# Oxytocin modulates respiratory heart rate variability through a hypothalamus–brainstem–heart neuronal pathway

**DOI:** 10.1038/s41593-025-02074-2

**Published:** 2025-10-20

**Authors:** Julie Buron, Ambre Linossier, Christian Gestreau, Fabienne Schaller, Roman Tyzio, Marie-Solenne Felix, Valéry Matarazzo, Muriel Thoby-Brisson, Françoise Muscatelli, Clément Menuet

**Affiliations:** 1 INMED, INSERM, Aix-Marseille University, Marseille, France; 2 INCIA, CNRS, Université de Bordeaux, Bordeaux, France; 3 Present Address: Department of Fundamental Neurosciences, University of Lausanne, Lausanne, Switzerland

**Keywords:** Neural circuits, Autonomic nervous system, Cardiovascular biology, Respiration

## Abstract

The variation in heart rate in phase with breathing is called respiratory heart rate variability (RespHRV). Relaxation and positive socio-emotional states can amplify RespHRV, yet the underlying mechanism remains largely unknown. Here we identify a hypothalamus–brainstem neuronal pathway in rodents through which oxytocin (OT) amplifies RespHRV during calming behavior. OT neurons from the caudal paraventricular nucleus in the hypothalamus regulate the activity of a subgroup of inhibitory neurons in the pre-Bötzinger complex, the brainstem nucleus that generates the inspiratory rhythm. Specifically, OT enhances the glycinergic input from OT-receptor-expressing neurons in the pre-Bötzinger complex to cardiac-innervating parasympathetic neurons in the nucleus ambiguus during inspiration. This leads to amplified respiratory modulation of parasympathetic activity to the heart, thereby enhancing RespHRV. We show that OT neurons participate in the restoration of RespHRV amplitude during recovery from stress in mice, indicating that OT acts centrally to regulate cardiac activity during a calming behavior.

## Abstract (web summary)

Buron et al. show that oxytocin enhances heart rate variability linked to breathing during recovery from stress. This calming and cardio-protective effect is produced through a hypothalamus–brainstem pathway for parasympathetic control of the heart.

## Main

Heart rate (HR) is highly variable. It is continuously regulated by the autonomic nervous system to maintain blood gas homeostasis and to produce physiological manifestations of emotional states, such as increased HR during stress and decreased HR during relaxation. A predominant source of HR variability originates from its coupling with respiratory activity, called RespHRV (also known as respiratory sinus arrhythmia)^1^, in which HR increases during inspiration and decreases during expiration. Physiologically, RespHRV optimizes cardiac efficiency, with increased cardiac activity occurring during inspiration when the myocardium receives oxygenated blood and vice versa during expiration, while maintaining physiological levels of arterial CO_2_ (refs. ^1–3^). Yet RespHRV has a broader impact. The amplitude of RespHRV—that is, the difference between maximal HR during inspiration and minimal HR during expiration—is larger in young individuals and in well-trained athletes, decreases with age and is dramatically reduced in hypertension and chronic heart failure^1–3^. Restoration of RespHRV amplitude in animal models of chronic heart failure, using a biofeedback heart pacemaker, induces large and persistent improvements in cardiac function^4,5^. RespHRV amplitude is also strongly modulated by emotional states. RespHRV is amplified during relaxation and positive socio-emotional states like parental bonding, and it is strongly decreased or even becomes negative in people with anxiety or depressive disorders, as well as in those with autism spectrum disorder^6–8^. Interestingly, the neuropeptide OT, widely studied for its relaxing, anxiolytic and pro-social effects^9^, has been found to amplify RespHRV after intranasal administration in humans^10^.

RespHRV is mainly generated by parasympathetic activity to the heart^1^, mediated by the vagus nerve, which originates from two brainstem nuclei: the nucleus ambiguus (nA) and the dorsal motor nucleus of the vagus nerve (DMV)^11^. Cardiac-innervating nA (nA^c^^ardiac^) neurons, whose activity decreases HR, are inhibited during inspiration and activated during expiration, thereby generating RespHRV^11–13^. DMV^c^^ardiac^ neurons are tonically active and can regulate mean HR (mHR)^11^. The inhibition of nA^c^^ardiac^ neuronal activity during inspiration probably arises from the adjacent pre-Bötzinger complex (preBötC), the neuronal group that generates the inspiratory rhythm^14–16^. In addition, pharmacological studies and the presence of a high density of OT fibers in the preBötC and nA areas (preBötC/nA) at the macroscopic level suggest that endogenous OT, which is primarily synthesized by neurons in the paraventricular (PVN) and supraoptic nuclei of the hypothalamus, could modulate the activity of preBötC and/or nA neurons^17–19^. Here, by using a variety of experimental approaches, we tested whether OT could amplify RespHRV through a central action on preBötC/nA neurons, with the dual aim of specifying the neuronal circuitry that generates RespHRV and identifying a mechanism for central RespHRV amplification with its behavioral relevance.

## Results

### PVN^OT^→preBötC/nA neurons amplify RespHRV and decrease mHR

**Fig. 1:**
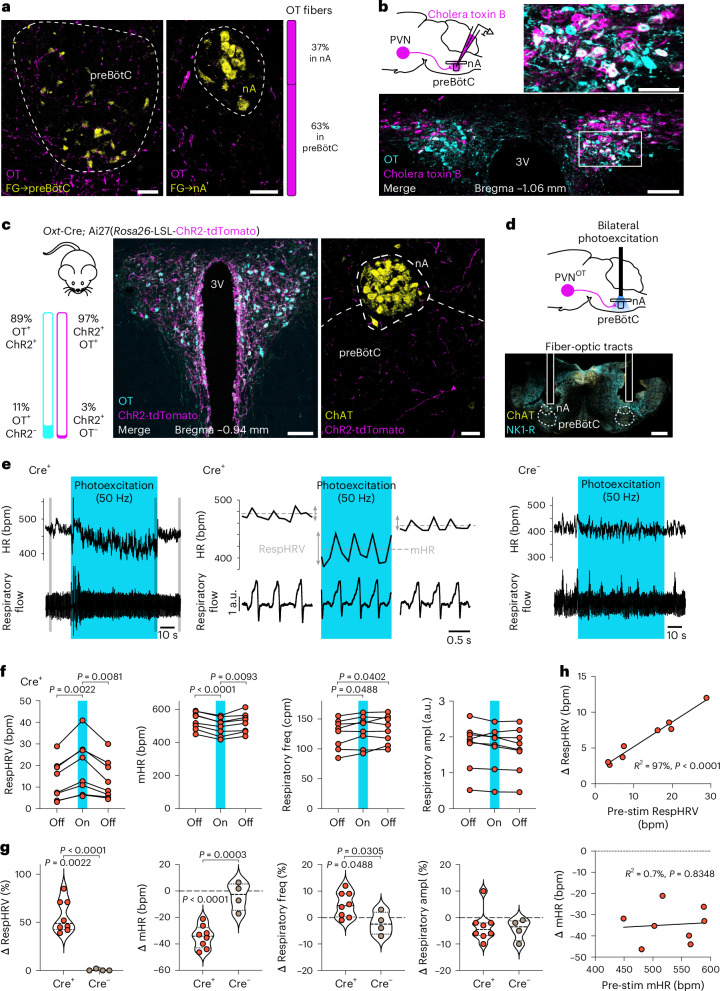
OT fibers from the caudal PVN to the preBötC/nA can amplify RespHRV, decrease mHR and increase respiratory frequency in adult freely moving mice at rest. **a**, Immunohistochemical labeling of OT fibers in the preBötC and nA, using FluoroGold (FG) retrograde tracing (Extended Data Fig. 1a,b) and classical anatomical landmarks^24^ (counts in *n* = 6 mice for nA, *n* = 6 mice for preBötC; Extended Data Fig. 1c,d). Scale bars, 50 µm. **b**, Retrograde tracing to localize OT neurons that project onto preBötC/nA neurons (*n* = 2, bilateral injections) (Extended Data Fig. 1e–g). 3V, third ventricle. Scale bars: large image, 100 µm; inset, 50 µm. **c**, ChR2-tdTomato expression in OT neurons (Extended Data Fig. 2a; *n* = 1) and in fibers in the preBötC/nA (*n* = 6 mice). ChAT, choline acetyltransferase. Scale bars: left, 100 µm; right, 50 µm. **d**, Strategy for bilateral photoexcitation of OT fibers in the preBötC/nA (verified a posteriori in *n* = 2 mice). NK1-R, neurokinin 1 receptor. Scale bar, 500 µm. **e**, Photoexcitation (1 min, 50 Hz, 10 ms pulses) of OT fibers in the preBötC/nA of freely moving mice (respiration measured by plethysmography; HR measured with ECG telemetry probes). Adult *Oxt*-Cre; Ai27(*Rosa26*-LSL-ChR2-tdTomato) (Cre^+^, *n* = 8) and Ai27(*Rosa26*-LSL-ChR2-tdTomato) female littermates (Cre^−^, *n* = 4) were used. Gray shaded areas on the left trace correspond to the recordings shown in the middle trace. bpm, beats per minute. **f**, Effects of the photoexcitation of OT fibers in the preBötC/nA of Cre^+^ mice on RespHRV, mHR, respiratory frequency (freq) and respiratory amplitude (ampl) (*n* = 8 mice). Repeated-measures one-way ANOVA, Tukey’s multiple comparison. cpm, cycles per minute; a.u., arbitrary units. **g**, Quantification of the relative effects (delta (Δ) in % or bpm for mHR) induced by photoexcitation of OT fibers in Cre^+^ (from absolute values shown in **f**) and Cre^−^ (absolute values shown in source data file) mice. Intra-group Δ changes (photoexcitation vs pre-photoexcitation, shown in **f**), repeated-measures one-way ANOVA with Tukey’s multiple comparison. Inter-group comparison (Cre^+^ vs Cre^−^), unpaired two-sided *t*-test. In violin plots, the dashed lines indicate the median and the dotted lines represent the quartiles. **h**, Correlation analysis between the Δ changes in RespHRV amplitude and mHR with the pre-photoexcitation values of these parameters. Pearson two-sided correlation analysis, simple linear regression plotted. Detailed statistics are presented in Supplementary Table 1. Source data

**Extended Data Fig. 1:**
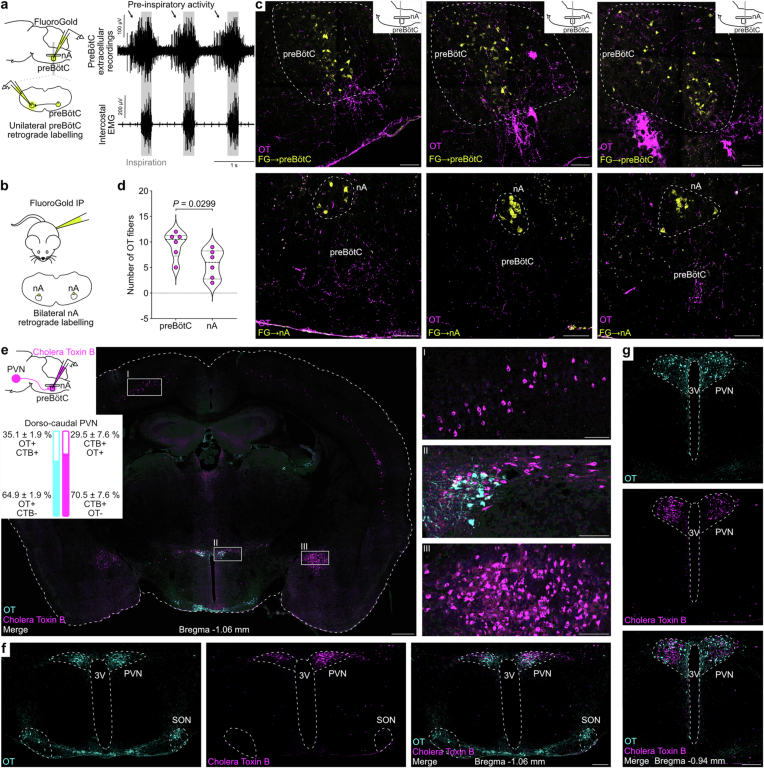
OT fibers are predominantly present in the preBötC compared to the nA, and OT neurons from the caudal PVN form a dense and regionally-specific cluster projecting to preBötC/nA neurons. **a**, FluoroGold injection in the preBötC unilaterally labels a subgroup of contralateral preBötC neurons, enabling delineation of the preBötC boundaries. PreBötC injections were guided by extracellular recordings made with the injection pipette, to localize the peak pre-inspiratory/inspiratory bursting activity on the rostral side of the neuronal cell column presenting inspiratory bursting activity. **b**, Intraperitoneal (IP) injection of the retrograde tracer FluoroGold labels nucleus ambiguus neurons. **c**, Coronal sections at three rostro-caudal levels (insets) with immunohistochemical labeling of OT fibers, and labeling of either contralaterally-projecting preBötC neurons (top, *n* = 6 mice) or nA neurons (bottom, *n* = 6 mice) with FluoroGold (FG). The preBötC contains a greater density of OT fibers compared to the nA. Scale bars, 100 µm. **d**, Average number of OT fibers counted in three brainstem sections (50 µm thick) per animal (*n* = 6 for preBötC, *n* = 6 for nA). Paired two-sided *t*-test for preBötC vs. nA. **e-g**, Bilateral injection of the retrograde tracer cholera toxin B in the preBötC/nA. OT neurons that project to the preBötC/nA are restricted to the dorso-caudal PVN. The most rostral level showing OT neurons co-labelled with cholera toxin B is at ~ Bregma -0.94 mm (**g**). The level with the highest proportion of OT neurons co-labelled with cholera toxin B is at ~ Bregma -1.06 mm (**e**, **f**). At this level, only three structures are labeled with cholera toxin B (**e**), the cortex (I in **e**), the PVN (II in **e**), and the central amygdala (III in **e**), similarly to what was shown previously using monosynaptic retrograde viral tracing from preBötC neurons^74^. Counts of the number of neurons expressing OT and/or cholera toxin B (CTB) (inset in **e**), expressed as relative proportions (%), were performed in the dorso-caudal PVN where cholera toxin B labeling was found (*n* = 2 mice). No cholera toxin B labeling was found in the supraoptic nucleus (SON) (**f**). Scale bars, 500 µm (whole section in **e**), 100 µm (I, II and III in **e**), 200 µm (**f**, **g**). Detailed statistics are presented in Supplementary Table 1. Source data

To determine whether preBötC and/or nA neurons receive OT projections, we immunolabeled OT fibers and labeled preBötC or nA neurons in adult wild-type mice (Fig. 1a and Extended Data Fig. 1a,b). The preBötC is defined by its anatomical location in the ventrolateral medulla oblongata and its function in generating the inspiratory rhythm^20–22^. It contains a network of heterogeneous neurons, with subgroups that are distinguishable by their developmental origin (for example, Dbx1^+^), neurochemistry (for example, glutamatergic, glycinergic, GABAergic), receptor expression (for example, µ-opioid receptor, neurokinin 1 receptor) or projection profile (for example, projection to the contralateral preBötC to synchronize the generation of the inspiratory rhythm^23^). To locate the preBötC, we used the addition of three identifying criteria: generation of the inspiratory rhythm, axonal projections to the contralateral preBötC and the classical anatomical landmarks for the preBötC (in particular, the shape of the inferior olives^24^ and the rostro-caudal position relative to the fourth ventricle). We made extracellular recordings to precisely localize the characteristic preBötC neurons that discharge during pre-inspiration and/or inspiration^14^ (all preBötC injections in this study were guided this way; Extended Data Fig. 1a), and we injected the retrograde neuronal tracer FluoroGold unilaterally at this coordinate. The contralateral preBötC was then defined by boundaries surrounding FluoroGold^+^ neurons at the classical preBötC anatomical landmarks (Extended Data Fig. 1a,c). On the other hand, nA neurons, including nA^cardiac^ neurons, form a nucleus of parasympathetic preganglionic neurons, predominantly sending their axons into the vagus nerve. nA neurons were labeled by intraperitoneal FluoroGold injection (Extended Data Fig. 1b,c). We found dense OT fibers throughout the rostro-caudal extent of the preBötC, some of which were in close apposition to contralaterally projecting preBötC neurons, but fewer OT fibers near nA neurons (Fig. 1a and Extended Data Fig. 1c,d). To identify which OT neurons project to preBötC/nA neurons, we injected the retrograde neuronal tracer, cholera toxin B, bilaterally into the preBötC/nA (Fig. 1b and Extended Data Fig. 1e–g). Among the few forebrain structures showing neurons labeled with cholera toxin B, a dense and specific cluster was present in the caudal dorso-lateral PVN. Here, ~30 % of the neurons projecting to the preBötC/nA were OT (PVN^OT^→preBötC/nA) neurons (Extended Data Fig. 1e). In addition, PVN^OT^→preBötC/nA neurons represented ~35 % of OT neurons in this region of the PVN.

**Extended Data Fig. 2:**
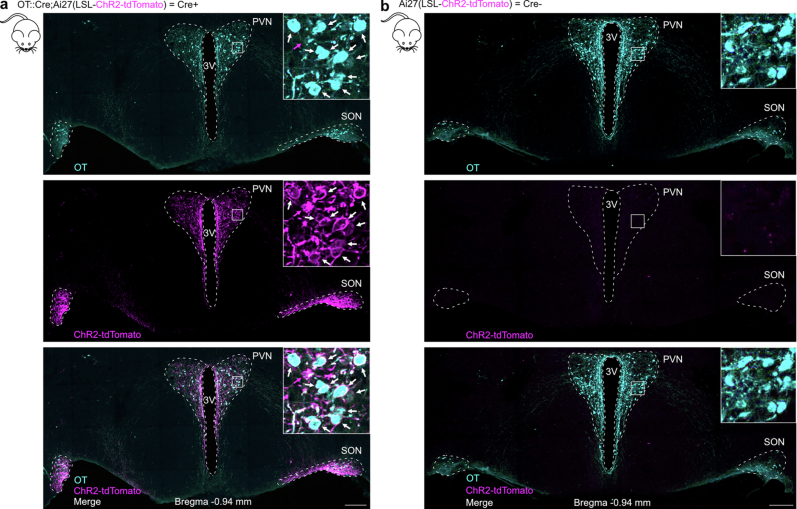
Specificity of ChR2-tdTomato expression in the OT neurons of OT::Cre;Ai27(LSL-ChR2-tdTomato) mice. **a**, The selective expression in OT neurons of the blue light gated cation channel ChR2 fused to the fluorescent protein tdTomato is induced by crossing OT::Cre mice with Ai27(LSL-ChR2-tdTomato) mice (*n* = 1 mouse). White arrows in insets indicate neurons counted as OT^+^ and ChR2-tdTomato^+^. A magenta arrow indicates a neuron counted as OT^−^ and ChR2-tdTomato^+^, therefore representing one of the rare neurons counted as non-specific expression (3% of OT^−^ ChR2^+^ neurons, Fig. 1c). This very low non-specific expression is likely overestimated by our count, because of the limited sensitivity of OT immunohistochemical labeling leading to an underestimation of the number of OT neurons^75^, and because we counted neurons showing faint OT labeling as OT^−^ neurons (such as the one indicated by the magenta arrow). Scale bar, 200 µm. **b**, Littermates that did not express the Cre recombinase (Cre^−^), but were genotyped positive for the LSL-ChR2-tdTomato transgene, obtained from crossings between OT::Cre mice with Ai27(LSL-ChR2-tdTomato) mice, were used as control mice. No ChR2-tdTomato expression was found in these mice (*n* = 1 mouse). Scale bar, 200 µm.

Next, we examined whether the PVN^OT^ fibers in the preBötC/nA can modulate cardiorespiratory function and, in particular, RespHRV. We used *Oxt*-Cre; Ai27(*Rosa26*-LSL-ChR2-tdTomato) adult female mice to optogenetically stimulate PVN^OT^ fibers in the preBötC/nA bilaterally, in vivo under freely moving conditions (Fig. 1c,d and Extended Data Fig. 2a,b). Cardiorespiratory functions were assessed with telemetry electrocardiography (ECG) and whole-body plethysmography. Prolonged photoexcitation (1 min, 50 Hz, 10 ms pulses, 10 mW mm^−2^) in animals during rest induced a strong and sustained amplification of RespHRV (+56%; from 13.2 ± 3.2 to 19.5 ± 4.3 beats per minute (bpm)) and a sustained decrease in mHR (−35 bpm; from 532 ± 18 to 497 ± 19 bpm) (Fig. 1e–g). However, it had only a moderate respiratory effect, inducing a small increase in respiratory frequency (+5%; from 125 ± 9 to 130 ± 9 cycles per minute) with no change in respiratory amplitude. There was no effect of photostimulation in control mice. These data show that PVN^OT^→preBötC/nA neurons can amplify RespHRV and decrease mHR in mice at rest.

### PVN^OT^ neurons release OT in the preBötC/nA to amplify RespHRV

**Fig. 2:**
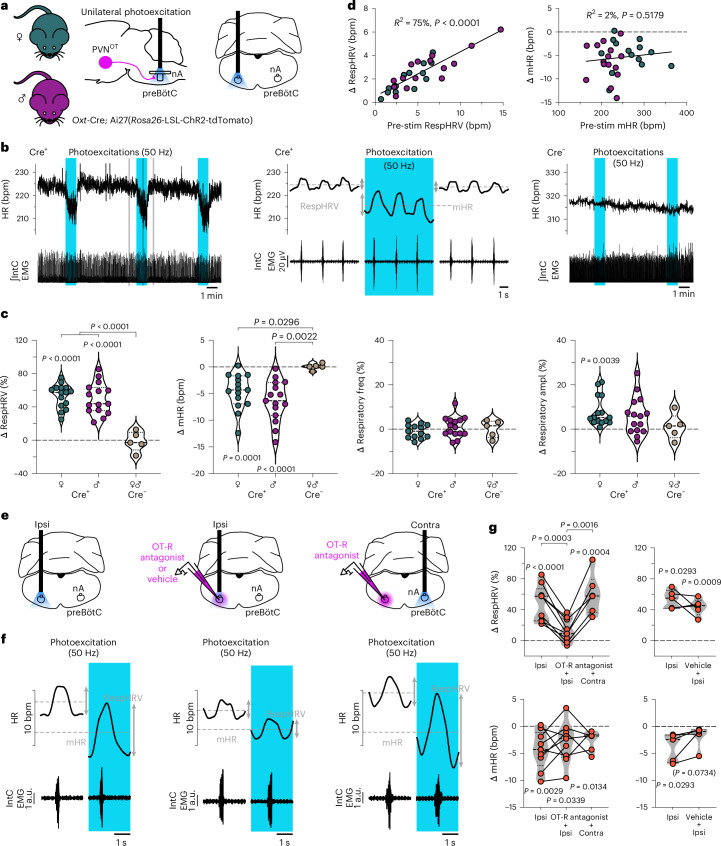
PVN^OT^ fibers can amplify RespHRV by releasing OT in the preBötC/nA in adult anesthetized mice. **a**–**c**, Unilateral photoexcitation of PVN^OT^ fibers in the preBötC/nA of anesthetized *Oxt*-Cre; Ai27(*Rosa26*-LSL-ChR2-tdTomato) adult female and male mice (**a**) induces RespHRV amplification and mHR decrease (**b**, representative traces; **c**, quantifications) (*n* = 15 Cre^+^ females, *n* = 15 Cre^+^ males, *n* = 5 Cre^−^ females and males). Intra-group Δ changes (photoexcitation vs pre-photoexcitation); repeated-measures one-way ANOVA with Tukey’s multiple comparison. Inter-group comparison (Cre^+^ females vs Cre^+^ males vs Cre^−^), one-way ANOVA with Tukey’s multiple comparison, except for respiratory amplitude, Kruskal–Wallis test with Dunn’s multiple comparison. In violin plots, the dashed lines indicate the median and the dotted lines represent the quartiles (**c**). Raw data are shown in Extended Data Fig. 3a. IntC EMG, intercostal electromyography. **d**, Correlation analysis between the Δ changes in RespHRV amplitude and mHR with the pre-photoexcitation values of these parameters. Pearson two-sided correlation analysis; simple linear regression plotted. **e**–**g**, Ipsilateral (ipsi) injection of a selective OT-R antagonist ((d(CH_2_)₅¹,Tyr(Me)²,Thr⁴,Orn⁸,des-Gly-NH_2_⁹)-Vasotocin; 200 nl at 1 µM) in the preBötC/nA (**e**) abolishes the photoexcitation-induced RespHRV amplification but not the mHR decrease (**f**, representative traces obtained in the conditions shown above in **e**; **g**, quantifications). Photoexcitation following ipsilateral vehicle injection (**g**, traces shown in Extended Data Fig. 4a), and photoexcitation in the contralateral (contra) preBötC/nA, induced similar RespHRV and mHR effects as ipsilateral pre-injection photoexcitation (*n* = 9 for ipsi and OT-R antagonist + ipsi; *n* = 6 for ipsi and OT-R antagonist + ipsi and OT-R antagonist + contra; *n* = 5 for ipsi and vehicle + ipsi). Intra-condition Δ changes (photoexcitation vs pre-photoexcitation), repeated-measures one-way ANOVA with Tukey’s multiple comparison. Inter-condition comparisons for OT-R antagonist (ipsi vs OT-R antagonist + ipsi vs OT-R antagonist + contra), repeated-measures mixed-effects analysis with the Geisser–Greenhouse correction, Tukey’s multiple comparison. Inter-condition comparisons for vehicle (ipsi vs vehicle + ipsi), paired two-sided *t*-test. Violin plots are represented in gray, dashed lines indicate the median and dotted lines represent the quartiles (**g**). Raw data for RespHRV and mHR, and data for the effects on respiratory frequency and respiratory amplitude are shown in Extended Data Fig. 3c–e. Localization of injection spots is shown in Extended Data Fig. 4b. Detailed statistics are presented in Supplementary Table 1. Source data

**Extended Data Fig. 3:**
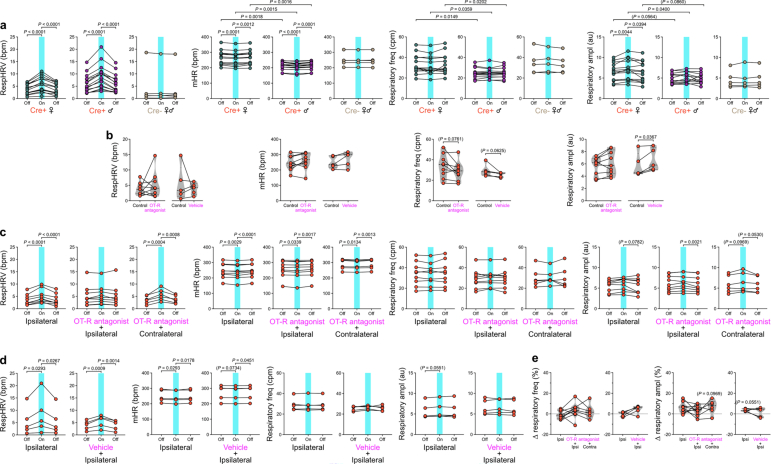
Individual data showing the respiratory and cardiac effects of the photoexcitation of PVN^OT^ fibers in the preBötC/nA, before and after injection of an OT-R antagonist. **a**, Individual data from the photoexcitation of OT fibers unilaterally in the preBötC/nA of adult anesthetized OT::Cre;Ai27(LSL-ChR2) mice (*n* = 15 Cre^+^ females, *n* = 15 Cre^+^ males, *n* = 5 Cre^−^ females and males). Cre^+^ female vs. male mice, pre-photoexcitation vs. photoexcitation vs. post-photoexcitation periods, repeated-measures two-way ANOVA, Sidak’s multiple comparison. Cre^−^ mice, repeated-measures one-way ANOVA, Tukey’s multiple comparison. freq, frequency; ampl, amplitude. **b**, In the mice subgroups (mixed sexes) shown in **a**, an OT-R antagonist ((d(CH_2_)₅¹,Tyr(Me)²,Thr⁴,Orn⁸,des-Gly-NH_2_⁹)-Vasotocin, 200 nl at 1 µM) or vehicle was then injected unilaterally in the preBötC/nA (*n* = 9 for OT-R antagonist injections, *n* = 5 for vehicle injections). Pre-injection (Control) vs. post-injection (OT-R antagonist or Vehicle), paired two-sided *t*-test or Wilcoxon two-sided matched-pairs signed rank test. **c**-**d**, Following the OT-R antagonist or vehicle injections, photoexcitations were repeated at the same unilateral site (OT-R antagonist + ipsilateral data in **c**, vehicle + ipsilateral in **d**, compared to pre-injections ipsilateral data) (*n* = 9 for ipsilateral and OT-R antagonist + ipsilateral in **c**, *n* = 5 for ipsilateral and vehicle + ipsilateral in **d**). In a subgroup of mice injected with the OT-R antagonist, photoexcitations in the contralateral preBötC/nA were made after the ipsilateral photoexcitations (*n* = 6, OT-R antagonist + contralateral in **c**). Repeated-measures one-way ANOVA, Tukey’s multiple comparison. **e**, Delta (Δ) changes in respiratory frequency and respiratory amplitude, from the pre-photoexcitation and photoexcitation data shown in **c** and **d**. Intra-condition Δ changes (photoexcitation vs. pre-photoexcitation), repeated-measures one-way ANOVA, Tukey’s multiple comparison. OT-R antagonist comparisons (ipsi vs. OT-R antagonist + ipsi vs. OT-R antagonist + contra), repeated-measures mixed-effects analysis with the Geisser-Greenhouse correction, Tukey’s multiple comparison. Vehicle comparisons (ipsi vs. vehicle + ipsi), paired two-sided *t*-test. Detailed statistics are presented in Supplementary Table 1. Source data

**Extended Data Fig. 4:**
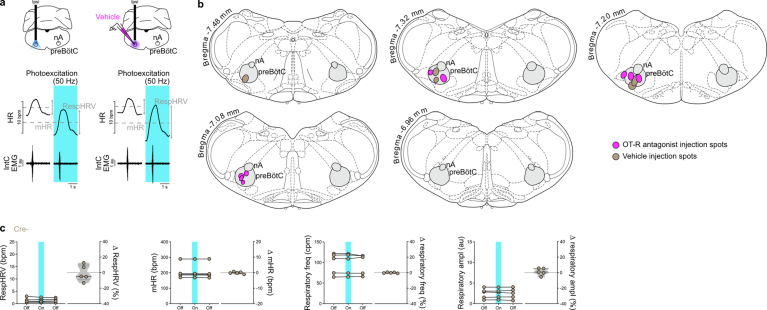
Raw traces for the photoexcitation of PVN^OT^ fibers in the preBötC/nA before and after vehicle injection, localization of the injection spots of the OT-R antagonist and vehicle, and control experiments for photoexcitations in the DMV. **a**, Vehicle injection in the preBötC/nA did not alter the effects induced by the photoexcitation of PVN^OT^ fibers (individual data in Fig. 2g and Extended Data Fig. 3d). **b**, Localization of the injection spots of a selective OT-R antagonist (200 nl) and of vehicle (200 nl) in the preBötC/nA, for data shown in Fig. 2e-g, Extended Data Fig. 3b-e, and Extended Data Fig. 5a-f. Each spot corresponds to a single injection in one mouse. **c**, In Cre^−^ mice, blue light illumination in the DMV induced no cardiorespiratory changes (*n* = 5 mice). Pre-photoexcitation vs. photoexcitation vs. post-photoexcitation periods, RM one-way ANOVA, Tukey’s multiple comparison, except for mHR, Friedman test, Dunn’s multiple comparison. Detailed statistics are presented in Supplementary Table 1. Source data

**Extended Data Fig. 5:**
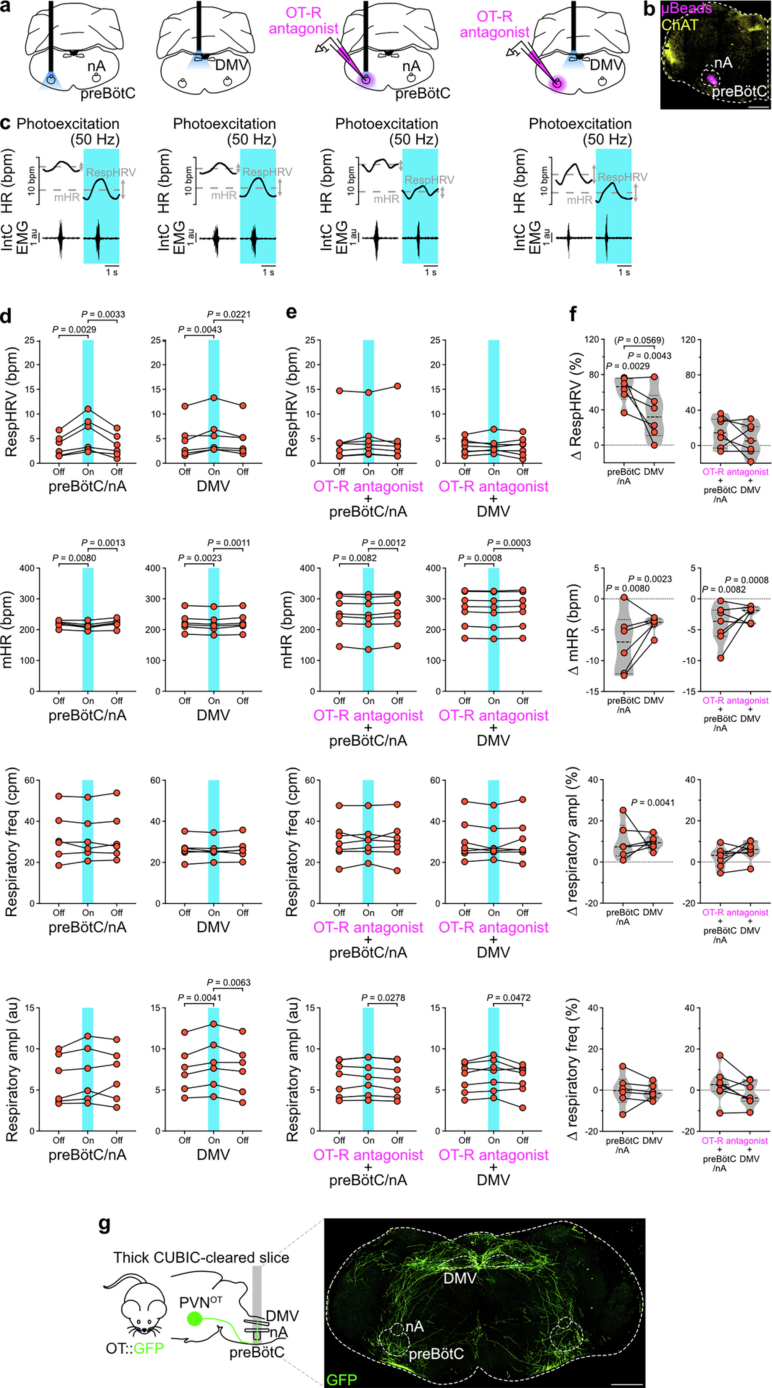
PVN^OT^ fibers can decrease mHR via DMV connectivity in adult anesthetized mice. **a**-**f**, Photoexcitation of PVN^OT^ fibers either in the preBötC/nA or in the DMV of anesthetized OT::Cre;Ai27(LSL-ChR2) adult mice induce the same effects, both before or after injection of the OT-R antagonist ((d(CH_2_)₅¹,Tyr(Me)²,Thr⁴,Orn⁸,des-Gly-NH_2_⁹)-Vasotocin, 200 nl at 1 µM) in the preBötC/nA (*n* = 6 for preBötC/nA and DMV before OT-R antagonist, *n* = 7 for preBötC/nA and DMV after OT-R antagonist). Traces in (**c**) were obtained in the conditions shown above in (**a**). **b**, Fluorescent microbeads in the injectate solutions enabled the localization of the injection spots for the OT-R antagonist or vehicle a posteriori (*n* = 9 mice for OT-R antagonist injections and *n* = 5 mice for vehicle injections, mapped in Extended Data Fig. 4b). Scale bar, 500 µm. **d**, repeated-measures one-way ANOVA, Tukey’s multiple comparison. **e**, repeated-measures one-way ANOVA, Tukey’s multiple comparison. **f**, Δ changes (photoexcitation vs. pre-photoexcitation), repeated-measures one-way ANOVA, Tukey’s multiple comparison. Comparisons before and after OT-R antagonist injection (preBötC/nA vs. DMV), paired two-sided *t*-test. **g**, CUBIC clearing of thick (1 mm) coronal brainstem slices at the level of the preBötC/nA in OT::GFP mice (*n* = 3 mice) shows OT fibers crossing both the preBötC/nA and the DMV. Scale bar, 500 µm. Detailed statistics are presented in Supplementary Table 1. Source data

OT fibers can release OT, but also other neurotransmitters such as glutamate^25,26^. To determine whether the cardiorespiratory effects evoked by photoexcitation of PVN^OT^ fibers are caused specifically by a release of OT in the preBötC/nA, we used anesthetized *Oxt*-Cre; Ai27(*Rosa26*-LSL-ChR2-tdTomato) adult mice and recorded ECG and inspiratory electromyogram (EMG). Unilateral photoexcitation of PVN^OT^ fibers in the preBötC/nA induced RespHRV amplification (+53%; from 3.8 ± 0.5 to 5.8 ± 0.8 bpm in females; +50%; from 6.1 ± 0.9 to 9.0 ± 1.3 bpm in males) and a modest yet consistent decrease in mHR (−5 bpm; from 269.5 ± 11.9 to 264.5 ± 12.04 bpm in females; −6 bpm; from 214.1 ± 6.6 to 207.8 ± 6.6 bpm in males) (Fig. 2a–c and Extended Data Fig. 3a). There was no change in respiratory frequency, but a tendency for an increase in inspiratory burst amplitude. Female and male mice had different baseline values for their cardiorespiratory parameters (Extended Data Fig. 3a) but similar profiles and magnitudes of their responses to the photoexcitation of PVN^OT^ fibers (Fig. 2c and Extended Data Fig. 3a). Consequently, unless specified, mixed sexes were used in all cohorts throughout the rest of this study. A selective OT receptor (OT-R) antagonist ((d(CH_2_)₅¹,Tyr(Me)²,Thr⁴,Orn⁸,des-Gly-NH_2_⁹)-Vasotocin)^25–27^ was then injected unilaterally in the preBötC/nA (Extended Data Figs. 4b and 5b), which caused no alteration in the cardiorespiratory parameters per se (Extended Data Fig. 3b). However, upon photoexcitation at the same site as the OT-R antagonist injection, the RespHRV amplification was almost abolished, while the modest decrease in mHR persisted (Fig. 2e–g and Extended Data Fig. 3c–e). Photoexcitation following injection of vehicle (Fig. 2e,g and Extended Data Figs. 3d,e and 4a,b) or photoexcitation in the preBötC/nA contralateral to the OT-R antagonist injection site (Fig. 2e–g and Extended Data Fig. 3c–e) induced similar cardiorespiratory effects as in the control pre-injection condition. These data show that PVN^OT^ neurons can induce RespHRV amplification by releasing OT in the preBötC/nA, whereas the mHR effect is dependent on a different mechanism.

### PVN^OT^ neurons decrease mHR by DMV connectivity

**Extended Data Fig. 6:**
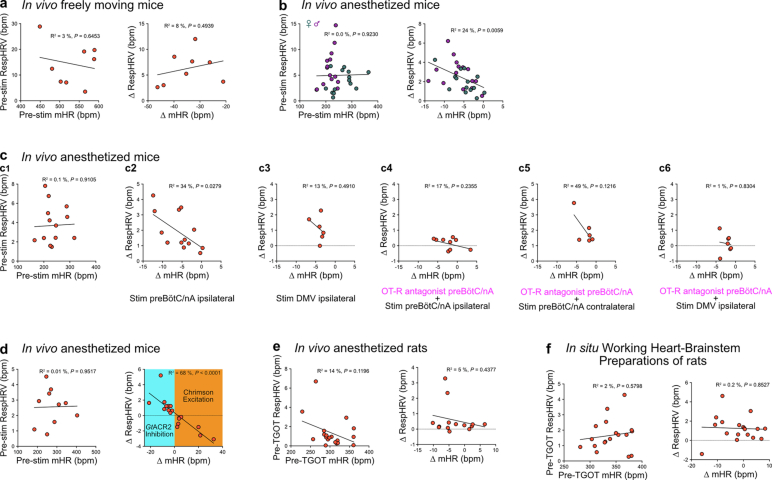
Correlation analysis between RespHRV and mHR in basal states and as delta changes in various experimental conditions. **a**, In freely moving mice (Fig. 1e-h), RespHRV amplitude and mHR are not correlated, in both pre-stim (basal) states and as Δ changes during photoexcitation of OT fibers in the preBötC/nA (*n* = 8 mice). **b**, In anaesthetized mice (Fig. 2a-d and Extended Data Fig. 4a), RespHRV amplitude and mHR are not correlated during pre-stim (basal) state, but their Δ changes during photoexcitation of OT fibers in the preBötC/nA are correlated (*n* = 15 female and *n* = 15 male mice). **c**, In a subgroup of mice shown in (**b**), in experimental conditions presented in Fig. 2e-g, Extended Data Fig. 3c-e and Extended Data Fig. 5a-f, RespHRV amplitude and mHR are not correlated during the pre-stim (basal) state (**c1**, *n* = 14 mice), but their Δ changes during photoexcitation of OT fibers in the preBötC/nA are correlated (**c2**, *n* = 14 mice). No correlation is evident in other experimental conditions (photostimulations in the DMV (**c3**, *n* = 6 mice), or photostimulations following OT-R antagonist injection in the preBötC/nA (**c4**, *n* = 9 mice; **c5**, *n* = 6 mice; **c6**, *n* = 7 mice)). **d**, In anaesthetized mice (Fig. 4b-d and Extended Data Fig. 7i-k, *n* = 10 mice), RespHRV amplitude and mHR are not correlated during the pre-stim (basal) state, but their Δ changes during photomodulations of preBötC^OT-R^ neurons are correlated (orange and blue backgrounds represent data from photoexcitations and photoinhibitions, respectively). **e**, In anesthetized rats (Fig. 6a-c), RespHRV amplitude and mHR are not correlated, in both the pre-TGOT (basal) state (*n* = 19 rats) and as Δ changes after TGOT injection in the preBötC (*n* = 14 rats). **f**, In rat in situ Working Heart-Brainstem Preparations (Fig. 6d-i), RespHRV amplitude and mHR are not correlated, in both the pre-TGOT (basal) state and as Δ changes after TGOT injection in the preBötC (*n* = 18 rats). **a**-**f**, Pearson two-sided correlation analysis, simple linear regression plotted. Detailed statistics are presented in Supplementary Table 1. Source data

Interestingly, the larger the RespHRV amplitude was before the photoexcitation of PVN^OT^ fibers in the preBötC/nA, the larger the delta in RespHRV amplification was during photoexcitation, with a strong correlation between the two parameters occurring in both the freely moving and anesthetized conditions (Figs. 1h and 2d). This observation suggests that OT probably acts in the preBötC/nA as a RespHRV gain amplifier. By contrast, the photoexcitation-induced decrease in mHR was not correlated with the pre-photoexcitation mHR level. Moreover, RespHRV amplitude and mHR were not correlated in the basal state, under both freely moving and anesthetized conditions, whereas the changes induced by photoexcitations of PVN^OT^ fibers in the preBötC/nA were correlated in the anesthetized condition but not in the freely moving condition (Extended Data Fig. 6a–c). In the anesthetized experiments, however, this correlation was lost during photoexcitations following injection of the OT-R antagonist in the preBötC/nA (Extended Data Fig. 6c). Together, these data further point towards a different mechanism underlying the central control of RespHRV and mHR, as proposed elsewhere^28^. OT was previously shown to act in the DMV to decrease mHR^29^. Photoexcitation of OT fibers in the DMV before and after injection of the OT-R antagonist in the preBötC/nA induced the same effects as the preBötC/nA photoexcitations, with RespHRV amplification only before the injection of the OT-R antagonist and mHR decrease both before and after the antagonist injection (Extended Data Fig. 5a–f). There was no effect of photostimulation in the DMV of control mice (Extended Data Fig. 4c). These findings suggest that some OT fibers may contact both the preBötC/nA and the DMV to amplify RespHRV and decrease mHR, respectively, as photoexcitations in either structure induce orthodromically and antidromically propagating action potentials to create the effects in both structures. This hypothesis is supported at the anatomical level, as tracing of OT fibers in CUBIC-cleared thick brainstem slices of *Oxt*-GFP mice containing the preBötC/nA and the DMV showed OT fibers crossing in both structures (Extended Data Fig. 5g), as also recently reported^30^. These data are therefore consistent with the conclusion that PVN^OT^ neurons differentially regulate cardiac activity, inducing RespHRV amplification through OTergic preBötC/nA connectivity and an mHR decrease through DMV connectivity.

### A subgroup of inhibitory preBötC neurons expresses the OT-R

**Fig. 3:**
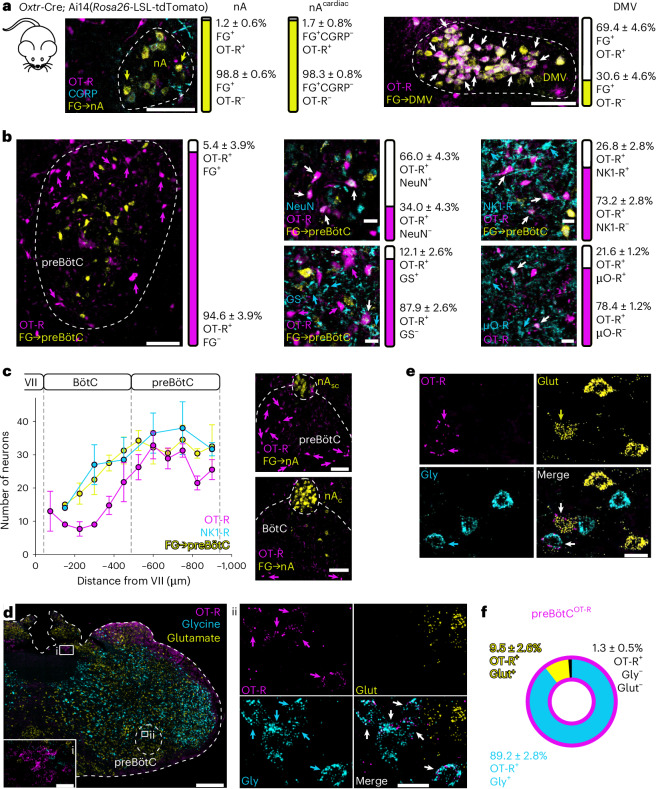
Anatomical and neurochemical characterization of OT-R^+^ cells in the preBötC/nA. **a**, Crossing of *Oxtr*-Cre mice with Ai14(*Rosa26*-LSL-tdTomato) mice to express tdTomato only in OT-R^+^ cells. FG was injected intraperitoneally in adult mice to label nA and DMV neurons (*n* = 6 mice for nA counts; *n* = 5 mice for DMV counts). nA^c^^ardiac^ neurons were detected as nA FG^+^ neurons that do not express the calcitonin gene-related peptide (CGRP^−^). Arrows indicate labeled neurons, with white arrows showing double-labeled OT-R^+^FG^+^ neurons. Individual data are shown in Extended Data Fig. 7a. Scale bars, 100 µm. **b**, Unilateral preBötC boundaries were mapped using contralateral preBötC FG injection (Extended Data Fig. 1a,c) and using classical anatomical landmarks of the preBötC location^24^. PreBötC^OT-R^ (OT-R^+^) cells were tested for their contralateral projection phenotype (FG^+^; *n* = 4 mice), neuronal vs astrocytic phenotype (NeuN^+^ vs glutamine synthetase (GS)^+^, respectively; *n* = 4 mice), and for the expression of two classical markers of preBötC neurons (NK1-R^+^, *n* = 4 mice; µ-opioid receptor (µO-R^+^), *n* = 6 mice). Arrows indicate labeled neurons, with white arrows showing double labeling between OT-R^+^ and the cyan marker. Individual data are shown in Extended Data Fig. 7b,c. Scale bar for preBötC^OT-R^ and FG only, 100 µm; other scale bars, 20 µm. **c**, OT-R^+^ cells are densely present in the preBötC, and minimally present in the adjacent Bötzinger complex (BötC) (*n* = 4 mice, data presented as means; error bars, s.e.m.). nA_sc_, semi-compact formation of the nA; nA_c_, compact formation of the nA. Scale bars, 100 µm. **d**–**e**, RNAscope in situ hybridization in adult wild-type mice to detect RNA transcripts encoding the expression of the OT-R (highest expression density in the DMV (**d**(i))) and of a glycinergic or glutamatergic phenotype (*n* = 4 mice). Representative examples from the preBötC of two mice, showing OT-R transcript expression (magenta arrows) only in glycinergic neurons (**d**(ii), cyan arrows), or in both a glycinergic and a glutamatergic neuron (**e**, cyan and yellow arrows, respectively). White arrows indicate dually labelled neurons. Scale bars: full image in **d**, 200 µm; **d**(i) inset, 50 µm; **d**(ii) inset, 20 µm; **e**, 20 µm. **f**, Relative quantification of the phenotype of preBötCOT-R neurons. Individual data are shown in Extended Data Fig. 7d. Detailed statistics are presented in Supplementary Table 1. Source data

**Extended Data Fig. 7:**
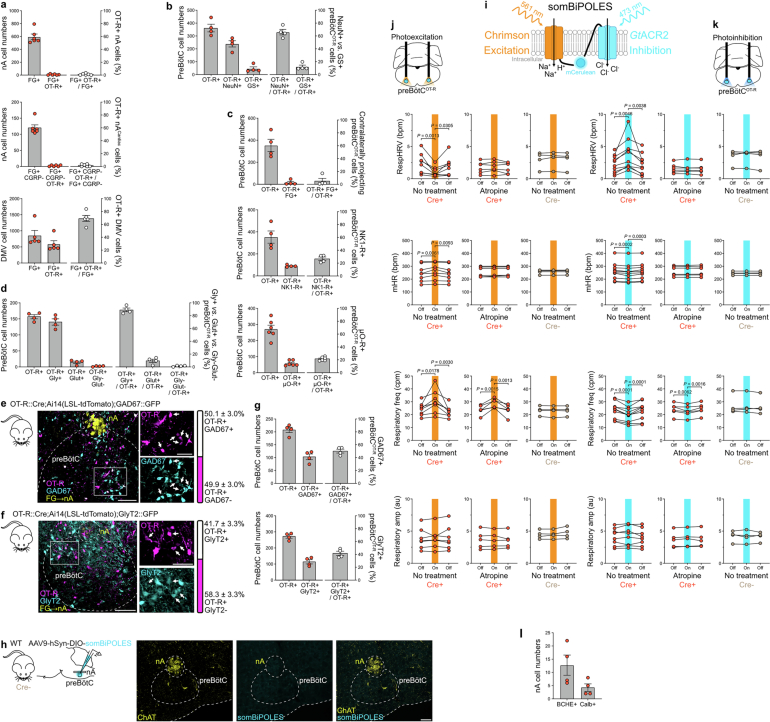
Individual data for the anatomo-functional characterization of OT-R^+^ cells in the preBötC/nA, and absence of somBiPOLES expression in Cre^−^ mice. **a-c**, Individual data for the averaged counts shown in Fig. 3a-b. Each point represents the total number of cells counted unilaterally in one mouse (*n* = 6 mice for nA counts and *n* = 5 mice for DMV counts in **a**; *n* = 4 mice in **b**; *n* = 4 mice for FG^+^ counts, *n* = 4 mice for NK1-R^+^ counts and *n* = 6 mice for µO-R^+^ counts in **c**; data presented as mean ± s.e.m. ). **d**, Individual data for the averaged counts shown in Fig. 3f. Each point represents the total number of cells counted bilaterally in three brainstem coronal sections containing the preBötC of each mouse (*n* = 4 mice, data presented as mean ± s.e.m.). **e-f**, Crossing of OT-R::Cre;Ai14(LSL-tdTomato) mice with GAD67::GFP mice (**e**) or GlyT2::GFP mice (**f**) to label OT-R^+^ cells (tdTomato^+^) and GABAergic or glycinergic neurons (GFP^+^), respectively. Scale bars: full images, 100 µm; insets, 50 µm. **g**, Individual data for the averaged counts shown in **e** and **f** (*n* = 4 mice for GABAergic counts, *n* = 4 mice for glycinergic counts, data presented as mean ± s.e.m.). **h**, Absence of somBiPOLES expression in Cre^−^ mice following viral injection in the preBötC (*n* = 4 mice). Scale bar, 100 µm. **i**, Schematic representation of the somBiPOLES construct. **j-k**, Individual data from the photoexcitation (**j**) and photoinhibition (**k**) of preBötC^OT-R^ neurons bilaterally in adult anesthetized mice (*n* = 8 Cre^+^ mice for photoexcitation and *n* = 10 Cre^+^ mice for photoinhibition with no treatment, *n* = 6 Cre^+^ mice for photoexcitation/photoinhibition after atropine, *n* = 4 Cre^−^ mice). Repeated-measures one-way ANOVA, Tukey’s multiple comparison. The pre-photomodulation and photomodulation data were used to calculate the delta changes for each measured parameter represented in Fig. 4c and d. freq, frequency; ampl, amplitude. **l**, Number of butyrylcholinesterase^+^ (BCHE + ) and calbindin^+^ (Calb + ) nA^Ccardiac^ neurons counted bilaterally (*n* = 4 mice, data presented as mean ± s.e.m.) with viral-mediated expression of tdTomato and synaptophysin-eGFP in preBötC^OT-R^ neurons (Fig. 4e-g). Detailed statistics are presented in Supplementary Table 1. Source data

So far, our data show that PVN^OT^ fibers can amplify RespHRV by releasing OT in the preBötC/nA, probably acting on preBötC rather than nA neurons, given that most PVN^OT^ projection fibers were found in the preBötC (Fig. 1a and Extended Data Fig. 1c,d). To identify the cells modulated by OT to induce RespHRV amplification, we used *Oxtr*-Cre; Ai14(*Rosa26*-LSL-tdTomato) adult mice to label OT-R^+^ cells (Fig. 3a–c and Extended Data Fig. 7a–c). In the preBötC/nA, very few OT-R^+^ neurons are nA neurons (7.6 ± 3.8 neurons on each side) or nA^cardiac^ neurons (2.0 ± 0.9 neurons on each side), identified as nA neurons not expressing the calcitonin gene-related peptide^31,32^; by contrast, most DMV neurons are OT-R^+^ (587.2 ± 106.4 neurons on each side) (Fig. 3a and Extended Data Fig. 7a). OT-R^+^ cells in the preBötC/nA are predominantly neurons (66.0 ± 4.3% neurons vs 12.1 ± 2.6% astrocytes), with qualitatively small somata (Fig. 3b and Extended Data Fig. 7b). They are present mainly within the anatomical boundaries of the preBötC, expressing classical markers of the preBötC neuronal population, including the expression of neurokinin 1 and µ-opioid receptors (Fig. 3b and Extended Data Fig. 7c). However, few contralaterally projecting preBötC neurons were OT-R^+^. PreBötC^OT-R^ neurons represent a population of ~700 neurons bilaterally (357.5 ± 4.4 neurons on each side). They are localized throughout the preBötC, with no specific clustering, but appear as a specific population within the respiratory cell column, as few OT-R^+^ neurons were found in the adjacent Bötzinger Complex (Fig. 3c). These data show that the OT-induced amplification of RespHRV is probably mediated via PVN^OT^→preBötC^OT-R^ connectivity.

A cumulative body of work suggests that the inspiratory component of RespHRV is generated by inhibitory, possibly glycinergic, preBötC neurons^12–14,16,33^. Moreover, OT has been shown to act selectively on inhibitory neurons in neuronal networks of the hippocampus and the amygdala, to amplify the gain of inhibitory synaptic connectivity^27,34,35^. We assessed the inhibitory glycinergic or excitatory glutamatergic phenotype of preBötC^OT-R^ neurons in adult wild-type mice, using RNAscope in situ hybridization (Fig. 3d–f and Extended Data Fig. 7d). We found that 89% of preBötC^OT-R^ neurons are glycinergic, 10% are glutamatergic and 1% remained uncharacterized. Qualitatively, preBötC^OT-R;glycine^ neurons represent only a small fraction of all preBötC^glycine^ neurons, present mostly in the dorsal part of the preBötC. In addition, the density of OT-R RNA transcripts is sparser in preBötC^OT-R^ neurons than in DMV^OT-R^ neurons (Fig. 3d(i),(ii)). We further assessed the inhibitory phenotype of preBötC^OT-R^ neurons using immunolabeling in *Oxtr*-Cre; Ai14(*Rosa26*-LSL-tdTomato); *Gad67*-GFP and *Oxtr*-Cre; Ai14(*Rosa26*-LSL-tdTomato); *Glyt2*-eGFP adult mice (Extended Data Fig. 7e–g). In these triple transgenic mice, 50% of preBötC^OT-R^ neurons were found to be GABAergic (Extended Data Fig. 7e,g) and 42% were glycinergic (Extended Data Fig. 7f,g). Overall, preBötC^OT-R^ neurons express a predominantly inhibitory glycinergic and/or GABAergic phenotype.

### PreBötC^OT-R^ neurons form a cardiorespiratory hub that controls RespHRV

**Fig. 4:**
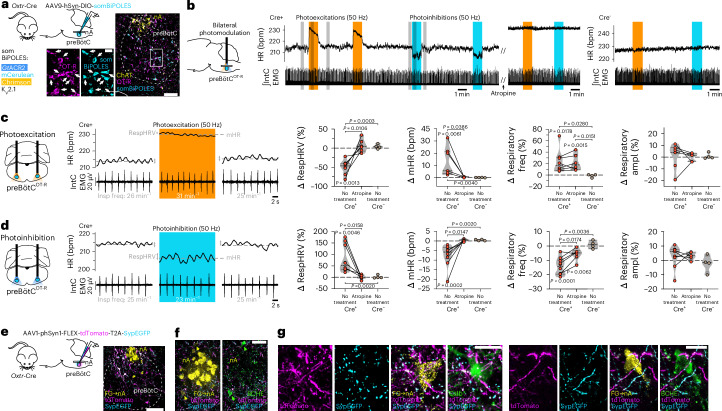
PreBötC^OT-R^ neurons control RespHRV amplitude and project onto nA^c^^ardiac^ neurons. **a**, Viral-mediated expression of the somBiPOLES optogenetic construct in preBötC^OT-R^ neurons of *Oxtr*-Cre mice (Extended Data Fig. 7i) (*n* = 10 mice). Viral injections in Cre^−^ mice did not induce somBiPOLES expression (Extended Data Fig. 7h; *n* = 4 mice). An anti-OT-R antibody was used for immunohistochemical labeling. Scale bars: full image, 100 µm; inset, 20 µm. **b**, Effects of bilateral photomodulations of preBötC^OT-R^ neurons in an anesthetized mouse (Cre^+^) on HR and inspiratory activity (IntC EMG), before and after intraperitoneal injection of the muscarinic receptor antagonist atropine (10 mg kg^−1^, 600 µl), and compared to a control (Cre^−^) mouse. **c**,**d**, Expanded traces from the gray shaded areas in **b**, and individual data showing bidirectional cardiorespiratory effects of photoexcitation (**c**) vs photoinhibition (**d**) of preBötC^OT-R^ neurons (*n* = 8 Cre^+^ mice for photoexcitation and *n* = 10 Cre^+^ mice for photoinhibition without treatment; *n* = 6 Cre^+^ mice for photoexcitation or photoinhibition before vs after atropine; *n* = 4 Cre^−^ mice). Intra-group Δ changes (photoexcitation vs pre-photoexcitation), repeated-measures one-way ANOVA with Tukey’s multiple comparison except for Cre^−^ RespHRV photoinhibition, Friedman test with Dunn’s multiple comparison. Cre^+^ before and after atropine comparison, paired two-sided *t*-test except for respiratory amplitude photoexcitation, Wilcoxon two-sided matched-pairs signed rank test. Inter-group comparison (Cre^+^ no treatment vs Cre^+^ atropine vs Cre^−^ no treatment), Kruskal–Wallis test with Dunn’s multiple comparison. Violin plots are represented in gray, dashed lines indicate the median and dotted lines represent the quartiles. Raw data are shown in Extended Data Fig. 7j,k. **e**, Viral-mediated expression of tdTomato and synaptophysin-enhanced GFP (SypEGFP) in preBötC^OT-R^ neurons (*n* = 4 mice). Scale bar, 100 µm. **f**,**g**, PreBötC^OT-R^ neurons project to (tdTomato^+^ fibers) and make putative pre-synaptic contacts with (SypEGFP^+^ puncta) nA^c^^ardiac^ neurons (FG^+^BCHE^+^ and FG^+^Calb^+^ neurons; Extended Data Fig. 7l; *n* = 4 mice). BCHE, butyrylcholinesterase; Calb, calbindin. Scale bars: in **f**, 50 µm; in **g**, 20 µm. Detailed statistics are presented in Supplementary Table 1. Source data

Our data show that PVN^OT^ neurons can amplify RespHRV by releasing OT onto preBötC^OT-R^ neurons, which express a predominantly inhibitory phenotype, and not by directly modulating the output nA^c^^ardiac^ neurons. PreBötC neurons are defined as a group of neurons generating the inspiratory rhythm^20–22^. To determine the functional role of preBötC^OT-R^ neurons for cardiorespiratory activities, we injected a Cre-dependent adeno-associated virus (AAV) bilaterally in the preBötC of adult *Oxtr*-Cre mice to express the somBiPOLES construct that enables bidirectional optogenetic modulation^36^ (Fig. 4a and Extended Data Fig. 7h,i). Prolonged photoexcitation of preBötC^OT-R^ neurons in anesthetized mice induced a rapid and sustained decrease in RespHRV amplitude (−49.9 ± 6.6%), an increase in mHR (+12.7 ± 4.5 bpm) and an increase in respiratory frequency (+23.2 ± 7.3%) (Fig. 4b,c and Extended Data Fig. 7j). Prolonged photoinhibition of the same neurons induced the opposite effects, causing a rapid and sustained increase in RespHRV amplitude (+77.2 ± 18.5%), a decrease in mHR (−7.5 ± 1.8 bpm) and a decrease in respiratory frequency (−14.3 ± 1.8%) (Fig. 4b,d and Extended Data Fig. 7k). There was no effect on respiratory amplitude or on all parameters in control mice. Interestingly, these bidirectional effects on RespHRV amplitude indicate that preBötC^OT-R^ neurons continuously set an ongoing level of RespHRV amplitude. The large variations in the amplitude of these bidirectional effects indicate that external modulators like OT can induce strong effects on RespHRV amplitude by acting on preBötC^OT-R^ neurons. Moreover, preBötC^OT-R^ neurons are not part of the preBötC inspiratory rhythmogenic subgroup, given that the photoinhibition of preBötC^OT-R^ neurons did not terminate respiratory activity, in contrast to an inactivation of preBötC rhythmogenic neurons^14,37,38^. Therefore, preBötC^OT-R^ neurons form a core cardiorespiratory hub within the preBötC that controls RespHRV amplitude.

### PreBötC^OT-R^ neurons project onto nA^c^^ardiac^ neurons

Intraperitoneal injection of the muscarinic receptor antagonist atropine, which blocks parasympathetic activity to the heart, blocked the effects of optogenetic modulations of preBötC^OT-R^ neurons on RespHRV and mHR, but not the respiratory effects (Fig. 4b–d and Extended Data Fig. 7j,k). This finding shows that preBötC^OT-R^ neurons modulate cardiac activity through the modulation of cardiac parasympathetic activity; probably through nA^c^^ardiac^ neurons because they are the sole cardiac parasympathetic preganglionic neurons that are respiratory modulated^11–13,16^. Moreover, the predominantly inhibitory nature of preBötC^OT-R^ neurons (Fig. 3d–f and Extended Data Fig. 7d–g), in addition to the direction of mHR effects during the photomodulation of preBötC^OT-R^ neurons (increased mHR during photoexcitation, decreased mHR during photoinhibition) (Fig. 4b–d and Extended Data Fig. 7j,k), strongly suggests that the inputs from preBötC^OT-R^ neurons to cardiac parasympathetic preganglionic neurons are monosynaptic. To determine whether preBötC^OT-R^ neurons project directly onto nA^c^^ardiac^ neurons, we injected a Cre-dependent AAV in *Oxtr*-Cre mice to express tdTomato and synaptophysin-GFP in preBötC^OT-R^ neurons, and we analyzed the preBötC^OT-R^→nA^c^^ardiac^ axonal projections (tdTomato^+^) and putative pre-synaptic contacts (synaptophysin-GFP^+^) (Fig. 4e–g). To specifically identify the nA^c^^ardiac^ neurons, we used intraperitoneal FluoroGold injection to label all nA neurons as well as selective immunolabeling of nA^c^^ardiac^ neurons based on the recent identification of two specific markers, calbindin and butyrylcholinesterase^32^ (Fig. 4f,g), and the confirmation of a third marker with the absence of expression of the calcitonin gene-related peptide^31,32^. We found dense preBötC^OT-R^ axonal projections throughout the rostro-caudal extent of the nA. All identified nA^c^^ardiac^ neurons (51 butyrylcholinesterase^+^ and 17 calbindin^+^ nA^c^^ardiac^ neurons bilaterally in four mice; Extended Data Fig. 7l) received projections and putative pre-synaptic contacts from preBötC^OT-R^ neurons (Fig. 4g). Therefore, these data are consistent with a direct monosynaptic connectivity from preBötC^OT-R^ neurons to nA^c^^ardiac^ neurons.

### PreBötC^OT-R^ neurons are inspiratory bursting and excited by OT

**Fig. 5:**
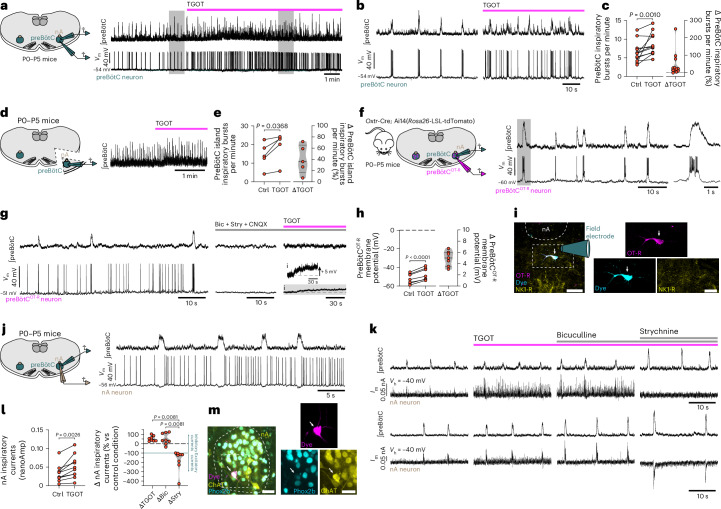
TGOT increases the frequency of preBötC inspiratory bursts, depolarizes preBötC^OT-R^ neurons that are inspiratory bursting and amplifies the glycinergic post-synaptic currents occurring in nA neurons during inspiratory bursts in rhythmic slices in vitro*.* **a**–**c**, TGOT (0.5 µM) increases the frequency of preBötC inspiratory bursts in rhythmic preBötC slices from neonatal wild-type mice (*n* = 12 mice; ⎰preBötC, integrated extracellular recordings; *V*_m_, whole-cell current-clamp recordings). Gray shaded areas in (**a**) are expanded in (**b**). Wilcoxon two-sided matched-pairs signed rank test (**c**). The violin plot is represented in gray, dashed line indicates the median and dotted lines represent the quartiles (**c**). **d**,**e**, TGOT (0.5 µM) increases the frequency of preBötC inspiratory bursts in preBötC ‘island’ preparations (*n* = 5 mice; **d**, representative trace; **e**, quantifications). Paired two-sided *t*-test. The violin plot is represented in gray, dashed line indicates the median and dotted lines represent the quartiles (**e**). **f**–**h**, PreBötC^OT-R^ neurons recorded in rhythmic preBötC slices from neonatal *Oxtr*-Cre; Ai14(*Rosa26*-LSL-tdTomato) mice display an inspiratory bursting pattern of activity (**f**), and TGOT (0.5 µM) depolarizes preBötC^OT-R^ neurons following the blockade of fast synaptic transmission (bicuculline (Bic), 10 µM; strychnine (Stry), 5 µM; CNQX, 20 µM; *n* = 7 mice) (**g**, representative traces; **h**, quantifications). Paired two-sided *t*-test. The violin plot is represented in gray, dashed line indicates the median and dotted lines represent the quartiles (**h**). **i**, Example of a recorded preBötC^OT-R^ neuron (dye^+^ and OT-R;tdTomato^+^, white arrows) located in the NK1-R-expressing preBötC region (*n* = 4 neurons recorded that were labeled and localized a posteriori). Scale bars: full image, 40 µm; inset, 20 µm. **j**, All respiratory-modulated nA neurons recorded (*n* = 10 wild-type mice) were hyperpolarized during the preBötC inspiratory bursts, causing inhibition of their spiking activity. **k**,**l**, nA neurons showed volleys of inhibitory currents during preBötC inspiratory bursts in the control condition (*I*_m_, whole-cell voltage-clamp recordings; *V*_h_, −40 mV holding voltage), which were amplified by TGOT (0.5 µM) application (*n* = 10 mice; **k**, representative traces; **l**, quantifications). During TGOT application, bicuculline (10 µM) application had no effect, while strychnine (5 µM) application either abolished all currents (*n* = 5 mice, top traces in **k**) or exposed the presence of volleys of excitatory currents during preBötC inspiratory bursts (*n* = 3 mice, bottom traces in **k**, further characterized in Extended Data Fig. 8d). Control vs TGOT, paired two-sided *t*-test. Δ changes following TGOT, bicuculline and strychnine applications, Friedman test with Dunn’s multiple comparison. Violin plots are represented in gray, the dashed lines indicate the median, the dotted lines represent the quartiles and the green line represents the limit above which the recorded currents are inhibitory and under which they are excitatory (**l**). **m**, Example of a recorded nA neuron (arrows indicate the dye^+^ recorded neuron that is Phox2b^+^ and ChAT^+^; *n* = 5 neurons recorded that were labeled and localized a posteriori). Scale bars: full image, 40 µm; inset, 20 µm. Detailed statistics are presented in Supplementary Table 1. Source data

**Extended Data Fig. 8:**
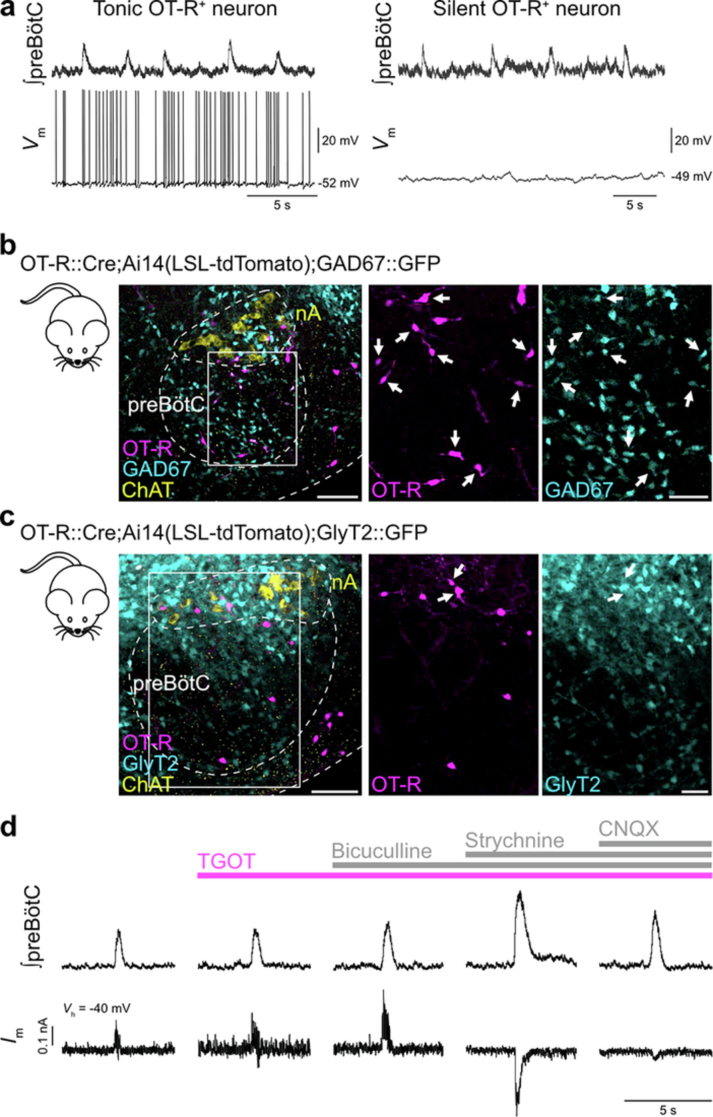
OT-R^+^ neurons outside of the preBötC do not discharge in phase with preBötC inspiratory bursts, the neurochemical phenotype of preBötC^OT-R^ neurons is similar in P0 mice compared to adults, and characterization of the excitatory post-synaptic currents in nA neurons during inspiratory bursts. **a**, All OT-R^+^ neurons recorded outside of the preBötC (*V*_m_, whole-cell current-clamp recordings) showed activity patterns that were not in phase with the preBötC inspiratory-phase discharging population activity (*n* = 6 neurons, 3 tonic and 3 silent). **b-c**, In P0 triple transgenic mice, preBötC^OT-R^ neurons show the same predominantly GABAergic (**b**) or glycinergic (**c**) phenotype as in adult mice (Extended Data Fig. 7e-g; arrows indicate OT-R and GAD67 or GlyT2 co-labelling). Scale bars: full images, 100 µm; insets, 50 µm. **d**, Same experiment as in Fig. 5k on a rhythmic preBötC slice from a neonatal wild-type mouse, here showing successive effects induced by the application of TGOT (0.5 µM), bicuculline (GABA_A_ receptor antagonist, 10 µM), strychnine (glycine receptors antagonist, 5 µM), and CNQX (AMPA/kainate receptor antagonist, 20 µM), on preBötC population activity (⎰preBötC, integrated extracellular recordings) and on the currents recorded from a respiratory-modulated nA neuron that showed volleys of excitatory currents during inspiratory bursts following strychnine application (*I*_m_, whole-cell voltage-clamp recordings; *V*_h_, -40 mV holding voltage). These excitatory currents were blocked by CNQX application, showing that they are induced by glutamatergic transmission (*n* = 3 mice).

Our results thus far show that preBötC^OT-R^ neurons express a predominantly inhibitory glycinergic and/or GABAergic phenotype, make putative monosynaptic contacts with nA^c^^ardiac^ neurons and regulate cardiorespiratory functions and RespHRV amplitude. It is therefore probable that the amplification of RespHRV by OT, identified by our optogenetic stimulations of PVN^OT^→preBötC fibers, is the result of an amplification of the preBötC^OT-R^→nA^c^^ardiac^ inhibitory connectivity during inspiration. To test this possibility, we used brainstem slices from newborn mice (postnatal days 0–5 (P0–P5)) that contain the preBötC/nA structures, and in which preBötC neurons continue spontaneously to generate rhythmic inspiratory bursting activity^20,39,40^. We first performed extracellular recordings of preBötC population activity, simultaneously with whole-cell current-clamp recordings of inspiratory bursting preBötC neurons (Fig. 5a,b). Bath application of the selective OT-R agonist [Thr^4^,Gly^7^]-OT (TGOT)^27,41,42^ induced an increase in preBötC inspiratory bursting frequency (+47.0 ± 20.4%) along with an increase in preBötC network excitability, as shown by the appearance of spiking activity between ongoing inspiratory bursts (Fig. 5a–c). To confirm that these effects are mediated specifically by the action of TGOT on preBötC^OT-R^ neurons, we used isolated preBötC ‘island’ preparations^43^, in which TGOT still increased preBötC inspiratory bursting frequency (+40.1 ± 11.5%) (Fig. 5d,e). We then assessed the pattern of activity of preBötC^OT-R^ neurons using *Oxtr*-Cre; Ai14(*Rosa26*-LSL-tdTomato) mice (Fig. 5f–i). All preBötC^OT-R^ neurons recorded (*n* = 15 neurons from nine mice) showed an inspiratory bursting pattern of activity, with some neurons active only during inspiratory bursts (Fig. 5f), while others also showed tonic activity during the expiratory phases (Fig. 5g). TGOT application following the blockade of fast synaptic transmission depolarized all preBötC^OT-R^ neurons recorded (+4.9 ± 0.5 mV, *n* = 7 neurons from seven mice; Fig. 5g,h). OT-R^+^ neurons outside of the preBötC were also recorded, none of which showed inspiratory bursting activity (*n* = 6 neurons, three tonic and three silent; Extended Data Fig. 8a). Immunolabeling of preBötC^OT-R^ neurons in newborn *Oxtr*-Cre; Ai14(*Rosa26*-LSL-tdTomato); *Gad67*-GFP and *Oxtr*-Cre; Ai14(*Rosa26*-LSL-tdTomato); *Glyt2*-eGFP mice displayed an expression profile similar to adult mice, with a predominantly inhibitory glycinergic and/or GABAergic phenotype (Extended Data Fig. 8b,c).

### OT amplifies preBötC^OT-R^→nA^c^^ardiac^ glycinergic connectivity during inspiration

To assess the effect of TGOT application on preBötC^OT-R^→nA^c^^ardiac^ connectivity, we performed simultaneous extracellular recordings of preBötC population activity, and whole-cell current-clamp and voltage-clamp recordings of neurons within the nA boundaries that presented modulation of their activity in phase with the preBötC inspiratory bursts (Fig. 5j,k). Post hoc immunolabeling confirmed that the recorded neurons were nA neurons (Fig. 5m). As shown previously^12,13^, we found nA^c^^ardiac^ neurons with spiking activity during the expiratory phase of the respiratory cycle, and with hyperpolarizing synaptic currents that stopped firing during inspiratory bursts. We recorded from nA neurons that were all active during the expiratory period and hyperpolarized during preBötC inspiratory bursts (current-clamp), owing to large outward inhibitory currents (voltage-clamp, −40 mV holding voltage) (Fig. 5j,k). TGOT application amplified the volleys of inhibitory currents during inspiratory bursts in all recorded nA neurons (+53.8 ± 10.1%, *n* = 10 neurons, each from a different mouse) (Fig. 5k,l). Application of the GABA_A_ receptor antagonist bicuculline had no effect, while application of the glycine receptor antagonist strychnine abolished the inhibitory currents during inspiratory bursts in all nA neurons. Following strychnine application, half of these nA neurons showed an absence of remaining currents, while the other half showed excitatory currents during preBötC inspiratory bursts that were undetectable before strychnine application (blocked by the AMPA/kainate receptor antagonist CNQX; Extended Data Fig. 8d). Moreover, a post-inhibitory rebound was evident after each volley of inhibitory currents during inspiratory bursts on nA neurons (Fig. 5k), inducing an increase in spiking activity immediately after each inspiratory burst (Fig. 5j), which tended to be amplified by TGOT. Together, these electrophysiological data show that OT raises the excitability of inspiratory bursting preBötC^OT-R^ neurons, leading to the amplification of the preBötC^OT-R^→nA^c^^ardiac^ glycinergic connectivity during inspiratory activity.

### OT amplifies RespHRV through cardiac parasympathetic activity

**Fig. 6:**
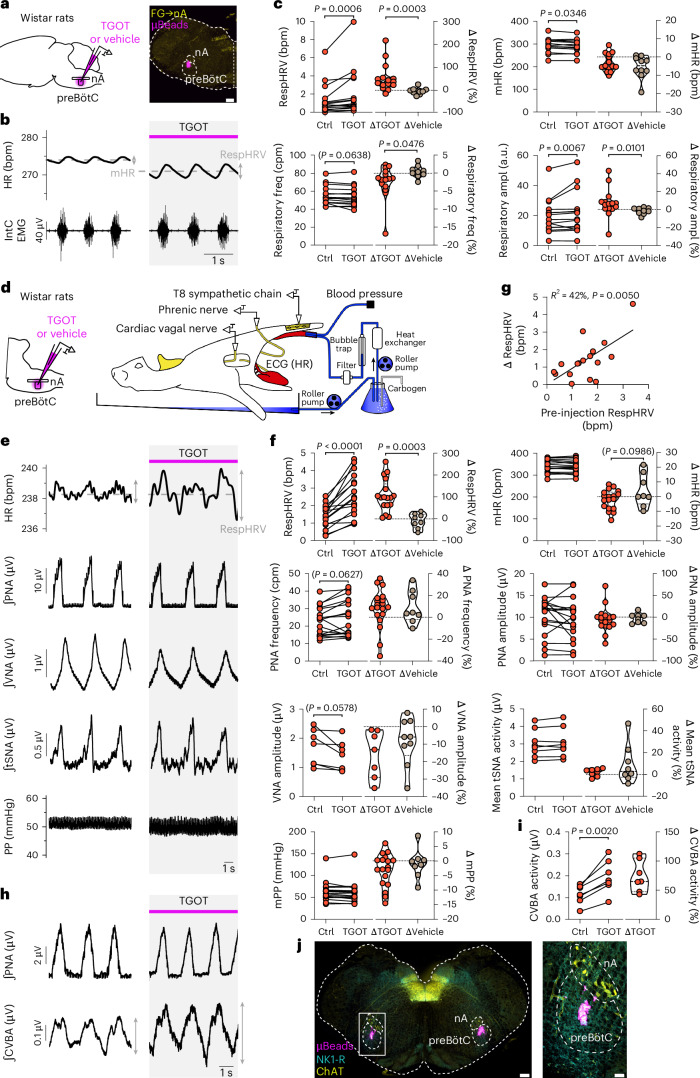
TGOT injection in the preBötC induces RespHRV amplification owing to amplified respiratory modulation of cardiac parasympathetic activity in adult anesthetized rats and in in situ WHBP of juvenile rats. **a**–**c**, The bilateral injection of TGOT (50 nl at 0.5 µM) in the preBötC of adult anesthetized Wistar rats (**a**) induced an amplification of RespHRV, a decrease in mHR and an increase in respiratory amplitude but no change in respiratory frequency (**b**,**c**). In violin plots, dashed lines indicate the median and dotted lines represent the quartiles (**c**). Mapping of injection spots (**a**) in Extended Data Fig. 9c (*n* = 15 TGOT and *n* = 10 vehicle). Scale bar, 300 µm (**a**). Before vs after TGOT injection (*n* = 15, **c**), paired two-sided *t*-test (respiratory frequency) or Wilcoxon two-sided matched-pairs signed rank test. Δ effects induced by TGOT or vehicle (*n* = 10, absolute values in Extended Data Fig. 9b) injections, Mann–Whitney two-sided test or unpaired two-sided *t*-test (mHR). **d**–**j**, Bilateral TGOT injection in the preBötC of in situ WHBPs (**d**) of juvenile Wistar rats (P21–P30) induced an amplification of RespHRV (**e**,**f**) and an increase in the respiratory modulation of the cardiac vagal branch activity (CVBA) (**h**,**i**), with no change in mHR, amplitude or frequency of the phrenic nerve activity (PNA), amplitude of the cervical vagal nerve activity (VNA), mean thoracic sympathetic nerve activity (tSNA) or mean perfusion pressure (PP). Before vs after TGOT injection (**f**,**i**) (RespHRV, *n* = 16; mHR, *n* = 18; PNA frequency, *n* = 19; PNA amplitude, *n* = 18; VNA, *n* = 7; tSNA, *n* = 8; mPP, *n* = 18; CVBA, *n* = 7), paired two-sided *t*-test or Wilcoxon two-sided matched-pairs signed rank test. Δ effects induced by TGOT or vehicle injections (absolute values for vehicle injections in Extended Data Fig. 9e; RespHRV, *n* = 8; mHR, *n* = 8; PNA frequency, *n* = 9; PNA amplitude, *n* = 8; VNA, *n* = 9; tSNA, *n* = 8; mPP, *n* = 8), Mann–Whitney two-sided test or unpaired two-sided *t*-test. In violin plots, dashed lines indicate the median and dotted lines represent the quartiles (**f**,**i**). Pearson two-sided correlation analysis, simple linear regression plotted (**g**). Mapping of injection spots (**j**) in Extended Data Fig. 9h (*n* = 19 TGOT and *n* = 4 vehicle). Scale bars: large image, 300 µm; inset, 100 µm. Detailed statistics are presented in Supplementary Table 1. Source data

**Extended Data Fig. 9:**
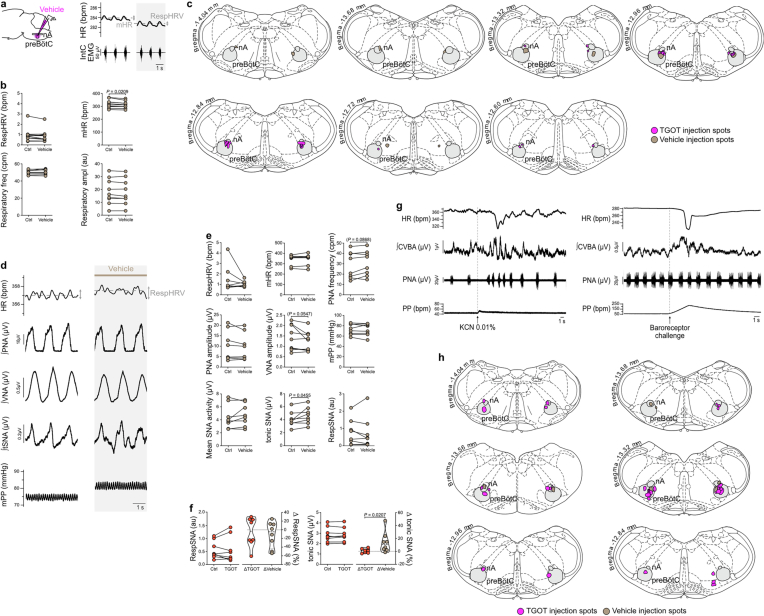
Vehicle injections in the preBötC of adult anesthetized rats and in situ Working Heart-Brainstem Preparations of juvenile rats, maps of preBötC injections, and functional characterization of CVBA recordings. **a-b**, Bilateral injection of vehicle in the preBötC of adult anesthetized Wistar rats had no effect on HR and inspiratory activity (IntC EMG). Absolute values are represented for the conditions before and after vehicle injection (*n* = 10). Paired two-sided *t*-test for respiratory frequency, respiratory amplitude and mHR, Wilcoxon two-sided matched-pairs signed rank test for RespHRV. **c**, Schematic coronal maps showing the distribution of all TGOT and vehicle injection spots in adult anesthetized Wistar rats. **d-e**, Effects of vehicle injection in the preBötC of in situ Working Heart-Brainstem Preparations (WHBP) of juvenile Wistar rats (P21-30) (HR, *n* = 8; PNA frequency, *n* = 9; PNA amplitude, *n* = 8; VNA, *n* = 9; tSNA, *n* = 8; mPP, *n* = 8). Absolute values are represented for the conditions before and after vehicle injection (**e**). Paired two-sided *t*-test or Wilcoxon two-sided matched-pairs signed rank test. **f**, Effects of bilateral TGOT vs. vehicle injection in the preBötC of in situ WHBP of juvenile Wistar rats on the two components of tSNA, the phasic burst of respiratory modulation of tSNA (RespSNA) and the tonic SNA (*n* = 8 TGOT and *n* = 8 vehicle). Before vs. after injection conditions, paired two-sidedt-test. Comparison of the relative effects of TGOT and vehicle (Δ in %), unpaired two-sided *t*-test for RespSNA, Mann-Whitney two-sided test for tonic SNA. **g**, Peripheral chemoreflex (potassium cyanide (KCN) injection) and baroreflex (increased perfusion flow rate) challenges were used to confirm functionally the validity of the CVBA recordings (data shown from the same preparation). **h**, Schematic coronal maps showing the distribution of all TGOT and vehicle injection spots in the in situ WHBP of juvenile Wistar rats. Detailed statistics are presented in Supplementary Table 1. Source data

So far, our data show that a PVN^OT^→preBötC^OT-R;glycine^→nA^c^^ardiac^ neuronal pathway can amplify RespHRV, with PVN^OT^ fibers releasing OT onto inhibitory preBötC^OT-R^ neurons to amplify the strength of the preBötC^OT-R^→nA^c^^ardiac^ glycinergic connectivity during the inspiratory phase of breathing. This finding strongly suggests that RespHRV is then amplified by enhanced modulation during inspiration of parasympathetic activity from nA^c^^ardiac^ neurons to the heart. However, preBötC neurons also provide modulation of pre-sympathetic neurons of the rostral ventrolateral medulla oblongata in phase with inspiration, producing a respiratory modulation of blood pressure^14,44,45^, which could indirectly regulate RespHRV amplitude. To address this possibility, we used the in situ Working Heart–Brainstem Preparation (WHBP), which enables recording autonomic and respiratory nerve activities in a perfused and decerebrate preparation devoid of the depressant effect of anesthesia^15^. We used rats because this preparation functions considerably better in rats than in mice, and because the difficult dissection and recording of the vagal branch that provides parasympathetic activity to the heart is more achievable in rats^46^. However, given that all our work so far was done in mice, we first used in vivo anesthetized rats to confirm the reproducibility of results between both species. Injection of TGOT bilaterally in the preBötC of anesthetized rats induced an amplification of RespHRV (+52.7 ± 15.8%), a very modest mHR decrease (−3.1 ± 1.3 bpm), no alteration in respiratory frequency and an increase in inspiratory bursts amplitude (+7.0 ± 4.1%) (Fig. 6a–c and Extended Data Fig. 9a–c). These effects are therefore very similar to those produced in anesthetized mice by OT release in the preBötC during optogenetic stimulation of PVN^OT^ fibers (Fig. 2). Then, using the in situ WHBP, injection of TGOT bilaterally in the preBötC induced an amplification of RespHRV (+105.3 ± 18.3%), no change in mHR and no alteration in the frequency and amplitude of inspiratory and post-inspiratory discharges recorded from the phrenic and cervical vagus nerves, respectively (Fig. 6d–f,j and Extended Data Fig. 9d–f,h). TGOT injection in the preBötC induced no change in thoracic (T8–T10) sympathetic chain recordings, which carry vasomotor activity, and no change in perfusion pressure, which in this preparation is the equivalent of blood pressure. Conversely, TGOT injection in the preBötC induced an amplification of the respiratory modulation of cardiac parasympathetic activity (+73.6 ± 10.1%), recorded from the left thoracic cardiac vagal branch^46^ (Fig. 6h,i and Extended Data Fig. 9g). These data show that OT in the preBötC induces RespHRV amplification by enhanced respiratory modulation of cardiac parasympathetic activity to the heart, which increases the disinhibition of the heart during inspiration, and not by modulation of sympathetic vasomotor activity.

### OT neurons participate in RespHRV recovery following stress

**Fig. 7:**
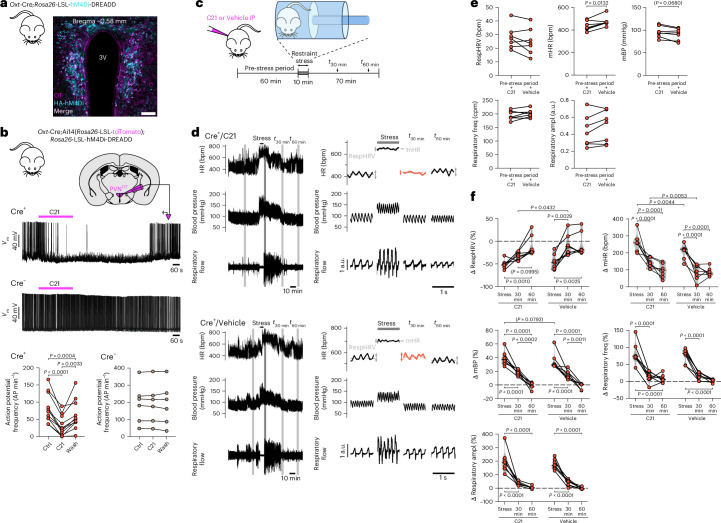
Chemogenetic inhibition of all OT neurons slows down the recovery of RespHRV amplification following a restraint stress test. **a**, Expression of the HA-tagged inhibitory chemogenetic actuator hM4Di in all OT neurons in adult transgenic mice (*n* = 4 mice). 3V, third ventricle. Scale bar, 100 µm. **b**, Whole-cell current-clamp recordings of PVN^OT^ neurons expressing tdTomato and hM4Di in slices from Cre^+^ mice, showing that application of the DREADD agonist C21 (10 µM, for 5 min) inhibits action potentials. C21 had no effect on neurons recorded in the PVN in Cre^−^ mice (*n* = 3 Cre^+^ and *n* = 2 Cre^−^ P20 mice, at least three recorded neurons per mouse, each from a different slice). Repeated-measures one-way ANOVA with the Geisser–Greenhouse correction, Tukey’s multiple comparison. **c**, Experimental strategy to test the effect of chemogenetic inhibition of OT neurons on respiratory and cardiovascular parameters, before and after a restraint stress test in a ventilated cylinder. The mice were implanted with blood pressure telemetry probes to enable measurements of mean blood pressure (mBP) and HR. They were placed in a plethysmography chamber (to which they were habituated for 1 week) during the pre-stress and post-stress conditions, to enable measurement of their respiratory activity. **d**–**f**, Effects induced by the intraperitoneal injection of the hM4Di agonist C21 or vehicle in adult Cre^+^ mice (*n* = 7, three trials averaged for each condition (C21 and vehicle) in each mouse; C21 or vehicle trials spaced by at least 24 h). Traces on the right for both the C21 and vehicle conditions are enlargements of the grayed areas on their respective traces on the left (**d**). Chemogenetic inhibition of OT neurons did not alter RespHRV amplitude at rest (**e**), but slowed down the recovery of RespHRV amplification following the restraint test (orange traces in **d**) (**f**). Pre-stress conditions, C21 vs vehicle; paired two-sided *t*-test (**e**). Stress and post-stress Δ (in % or bpm) effects compared to the pre-stress condition, C21 vs vehicle, repeated-measures two-way ANOVA with Šidák’s multiple comparison (**f**). Cre^−^ data are shown in Extended Data Fig. 10. Violin plots are represented in gray, dashed lines indicate the median and dotted lines represent the quartiles (**f**). Detailed statistics are presented in Supplementary Table 1. Source data

**Extended Data Fig. 10:**
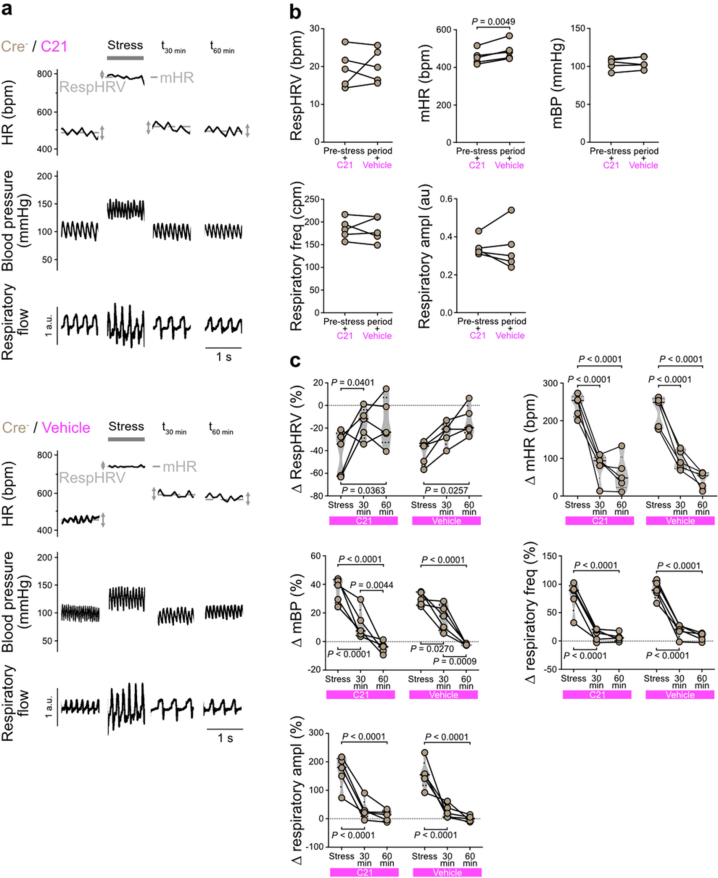
The DREADD agonist Compound 21 is mostly inert on cardiovascular and respiratory functions in OT::Cre;R26-LSL-hM4Di-DREADD Cre^−^ mice, except for mHR. **a-c**, Effects induced by the intraperitoneal injection of the hM4Di agonist Compound 21 (C21) or vehicle, before, during and after a restraint stress test in a ventilated cylinder, in Cre^−^ mice from the OT::Cre;R26-LSL-hM4Di-DREADD transgenic mouse line (*n* = 5, three trials averaged for each condition, C21 and vehicle, in each mouse; C21 or vehicle trials spaced by at least 24 h). There were no off-target effects of C21 in Cre^−^ mice on RespHRV amplitude, mBP, respiratory frequency and respiratory amplitude during the pre-stress condition (**b**), nor on the effect of stress and during the recovery from stress (**c**), but mHR was increased during the pre-stress condition (**b**). Pre-stress conditions, C21 vs. vehicle, paired two-sided *t*-test (**b**). Stress and post-stress Δ (in % or bpm) effects compared to the pre-stress condition, C21 vs. vehicle, repeated-measures two-way ANOVA, Sidak’s multiple comparison (**c**). Detailed statistics are presented in Supplementary Table 1. Source data

We have identified a multisynaptic neuronal pathway for OTergic amplification of RespHRV, from the hypothalamus to the brainstem to the heart. This pathway can be activated and induce RespHRV amplification during rest (Fig. 1e–g). Given that RespHRV amplitude is increased during relaxation and calming behaviors^2,7^, we tested whether OT neurons can be activated endogenously to participate in the recovery of RespHRV amplitude during recovery from stress. We used *Oxt*-Cre; *Rosa26*-LSL-hM4Di-DREADD male adult mice to chemogenetically inhibit all OT neurons in vivo in freely moving conditions (Fig. 7 and Extended Data Fig. 10). Cardiovascular and respiratory functions were assessed with blood pressure telemetry recordings and whole-body plethysmography. Each mouse was habituated to a dedicated plethysmography chamber for 1 week, with nesting material from their home cage. At the end of this habituation period, the mice exhibited a predominantly calm and resting behavior in their plethysmography chamber. Then, the mice were injected intraperitoneally with either the DREADD agonist Compound 21 (C21) or vehicle, followed by a resting phase in their plethysmography chamber, a restraint stress test phase in a ventilated cylinder and a recovery phase back in their plethysmography chamber (Fig. 7c). Compared to vehicle, C21 injections induced no change in RespHRV amplitude, blood pressure or respiratory frequency and amplitude during the pre-stress resting phase (Fig. 7d,e and Extended Data Fig. 10). This shows that OT neurons do not influence RespHRV amplitude in resting conditions. However, C21 injection decreased mHR compared to vehicle in both Cre^+^ (−39.3 ± 11.3 bpm) and Cre^−^ (−32.7 ± 5.8 bpm) mice, indicating an off-target effect of C21 on mHR. Although all DREADD agonists have been shown to exhibit off-target effects^47^, importantly, here, RespHRV amplitude and respiratory parameters were not affected.

Restraint stress induced a strong decrease (by ~50 %) in RespHRV amplitude of the same magnitude in all groups, and a strong increase in mHR, blood pressure and respiratory parameters (Fig. 7c,d,f and Extended Data Fig. 10). During the recovery from stress, chemogenetic inhibition of all OT neurons specifically delayed the recovery in RespHRV amplitude, but not the return to basal values of other cardiovascular and respiratory parameters. Indeed, in vehicle-injected Cre^+^ mice, and in all Cre^−^ mice, RespHRV amplitude, mHR and respiratory frequency and amplitude had returned to baseline values 30 min after the stress test. In C21-injected Cre^+^ mice, all parameters followed the same recovery kinetics except RespHRV amplitude, which returned to baseline value only 60 min after the stress test. We conclude that the neuronal pathway for OTergic amplification of RespHRV is used endogenously during recovery from stress, a calming behavior.

## Discussion

**Fig. 8:**
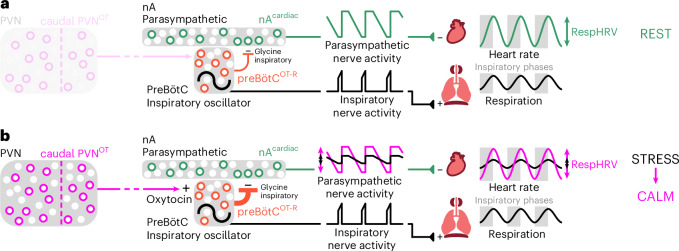
Schematic model of the preBötC^OT-R^→nA^c^^ardiac^ neuronal circuit that controls RespHRV at rest and its modulation by PVN^OT^ neurons for RespHRV amplification during a calming behavior. **a**, At rest, OT is not involved in the regulation of RespHRV amplitude. Yet expression of the OT-R in a subgroup of preBötC neurons, the group that generates the inspiratory rhythm, defines a subpopulation of cells that participates in the control of RespHRV. PreBötC^OT-R^ neurons are active during inspiration and induce glycinergic inhibition of the activity of nA^c^^ardiac^ neurons by putative monosynaptic contacts. This results in a decrease in cardiac parasympathetic activity that is concomitant with each inspiratory burst in the phrenic nerve and at the effector level in an increase in HR during inspiration. **b**, PVN^OT^ neurons are involved in the recovery of RespHRV amplitude during calming behavior following a stress. The release of OT by PVN^OT^ neurons raises the excitability of preBötC^OT-R^ neurons, leading to the amplification of the preBötC^OT-R^→nA^c^^ardiac^ glycinergic connectivity during inspiration. This induces an amplification of the respiratory modulation of parasympathetic activity to the heart, and thus an amplification of RespHRV. OT released endogenously in the preBötC induces little modulation of respiratory activity, which is not correlated with the RespHRV amplitude. Inhibition of PVN^OT^ neurons slows the recovery of RespHRV amplitude following a stress.

We have identified a multisynaptic neuronal pathway, from the hypothalamus to the brainstem to the heart, for the OTergic amplification of RespHRV, and its implication in the recovery from stress (Fig. 8). OT neurons in the caudal PVN in the hypothalamus project to the preBötC/nA and the DMV in the brainstem. PVN^OT^ fibers exert dual control on HR through both areas, namely by inducing RespHRV amplification through the release of OT in the preBötC/nA and by concomitantly decreasing mHR via the DMV. OT-Rs are expressed in a subgroup of preBötC neurons that are predominantly inhibitory (glycinergic and/or GABAergic), that are active during inspiration and that regulate both respiratory and cardiac activities; thus, preBötC^OT-R^ neurons form a cardiorespiratory hub that controls RespHRV amplitude. PreBötC^OT-R^ neurons make putative monosynaptic contacts with nA^c^^ardiac^ neurons; OT increases their excitability, amplifying the inhibitory glycinergic connectivity during inspiration from preBötC^OT-R^ to nA^c^^ardiac^ neurons. This process induces an amplification of the respiratory modulation of cardiac parasympathetic activity, resulting in an increased disinhibition of the heart during inspiration, thus amplifying RespHRV. By contrast, sympathetic vasomotor activity is not affected. Our work has therefore identified a PVN^OT^→preBötC^OT-R;glycine^→nA^c^^ardiac^→parasympathetic→heart pathway for RespHRV amplification, which was found in female and male mice as well as in rats. This connectivity was identified on different experimental preparations in newborn, juvenile and adult animals. Behaviorally, OT does not influence RespHRV amplitude in resting adult animals, a condition in which RespHRV amplitude is already large. A stressful event, such as a restraint test, induces a substantial decrease in RespHRV amplitude; OT neurons are involved in the rapid recovery of RespHRV amplitude following stress but not in the recovery of other respiratory and vascular parameters.

OT neurons were previously shown to regulate cardiac function, including by decreasing mHR, through an action on DMV^c^^ardiac^ neurons^25,29^. However, DMV^c^^ardiac^ neurons represent only 20% of all cardiac parasympathetic preganglionic neurons, and their axons are unmyelinated C fibers in rodents. Our work showing that OT neurons can only evoke a small (<5%) decrease in mHR is consistent with the bradycardia levels previously obtained upon direct stimulation of DMV neurons or regulation by OT^48,49^. Conversely, nA^c^^ardiac^ neurons represent the remaining 80% of all cardiac parasympathetic preganglionic neurons, and their axons are myelinated B fibers. Stimulation of nA neurons induces fast and profound bradycardia^32^, which is consistent with the encoding of RespHRV—that is, fast and large oscillations in HR—via nA^c^^ardiac^ neurons. We show that OT can induce a large amplification of RespHRV (>50%) by modulating the strength of the preBötC^OT-R;glycine^→nA^c^^ardiac^ neuronal connectivity. Moreover, we found that RespHRV amplitude and mHR are not correlated in the basal state and follow different dynamics when modulated by OT (Figs. 1h, 2d and 6g and Extended Data Fig. 6). This was observed across various experimental conditions, from the in vivo freely moving condition that enables conscious and behavioral modulations of cardiorespiratory activities, to the reduced in situ WHBP that isolates the brainstem cardiorespiratory neuronal networks in an experimentally controlled homeostatic state. Therefore, our data provide further evidence for the dichotomy in the regulation of cardiac activity by nA^c^^ardiac^ and DMV^c^^ardiac^ neurons, with OT neurons independently regulating RespHRV amplitude and mHR via each nucleus (respectively).

The present study identifies a phenotypic signature of key nodal neurons, namely preBötC^OT-R^ neurons, which control RespHRV amplitude by generating at least part of the inspiratory component of RespHRV. Optogenetic modulation of the activity of preBötC^OT-R^ neurons in vivo induced large and bidirectional variations in RespHRV amplitude (+80% to −50%), indicating that preBötC^OT-R^ neurons have a significant role in setting the ongoing RespHRV level (Fig. 4a–d and Extended Data Fig. 7h–k). PreBötC^OT-R^ neurons express a predominantly inhibitory, glycinergic and/or GABAergic phenotype (Fig. 3d–f and Extended Data Fig. 7d–g). All recorded preBötC^OT-R^ neurons exhibit phasic inspiratory bursting activity, with a subset also displaying tonic activity (Fig. 5f,g). PreBötC^OT-R^ neurons are pre-synaptic partners to nA^c^^ardiac^ neurons, as evidenced by viral tracing (Fig. 4e–g) and electrophysiology (Fig. 5). OT depolarizes preBötC^OT-R^ neurons, leading to an amplification of the glycinergic (but not GABAergic) post-synaptic currents recorded in nA neurons during inspiratory bursts (Fig. 5 and Extended Data Fig. 8d). Collectively, these data show that preBötC^OT-R^ neurons generate a monosynaptic, inhibitory modulation of nA^c^^ardiac^ neurons during inspiration, contributing to the generation of the inspiratory component of RespHRV, which is amplified by OT. This is a major component in the generation of RespHRV, given that HR automatically decreases after inspiration owing to the predominant parasympathetic tone in mammals at thermoneutrality. Still, part of the cardiac parasympathetic tone originates from respiratory neurons that are active during early expiration (post-inspiration), probably the pontine Kölliker–Fuse neurons^46^. Future studies could investigate a putative role of OT in the amplification of the expiratory component of RespHRV.

The effects induced by the photomodulation of preBötC^OT-R^ neurons highlight the important distinction between the role of these neurons in controlling ongoing RespHRV amplitude and their modulation exerted by OT. Tonic (50 Hz) photoexcitation of preBötC^OT-R^ neurons increased mHR and decreased RespHRV amplitude. This tonic photoexcitation would increase the glycinergic modulation of nA^c^^ardiac^ neurons by preBötC^OT-R^ neurons, decreasing cardiac parasympathetic activity and inducing the measured increase in mHR, while the pattern of modulation of nA^c^^ardiac^ neurons would be shifted from receiving a phasic synaptic inhibition in phase with inspiration to receiving a predominant high-frequency tonic inhibition, inducing the measured decrease in RespHRV amplitude. Although these findings underscore the pivotal function of preBötC^OT-R^ neurons in controlling the ongoing RespHRV amplitude, the tonic high-frequency photoexcitation strategy used here, induced by the large activation of the Chrimson-mediated cationic currents, displaces the intrinsic physiological activity of preBötC^OT-R^ neurons (that is, phasic discharge during inspiration) into an experimentally induced artificial state (that is, high-frequency tonic firing). Consequently, this strategy does not accurately reflect the endogenous and precise modulation of the activity of preBötC^OT-R^ neurons that would be achieved by OT through activation of the G-protein-coupled OT-R. In vitro pharmacology showed that OT can depolarize preBötC^OT-R^ neurons and amplify the preBötC^OT-R;glycine^→nA^c^^ardiac^ neuronal connectivity during inspiratory bursts (Fig. 5g–m). In vivo, the endogenous release of OT onto preBötC^OT-R^ neurons triggered by optogenetic stimulation of PVN^OT^ fibers induced a large amplification of RespHRV with very small changes in mHR (Figs. 1d–g and 2 and Extended Data Fig. 5a–d,f), indicating that preBötC^OT-R^ neurons function as an endogenous proxy to specifically control RespHRV amplitude. These effects of OT are strikingly similar to those identified in the hippocampus and amygdala, where OT also amplifies the gain of inhibitory synaptic connectivity^27,34,35^. The precise molecular mechanism by which OT amplifies the preBötC^OT-R;glycine^→nA^c^^ardiac^ neuronal connectivity during inspiration, leading to a specific amplification of RespHRV, remains to be elucidated, and is probably complex, given the large variety of intracellular pathways that the OT-R can activate, depending on the OT concentration released and the subcellular localization of the OT-R^50,51^.

The tonic photoinhibition of preBötC^OT-R^ neurons decreased mHR and increased RespHRV amplitude. Although the decreased mHR was anticipated, through mechanisms opposite those induced by the photoexcitation of preBötC^OT-R^ neurons and discussed above, the underlying mechanism responsible for the increased RespHRV amplitude remains unclear. It was thought that the tonic photoinhibition of preBötC^OT-R^ neurons would remove the phasic modulation during inspiration of the activity of nA^c^^ardiac^ neurons and decrease RespHRV amplitude. The present study does not provide a definitive answer to this unexpected result; however, one hypothesis is that the photoinhibition of preBötC^OT-R^ neurons might remove the inhibitory influence that these neurons might otherwise exert upon other cell groups that are potentially involved in regulating RespHRV amplitude. These may include neurons belonging to the respiratory central pattern generator (Kölliker–Fuse, post-inspiratory complex and Bötzinger complex neurons) and those involved in mediating the influence of respiratory-modulated afferents (pulmonary, baroreflex and veno-atrial)^1–3^. Additionally, we previously showed that using the same optogenetic tool (*Gt*ACR2), but to photoinhibit all preBötC neurons, resulted in only transient inhibition of the phasic discharge activity of preBötC neurons associated with inspiration^14,52^. These neurons rapidly escaped inhibition to restore the inspiratory phase of the breathing cycle, while the tonic preBötC neurons regulating cardiac activity probably remained inhibited. It is therefore possible that the phasic activity of preBötC^OT-R^ neurons during inspiration rapidly escaped the *Gt*ACR2-mediated photoinhibition in the present study, explaining why RespHRV amplitude was not decreased, whereas the tonic activity remained inhibited, explaining why mHR decreased.

When anxious, stressed or scared and feeling our heart pounding in our chest, taking deep breaths is a universal way to calm down. Indeed, slow and deep breaths strongly amplify RespHRV. Ancestral breathing techniques, such as yogic pranayama, and more recent ones like ‘boxed’ breathing or the ‘365’ breathing method, all rely on deep, slow breathing to amplify RespHRV. Even Buddhist mantras and Catholic rosary prayers amplify RespHRV by inducing slow, deep breathing^53^. Our work identifies a non-voluntary hypothalamus–brainstem neuronal pathway that similarly amplifies RespHRV when calming down during recovery from stress. This pathway could participate more globally in the amplification of RespHRV during positive socio-emotional states in humans when breathing is not consciously altered, such as during parental bonding. Interestingly, the OTergic amplification of RespHRV is mediated by a specific amplification of the respiratory→cardiac parasympathetic (preBötC^OT-R;glycine^→nA^c^^ardiac^) connectivity, with very little (if any) alteration in the respiratory activity per se. Therefore, RespHRV amplification by OT administration could be used as a therapeutic strategy in cardiovascular and mental disorders in which RespHRV amplitude is severely decreased, without adverse effects on respiratory function. Finally, whether variations in RespHRV amplitude are exclusively a physiological representation of mental states or can also cause variations in mental states per se remains unanswered. Recently, the finding that tachycardia is anxiogenic, which had been empirically accepted but never definitively proven, was demonstrated through elegant and selective optogenetic control of HR^54^. It is therefore probable that the OTergic amplification of RespHRV induces beneficial effects for both cardiac physiology and mental states, which are aspects that remain to be further investigated.

## Methods

### Animal experiments

Animals were handled and cared for in accordance with the Guide for the Care and Use of Laboratory Animals (N.R.C., 1996) and the European Communities Council Directive of 22 September 2010 (2010/63/EU,74). Experimental protocols were approved by the Institutional Ethical Committee no. 014 of the French Ministry of Higher Education, Research and Innovation (authorization APAFIS nos. 12006 and 44474). All animal experiments were conducted with animals group-housed under a 12 h light–dark cycle, at a constant temperature (22 ± 1 °C) and humidity (55 ± 10 %), with ad libitum access to food and water. Animals were fed a standard chow (SAFE R0325, SAFE Diets) with an approximate composition of 21.4% crude protein, 5.1% fat, 4.0% crude fiber and 52.0% nitrogen-free extract (energy content, ~14.2 MJ kg^−1^). All efforts were made to reduce animal suffering and minimize the number of experimental animals.

Male and female animals were used for all experiments (unless specified). Mice used in this study included wild-type C57BL/6J (Charles River), wild-type Swiss (Janvier Labs), *Oxt*-Cre knock-in (The Jackson Laboratory, stock 024234)^55^, Ai27(*Rosa26*-LSL-ChR2-tdTomato) (Ai27; The Jackson Laboratory, stock 012567)^56^, *Oxtr*-Cre knock-in (The Jackson Laboratory, stock 031303)^57^, Ai14(*Rosa26*-LSL-tdTomato) (Ai14; The Jackson Laboratory, stock 007914)^58^, *Gad67*-eGFP (generously provided by A. Baude)^59^, *Glyt2*-eGFP (Zeilhofer lab, ETH Zurich)^60^, *Oxt*-GFP (GENSAT project: Tg(Oxt-eGFP)LC317Gsat/Mmucd) and *Rosa26*-LSL-hM4Di-DREADD (The Jackson Laboratory, stock 026219)^61^. Wild-type Wistar rats were obtained from Janvier Labs.

### Viruses

AAV9-hSyn-DIO-somBIPOLES-mCerulean (RRID:Addgene_154951, Addgene, 154951; titer: 1.7 × 10¹³ genome copies per ml) was used for optogenetic excitation and inhibition of preBötC^OT-R^ neurons in *Oxtr*-Cre mice^36^.

AAV1-phSyn1(S)-FLEX-tdTomato-T2A-SypEGFP-WPRE (RRID:Addgene_51509, Addgene, 51509; titer: 1.8 × 10¹³ genome copies per ml) was used for tracing experiments from preBötC^OT-R^ neurons to nA^c^^ardiac^ neurons in *Oxtr*-Cre mice^62^.

### Viral and tracer microinjections in the preBötC

For tracing experiments and somBiPOLES virus injections in the brainstem, 3–4-month-old male and female mice were used: wild-type C57BL/6J (*n* = 8), *Oxtr*-Cre; Ai14 (*Rosa26*-LSL-tdTomato) (*n* = 4) and *Oxtr*-Cre (*n* = 18). Mice received pre-surgical injection of a non-steroidal, anti-inflammatory drug (carprofen (Rimadyl), 5 mg kg^−1^, subcutaneously) and were anesthetized with isoflurane (4% induction, 2% maintenance in 80% O_2_; Isorane, Axience). Body temperature was maintained at 37.5 °C with a heating pad (model 40-90-8D, FHC). Mice were placed in a stereotaxic frame.

For tracer injections, the head was tilted 40° ventro-flexed, a midline incision of the skin and muscle tissues was made at the occipital level, a cranial window was created on the caudal occipital bone using a dental drill and the atlanto-occipital membrane was opened to expose the brainstem. Glass micropipettes (~30 µm tip diameter) were filled with injectate and connected by a silver wire to an electrophysiology amplifier (Neuro Amp Ex, ADInstruments) for neuronal activity recording. The pipette (20° angled in the rostro-caudal axis, tip pointing forward) was descended into the brainstem 1.25 mm lateral to the midline. Extracellular recordings of multiunit activity were used to functionally map the preBötC, defined as the most rostral point displaying distinct inspiratory-locked activity (typically 0.0 ± 0.2 mm rostral, 1.4 ± 0.2 mm ventral to the calamus scriptorius).

For somBiPOLES virus injections, the head was kept horizontal, and the cranial window was made at the rostral end of the occipital bone. The extracellular recording pipette was tilted 10° in the rostro-caudal axis, pointing towards the caudal part of the mouse. Glass micropipettes were descended through the cerebellum to the brainstem, 1.25 mm lateral to the midline. Injection spots were typically 1.7 ± 0.3 mm caudal and 6.0 ± 0.3 mm ventral to lambda.

Bilateral injections were performed in all cases, except for unilateral FluoroGold experiments. Viruses and tracers were microinjected using a picospritzer (PICOSPRITZER III, Parker) and a graduated monocular. Injection details were as follows: viruses: 25 nl for the tracing virus and 35 nl for the somBiPOLES virus per injection site over 5 min into two rostro-caudal sites 200 µm apart; cholera toxin subunit B Alexa Fluor 488 Conjugate (CTB-488, 0.01% in 0.9% NaCl; ThermoFisher Scientific, C34775): 70 nl per injection site over 5 min into three rostro-caudal sites 200 µm apart; FluoroGold (0.5 % in 0.9% NaCl; Fluorochrome): 25 nl per injection site over 5 min into two rostro-caudal sites 200 µm apart.

At each site, pipettes were left in place for 10 min post injection before withdrawal. Wounds were closed using sterile sutures. Experiments were conducted 3 weeks after viral injections and 1 week after tracer injections. Injection sites were verified post hoc.

### Optogenetic cannula and ECG telemetric transmitter implantation

For in vivo freely moving experiments, *Oxt*-Cre; Ai27(*Rosa26*-LSL-ChR2-tdTomato) female mice (3–4 months old, *n* = 8 Cre^+^, *n* = 4 Cre^−^) were anesthetized and placed in a stereotaxic frame. The surgical approach was the same as that used for the somBiPOLES virus injections described above. ECG and EMG inspiratory recordings were obtained using three electrodes: two in cranial muscles and one subcutaneously through a dorsal incision, which was subsequently used to insert the telemetric transmitter. Signals were amplified, filtered, digitized (Power 1401, Cambridge Electrical Design), rectified and integrated by Spike2 software (v.10; Cambridge Electrical Design). Extracellular recordings were performed to map the preBötC, using the same techniques as for somBiPOLES viral injections (typically 1.25 mm lateral to the midline and 1.2 ± 0.3 mm caudal, 6.0 ± 0.3 mm ventral to lambda). Bilateral optogenetic cannulas (200 µm, NA 0.22, Doric Lenses) were implanted at the preBötC rostro-caudal and medio-lateral coordinates. The dorso-ventral position was optimized based on maximal RespHRV amplification and bradycardia during optogenetic stimulation, typically 0.4 mm above the best extracellular inspiratory recording. Cannulas were fixed with cyanoacrylate (Super Glue, Loctite).

During the same surgery, ECG telemetry transmitters (ETA-F10, Data Sciences International (DSI), Harvard Bioscience) were implanted subcutaneously along the mouse’s flank, using the dorsal incision made at the beginning of the surgery. Two subcutaneous electrodes, previously exposed by 1 cm, were sutured near the right shoulder and the left rib region. The wound was closed with sterile sutures. The ECG signal was recorded (Matrix 2.0, DSI, Harvard Bioscience), sampled at 2,000 Hz and integrated into Spike2 software using a DSI talker.

After a 1-week recovery, respiratory activity was recorded by plethysmography and ECG by telemetry for 3 h, during which optogenetic stimulations were performed periodically.

### In vivo plethysmography recordings

Breathing of unrestrained, awake mice was recorded using whole-body plethysmography (EMKA Technologies). A constant air flow (0.5 l min^−1^) circulated through 200 ml plethysmography chambers (maintained at 25 ± 0.5 °C) using a vacuum pump (Vent 4, EMKA). During each recording, calibration was performed by injecting 1 ml air into the chamber with the mouse. Analog signals were amplified (Amplipower, EMKA), digitized (Power 1401, Cambridge Electrical Design) and integrated into Spike2 software (v.10, Cambridge Electrical Design).

### In vivo RespHRV recordings in anesthetized animals

Animals were anesthetized and placed in a stereotaxic frame with the nose ventro-flexed (40°), providing access to the brainstem surface. For mouse experiments, ECG and EMG inspiratory recordings were obtained using three electrodes placed under the skin of the hind legs and a front leg. For rat experiments, the EMG inspiratory recordings were obtained with external intercostal muscle electrodes, and the ECG was recorded using three electrodes placed under the skin of the hind legs and thorax. Signals were amplified, filtered, digitized (Power 1401, Cambridge Electrical Design), rectified and integrated by the Spike2 software (v.10, Cambridge Electrical Design).

In Wistar rats (2–3 months old, male and female, *n* = 25), as described above, extracellular recordings were made to functionally map the preBötC bilaterally (typically 1.8 mm lateral to the midline and 0.7 ± 0.2 mm rostral, 1.7 ± 0.3 mm ventral to the calamus scriptorius). A specific OT-R agonist (TGOT^39^ (4013837.0005, Bachem), 0.5 µM diluted in artificial cerebrospinal fluid (aCSF (composition as used for the WHBP below), 50 nl) or aCSF (vehicle injections, 50 nl) diluted with 0.5% fluorescent beads, was slowly injected bilaterally into each preBötC using a syringe and a graduated monocular.

In *Oxt*-cre; Ai27(*Rosa26*-LSL-ChR2-tdTomato) mice (3–4 months old, *n* = 15 Cre^+^ females, *n* = 15 Cre^+^ males, *n* = 5 Cre^−^ females and males), unilateral photostimulations were performed in the preBötC. The optical fiber was tilted by 20° in the rostro-caudal axis, with the tip pointing forward. The preBötC was located by the following coordinates: 1.25 mm lateral to the midline and 0.0 mm rostral to the calamus scriptorius. The dorso-ventral coordinate was functionally determined based on the optogenetically induced bradycardia and RespHRV amplification. After identifying the optimal position, a minimum of three stimulations were performed at intervals of at least 5 min. To precisely perform OT-R antagonist injections into the preBötC, extracellular recordings were made, as detailed above. Optimal inspiratory extracellular activities were generally located 400 µm ventral to the coordinate defined for the optogenetic stimulations. OT-R antagonist (d(CH_2_)₅¹,Tyr(Me)²,Thr⁴,Orn⁸,des-Gly-NH_2_⁹)-Vasotocin trifluoroacetate salt (OTA; Bachem, 4031339), 1 µM diluted in aCSF, 200 nl) was then injected unilaterally in the preBötC^25,27,63^. Several photostimulations were performed every 5 min for 1 h after OTA injection. In some animals, photostimulations were also performed on the contralateral preBötC and in the ipsilateral DMV (coordinates: 0.3 mm lateral to midline, 0.0 ± 0.3 mm rostral and 0.2 ± 0.2 mm ventral to the calamus scriptorius). For control experiments, aCSF without OTA was injected. Fluorescent beads (0.5%) were added to all injectate solutions to enable the localization of the injection spots a posteriori.

In mice injected with the somBiPOLES virus, bilateral photoexcitations and photoinhibitions were performed in the preBötC using the techniques described for photostimulations in *Oxt*-Cre; Ai27(*Rosa26*-LSL-ChR2-tdTomato) mice experiments. At the end of the experiment, intraperitoneal injection of atropine was administered (10 mg kg^−1^, diluted in 600 µl NaCl for a 30 g mouse; Sigma-Aldrich, A0132), followed by additional stimulations after a 20-min interval.

### Optogenetic stimulations

Unilateral or bilateral cannulas (200 µm, NA 0.22; Doric Lenses) were connected to optical fibers (200 µm, NA 0.22; Doric Lenses), themselves connected to a rotary joint, allowing beam splitting when needed. A common optical fiber was connected to either a blue laser (473 nm, DPSS, ADR-800A, Shanghai Laser & Optics) or a dual-wavelength laser (473 nm and 561 nm, DPSS, 06 Series, Cobolt AB, Hübner Photonics) for somBiPOLES experiments. Lasers were TTL-triggered using a PulserPlus generator (Prizmatix) controlled by Pulser software (v.3.2.1; Prizmatix), with signal integration via Spike2.

In *Oxt*-Cre; Ai27(*Rosa26*-LSL-ChR2-tdTomato) mice (freely moving or anesthetized), photostimulation was conducted using 10 ms pulses at 10 mW mm^−2^, 3,000 pulses at 50 Hz. Photostimulations in freely moving experiments were performed during calm periods characterized by a stable respiratory frequency.

In somBiPOLES-injected mice, photoinhibition (473 nm) used 10 ms pulses at 1.2 mW mm^−2^, 3,000 pulses at 50 Hz, and photoexcitation (561 nm) used the same protocol at 10 mW mm^−2^. The power difference reflects opsin sensitivity differences.

Photostimulations were separated by at least 5 min. Light intensity was calibrated with a power meter (PM100D, Thorlabs) up to 10 mW mm^−2^ (~400 µm from target) to ensure efficacy without tissue damage^64^. Each protocol was repeated three times or more per animal, with consistent intra-animal reproducibility.

### WHBP

The arterially perfused in situ WHBP^15,65^ was applied to juvenile Wistar rats of both sexes (P21–P30, *n* = 28). Rats were deeply anesthetized with isoflurane, bisected below the diaphragm, exsanguinated and cooled in carbogenated aCSF on ice (125 mM NaCl, 24 mM NaHCO_3_, 3 mM KCl, 2.5 mM CaCl_2_, 1.25 mM MgSO_4_, 1.25 mM KH_2_PO_4_ and 10 mM D-Glucose, pH 7.3; Sigma-Aldrich). After precollicular decerebration, the lungs were removed, and the descending aorta was isolated and cleaned. Preparations were transferred to a recording chamber. Retrograde perfusion of the thorax and head was achieved by a double-lumen catheter (ø1.27 mm, PowerPICC, Bard) inserted into the descending aorta. The perfusion solution consisted of aCSF containing Ficoll (1.25%; 17-0310-50, Sigma-Aldrich), vasopressin acetate (2 nM; V9879, Sigma-Aldrich) and vecuronium bromide (2–4 µg ml^−1^; PHR1627, Sigma-Aldrich), warmed to 31 °C and gassed with carbogen (closed loop reperfusion circuit, 25–30 ml min^−1^). Perfusion pressure was monitored with a transducer connected to the second lumen of the catheter. The head of the preparation was fixed with ear and mouth bars. A peristaltic pump (520S, Watson-Marlow Bredel Pumps) was used to slowly increase the flow rate of the perfusion solution to tune the preparation. For most WHBPs, simultaneous recordings of phrenic nerve activity, cervical vagus nerve activity (left side) and thoracic sympathetic chain activity (between T8 and T10) were obtained using glass suction electrodes.

For some WHBPs, the cardiac vagal branch activity was recorded instead of the cervical vagus nerve activity^46^. To expose the branch, a small amount of parenchymal tissue around the cut left bronchus was kept, and the thymus was resected to gain access to the left cardiac vagal branch. In the recording chamber, the left thoracic vagus nerve could be observed parallel to the esophagus and was carefully dissected away from the surrounding tissue to expose its branches. The left cardiac vagus branch was usually found just caudal to the left recurrent laryngeal nerve and was cut as distally as possible. Functional validation was done by transient baroreceptor stimulation (maximum perfusion for 2–3 s) and chemoreceptor activation (KCN injection, 0.2 ml, 0.01%; 1064371000, Sigma-Aldrich). Preparations failing these tests were either repositioned until successful or excluded.

All neurograms were amplified, filtered (300–5,000 Hz band-pass, A-M system, Model 1700), digitized (Power 1401, Cambridge Electrical Design), rectified and integrated using Spike2 software (v.10, Cambridge Electrical Design). HR was extracted from systolic perfusion pressure with a window discriminator.

Once tuned, bilateral brainstem extracellular recordings were performed to locate inspiratory activity from preBötC neurons. They were typically found 1.5 mm lateral, 0.9 ± 0.3 mm rostral and 2.0 ± 0.3 mm ventral to the calamus scriptorius. TGOT (0.5 µM, 50 nl, diluted in aCSF with 0.5% fluorescent beads) or aCSF (vehicle injections, 50 nl, with 0.5% fluorescent beads) were slowly injected in each preBötC bilaterally using a syringe and a graduated monocular.

### Rhythmic preBötC/nA slice and island preparations

Brainstem transverse slice preparations containing the preBötC network and the nA were obtained from mouse pups of both sexes (P0–P5) as follows. Swiss (*n* = 27) or *Oxtr*-Cre; Ai14(*Rosa26*-LSL-tdTomato) (*n* = 7) newborns were quickly decapitated at the C4 spinal level, and the brainstem was carefully dissected in a chilled oxygenated aCSF composed of 120 mM NaCl, 8 mM KCl, 1.26 mM CaCl_2_, 1.5 mM MgCl_2_, 21 mM NaHCO_3_, 0.58 mM NaH_2_PO_4_, 30 mM glucose, pH 7.4. The isolated hindbrain was embedded in a low-melting-point agar block (ThermoFisher) and oriented to enable serial transverse sectioning along the rostro-caudal axis using a vibratome (VT 1200S, Leica). A 500 µm thick slice, with its anterior limit set ~300 µm caudal to the posterior limit of the facial nucleus, was isolated. Additional anatomical landmarks, including the inferior olive shape and the fourth ventricle opening, guided rostro-caudal level selection^66^. Slices were then transferred to a recording chamber, rostral surface upwards, and continuously superfused with oxygenated aCSF at 30 °C. Preparations were allowed to recover for 20–30 min before any recording sessions began.

Island preparations were obtained from transverse slices by additionally trimming away tissue outside the region hosting the preBötC network and the nA using fine scissors^67^.

### Procedure for recordings in preBötC/nA slice and island preparations

PreBötC network activities in slice and island preparations were recorded using glass micropipettes (tip diameter, 80–100 µm) positioned on the slice’s rostral surface, ventral to the nA, where the preBötC respiratory circuitry is located. The micropipettes were fabricated from aCSF-filled borosilicate glass tubes (Harvard Apparatus) broken at the tip and used as suction electrodes connected to a high-gain amplifier (AM Systems). Signals were filtered (3 Hz–3 kHz), integrated (time constant, 100 ms; Neurolog, Digitimer) and digitized through a Digidata 1440 interface and pCLAMP v.10 software (Molecular Devices).

Whole-cell patch-clamp recordings were visually guided using differential interference contrast. For nA neurons recordings, the patch pipette was positioned close to the nA pars compacta, slightly dorso-lateral to the micropipette monitoring preBötC activity. The patch pipette for recording individual preBötC neurons was also positioned close to the extracellular pipette, and the recorded neurons were selected according to their activity in phase with the preBötC network activity. Patch pipettes were fabricated with borosilicate glass tubes using a puller (Sutter Instrument) and filled with a solution composed of 140 mM K-gluconate acid, 1 mM CaCl_2_·6H_2_O, 10 mM EGTA, 2 mM MgCl_2_, 4 mM Na_2_ATP, 10 mM HEPES pH 7.2, supplemented with Alexa Fluor 568 (Molecular Probes, ThermoFisher Scientific). Tip resistance was 5–7 MΩ when filled. Electrophysiological signals were recorded using an Axoclamp 2A amplifier (Molecular Devices) and the same digitizing interface and software mentioned before. nA neurons were selected based on their location (between the dorsal part of the preBötC and the ventral part of the nA pars compacta) and their rhythmic inhibitory synaptic inputs received during each preBötC respiratory burst. Synaptic drive was quantified as the maximal envelope amplitude of inhibitory currents during inspiratory bursts, averaged over ten consecutive bursts per neuron.

Drugs were applied in the bath at a final concentration of 0.5 µM TGOT (Bachem); 10 µM bicuculline (Trocris, Bio-Techne); 5 µM strychnine (Sigma-Aldrich); 20 µM CNQX (Sigma-Aldrich). One drug (or combination of drugs) application per slice.

### PVN slice preparations

P20 *Oxt*-Cre; Ai14(*Rosa26*-LSL-tdTomato); *Rosa26*-LSL-hM4Di-DREADD mice of both sexes (*n* = 3 Cre^+^, *n* = 2 Cre^−^) were anesthetized using 5% isoflurane and rapidly decapitated. Coronal slices containing the PVN (350 µm) were obtained using a vibratome (VT 1200S, Leica). Slices were allowed to equilibrate before use for 1 h in standard recording aCSF (124 mM NaCl, 3 mM KCl, 1.2 mM NaH_2_PO_4_, 1.2 mM MgSO_4_, 25 mM NaHCO_3_, 11 mM D-glucose and 2 mM CaCl_2_, saturated with 95% O_2_ and 5% CO_2_, pH 7.4, ~300 mosM) at room temperature (~22°C). During the experiments, the recording chamber was superfused at ~3 ml min^−1^ with standard recording aCSF.

### Procedure for recordings in PVN slices

Electrodes (resistance, ~3.0–6.0 MΩ) were prepared using a Narishige micropipette puller (PC-100) and filled with 130 mM K^+^-gluconate, 10 mM NaCl, 11 mM EGTA, 1 mM CaCl_2_, 10 mM HEPES, 1 mM MgCl_2_, 2 mM Mg-ATP, 0.2 mM Na-GTP, pH 7.3, ~280 mosM. Recording pipettes were guided using a piezoelectric manipulator (Luigs & Neumann). PVN^OT^ neurons expressing tdTomato were identified in Cre^+^ mice using a Hamamatsu scientific camera. In Cre^−^ mice, unlabeled neurons in caudal PVN slices were recorded. Resting membrane potential, spontaneous action potentials and evoked action potentials were recorded in current-clamp mode. The DREADD agonist C21 (10 µM) was applied for 5 min. Data obtained from PVN slice experiments were acquired in pCLAMP v.10.7 using a HEKA EPC 10 Double Amplifier (HEKA Elektronik), filtered at 2 kHz and sampled at 20 kHz. Data were analyzed using Clampfit (v.10.7, Molecular Devices) and MiniAnalysis (v.6.0.3, Synaptosoft).

### Histology and image acquisition

After in vivo anesthetized and WHBP experiments, brains were fixed in Antigenfix (Diapath, P0016) at 4 °C for 48–72 h and cryoprotected in 30% sucrose. After in vitro experiments, slices were fixed in 4 % paraformaldehyde for 2–3 h and cryoprotected in 20% sucrose. For other conditions, animals were transcardially perfused with 20 ml PBS, followed by 25 ml Antigenfix. Brains were post-fixed in Antigenfix at 4 °C for 24 h and cryoprotected in 30% sucrose. In experiments requiring nA labeling, animals received an intraperitoneal injection of the retrograde tracer FluoroGold (0.2%, 300 µl, Fluorochrome) 5 days before perfusion. Coronal brainstem or hypothalamus sections were cut using a cryostat (CM350S, Leica Microsystems): 30 µm for in vitro slices, 50 µm for others.

For immunohistochemistry, 50 µm sections were blocked and permeabilized in PBS (1×) containing 5% normal goat serum, 2% BSA and 0.3% Triton X-100 for 1 h at room temperature. Then, 30 µm sections were incubated in PBS solution with 1% BSA and 0.3% Triton X-100 for 90 min. Sections were incubated with primary antibodies for 72 h at 4 °C (50 µm sections) or overnight at room temperature (30 µm sections). After washing, sections were left for 2 h (50 µm sections) or 90 min (30 µm sections) with secondary antibodies at room temperature. Sections were mounted with Vectashield (H-1000 without DAPI or H-1200 with DAPI; VectorLabs). Primary antibodies used in this study included rabbit anti-CGRP (Sigma-Aldrich, C8198; 1:2,500), rabbit anti-NK1-R (Sigma-Aldrich, S8305; 1:5,000), mouse anti-Tyrosine Hydroxylase (Merck Millipore, MAB318; 1:1,000), goat anti-choline acetyltransferase (Merck Millipore, AB144P; 1:200), rabbit anti-glutamine synthetase (Sigma-Aldrich, G2781; 1:1,000), mouse anti-PS38 (a gift from H. Gainer; 1:50), chicken anti-GFP (Avès, 1020; 1:500), rabbit anti-DsRed (Clontech, 632496; 1:1,000), goat anti-mCherry (Sicgen antibodies, AB0040-200; 1:1,000), mouse anti-NeuN (Synaptic System, 266011; 1:500), rabbit anti-CTB (Invitrogen, PA1-25635; 1:1,000), rabbit anti-µOpioid Receptor (Abcam, Ab134054; 1:100), goat anti-BCHE (Bio-Techne, AF9024; 1:100), rabbit anti-calbindin (Swant, CB-38a; 1:8,000), rabbit anti-oxytocin receptor (Alomone labs, AVR-013; 1:200), mouse anti-HA tag (Cell Signaling Technology, 2367S; 1:300) and mouse anti-Phox2b (Santa Cruz, sc-376997; 1:100). Species-specific donkey secondary antibodies conjugated to Alexa Fluor 488, 555 or 647 were obtained from Invitrogen or Jackson ImmunoResearch and used at a 1:500 dilution.

Fluorescent staining was examined under a Zeiss LSM-800 (or LSM-900) confocal microscope with ×10, ×20 or ×40 objectives and captured using ZEN v.2.6 (blue edition) imaging software. For cell counting analysis, *z*-stack images with 0.5 µm *z*-steps were used. Images for figures are maximum intensity projections of 6–15 image stacks, produced using the ImageJ software (v.2.14.0/1.54f, NIH), with optimized low and high thresholds for gray levels using Photoshop (v.12.1, CS5, Adobe). For merged images, brightness and contrast were uniformly adjusted for each channel across the entire image using ImageJ (v.2.14.0/1.54f, NIH).

### RNAscope in situ hybridization and image acquisition

RNAscope in situ hybridization was performed using the RNAscope Multiplex Fluorescent Reagent Kit v.2 (Advanced Cell Diagnostics). The corresponding protocols provided by the manufacturer were adapted based on the literature and recommendations from the manufacturer. C57BL/6J wild-type mice of both sexes (*n* = 4; 2 months old) were transcardially perfused with PBS buffer (50 ml) followed by Antigenfix (50 ml). Brainstems were collected, post-fixed in Antigenfix for 24 h at 4 °C and cryoprotected in 30% sucrose for 3 days at 4 °C. Tissues were frozen, and 30 µm coronal sections were made using a cryostat (CM350S, Leica Microsystems). The sections were kept overnight at 4 °C in TBST buffer (1× Tris buffer saline with 0.1% Tween 20 detergent). Free-floating sections were incubated in RNAscope Hydrogen Peroxide for 20 min and rinsed five times in distilled water. Sections were mounted onto SuperFrost Plus Slides (ThermoFisher Scientific) and air-dried before being post-fixed for 15 min in Antigenfix at 4 °C. Following three PBS washes, tissues were dehydrated in successive 50%, 70% and 100% ethanol and baked at 60 °C for 30 min (HybEZ II Oven, Advanced Cell Diagnostics). Tissues were rehydrated in water and then air-dried for 1 h. Following target retrieval, RNAscope protease plus was applied for 30 min at 40 °C, and sections were rinsed in distilled water. Probes targeting mRNAs for OT-R (Mm-Oxtr-C1), and mRNAs encoding a glycinergic phenotype (Mm-Slc6a5-C3) and a glutamatergic phenotype (Mm-Slc17a6-C4), were applied for 2 h at 40 °C and then rinsed with fresh RNAscope wash buffer. Sections were stored overnight in 5× saline sodium citrate and rinsed in wash buffer in the morning. RNAscope Multiplex Fluorescent V2 Amp1 and Amp2 were added for 30 min at 40 °C, and Amp3 was added for 15 min at 40 °C. RNAscope Multiplex Fluorescent V2 HRP-C1 followed by Opal 690 (Akoya Biosciences) was used to identify OT-R mRNAs, HRP-C3 followed by Opal 520 was used to identify mRNAs encoding a glycinergic phenotype and HRP-C4 followed by Opal 570 was used to identify mRNAs encoding a glutamatergic phenotype; each followed by HRP-blocker incubation (15 min at 40 °C).

Fluorescent staining was examined under a Zeiss LSM-800 confocal microscope with a ×40 objective and captured using ZEN v.3.7 (blue edition) imaging software. Cell counting analysis and figure production followed the protocol described in the ‘Histology and image acquisition’ section.

### Mapping of injection spots

For all experiments with chemical compound injections in the preBötC, fluorescent beads (excitation, 580 nm; emission, 605 nm; 0.5% in aCSF; F8793, ThermoFisher Scientific) were co-injected to enable the localization of the injection spots. Sections containing fluorescent beads were co-labeled with TH and NK1-R antibodies to localize preBötC, as well as ChAT antibody or FluoroGold (0.2%, intraperitoneal, Fluorochrome) to visualize nA. Sections were then manually aligned to the Paxinos–Watson rat brain atlas^68^ or the Paxinos–Franklin mouse brain atlas^69^. Injection spots were imaged using an Apotome Zeiss epi-fluorescence microscope with a ×10 air objective and captured with an axioCam MRm camera and ZEN v.2.6 imaging software (blue edition).

### Tissue clearing

Paraformaldehyde-fixed whole *Oxt*-GFP mouse brains (*n* = 3, both sexes) were cut with a slicer to obtain coronal slices of ~1 mm thickness. Tissue was cleared using the CUBIC method^70–72^. Fixed tissue was immersed in 50%-diluted reagent-1A (containing 5% aminoalcohol #10, 10% Triton and 10% urea) for 6 h at 25 °C with shaking, and then replaced by reagent-1A for 42 h at 25 °C with shaking. From day 2 to day 4, tissue was shaken at 37 °C. Clearing was stopped with PBS. Slices were immersed in 50%-diluted reagent-2 (containing 50% sucrose, 25% urea, 10% aminoalcohol #16 and 0.1% Triton) for 24 h at 25 °C and then in reagent-2 for 48 h at 25 °C. Slices were mounted with a coverslip immersed in a 1:1 mixture of silicon oil and mineral oil. Images were acquired using a Zeiss LSM-800 confocal microscope.

### Blood pressure telemetric transmitter implantation

Male *Oxt*-Cre; *Rosa26*-LSL-hM4Di-DREADD mice (3–4 months old; *n* = 7 Cre^+^, *n* = 5 Cre^−^) were implanted with a telemetric blood pressure sensor (PA-C10, DSI, Harvard Bioscience) in the left carotid artery. Mice received a non-steroidal anti-inflammatory drug injection (carprofen (Rimadyl), 5 mg kg^−1^, subcutaneously) 30 min before surgery. Anesthesia was induced with isoflurane inhalation in an induction box, followed by an intraperitoneal injection of a ketamine–xylazine mixture (ketamine, 100 mg kg^−1^, Imalgene 1000; xylazine, 10 mg kg^−1^, Rompun 2%). Mice were positioned on their backs on a heating pad, maintaining their temperature at 37.5 °C. A midline neck incision was made, and the carotid artery sheath was accessed by gently displacing the salivary glands and muscles. The left carotid artery was isolated from the vagus nerve and carotid vein until the carotid bifurcation was located. A ligature was placed around the carotid bifurcation and used to create tension, facilitating the positioning of a vascular clamp approximately 1 cm upstream of the bifurcation. A needle was used to make an incision in the carotid artery, allowing catheterization with the telemetric sensor catheter. Once in place, the clamp was removed, and the catheter was inserted approximately 1 mm into the aorta and ligated to the carotid artery. The transmitter was subcutaneously inserted into the back of the animal. The incision was closed using sterile sutures, and lidocaine (5% cream, Lidocaïne Prilocaïne, Biogaran) was applied to the incision area. The animal was allowed to recover for 1 week.

### Behavioral studies of recovery from stress

Male *Oxt*-Cre; *Rosa26*-LSL-hM4Di-DREADD mice were habituated daily to the plethysmography chamber for 1 week before chemogenetic experiments. On the day of the experiment, mice received an intraperitoneal injection of C21 (2.5 mg kg^−1^ diluted in 0.9% NaCl, DREADD agonist 21 dihydrochloride; HB6124, Hello Bio) or vehicle solution (0.9% NaCl) under light isoflurane anesthesia. Mice were then placed in their plethysmography chamber, which was positioned on the receiver plate for simultaneous respiratory and blood pressure recording. Respiratory signals were amplified (Amplipower, EMKA) and digitized (Power 1401, Cambridge Electrical Design). Blood pressure telemetry signals were recorded (Matrix 2.0, DSI, Harvard Bioscience) and rectified according to the measurement of ambient pressure (APR-2, DSI, Harvard Bioscience). Respiratory and telemetric signals were integrated into Spike2 software (v.10, Cambridge Electrical Design) at a 2,000 Hz sampling rate. After a 1 h control period in a low-light room at room temperature, mice were placed in a restraining ventilated cylinder that severely restricted their movements for 10 min before returning to the plethysmography chamber for 70 min. Heart rate was derived from systolic blood pressure through automatic detection threshold with manual verification. Analysis was performed before the stress stimulus (during calm periods), during the stress phase and twice during the post-stress recovery phase (25–35 min and 55–65 min post stress). Stress effects on respiratory parameters and RespHRV were quantified within the first 5 min after mice returned to their plethysmography chamber. Each animal had six recording sessions over consecutive days, each lasting about 2.5 h, to obtain three recordings in each condition (C21 or NaCl). NaCl sessions always preceded C21 sessions owing to the compound’s prolonged effects (up to 6 h).

### Data analysis and statistics

Cell counting was performed manually and unilaterally on serial 1:4 sections, unless specified below. Cell counting of OT-R^+^, calbindin^+^, BCHE^+^ neurons in the preBötC/nA, as well as dual-label quantifications in *Oxtr*-Cre; Ai14(*Rosa26*-LSL-tdTomato); *Gad67*-GFP mice and *Oxtr*-Cre; Ai14(*Rosa26*-LSL-tdTomato); *Glyt2*-GFP mice were performed on serial 1:2 sections. PVN OT/ChR2 counts used three sections covering the rostro-caudal axis. For PVN OT/CTB counts, two sections were selected from the caudal PVN where CTB labeling was found, and bilateral counts were made. For nA counts, all FluoroGold-labeled or ChAT-labeled sections (approximately five or six per series) were counted. PreBötC counts were based on the location of the nA, ventricular aperture, slice shape and marker selection, resulting in a set of slices containing preBötC neurons (approximately three slices per series). Total cell numbers were estimated by multiplying counts per series by two or four, accordingly. RNAscope counts were bilateral in three preBötC sections per mouse.

Details regarding the analysis of the HR components (mHR and RespHRV) and the stability of RespHRV are shown in Supplementary Figs. 1 and 2. HR was obtained from ECG or blood pressure recordings by detecting R-waves or systolic blood pressure, respectively, using Spike2 software (v.10, Cambridge Electrical Design). All recordings were obtained at a minimum sampling rate of 1,000 Hz (freely moving mice: ECG recordings, 1,000 Hz; respiratory activity recordings, 2,000 Hz; blood pressure recordings, 2,000 Hz; anesthetized mice and rats: EMG and ECG recordings, 3,000 Hz; WHBP: all nerve recordings, 3,000 Hz; perfusion pressure recordings, 1,000 Hz). The mHR was quantified as the average of HR over each period analyzed. RespHRV amplitude was calculated from respiratory-triggered averaging of HR aligned to end-inspiration. For each experimental condition, the number of averaged respiratory cycles was determined based on the duration of the effect observed during optogenetic, pharmacological or behavioral stimulation. For optogenetic experiments, the last 30 s of each 1-min stimulation, during which the effects were maximal and stabilized, were analyzed. This period corresponds to a minimum of 40 respiratory cycles for freely moving mice and 15 respiratory cycles for anesthetized mice. In pharmacological experiments with TGOT injections, the effects were maximal during a 30 s window, corresponding to at least 30 respiratory cycles for anesthetized rats and 15 inspiratory discharges for WHBP experiments. For chemogenetic experiments, stress effects were quantified within the first 5 min after the restraint period, over at least 100 respiratory cycles. In all experimental conditions, the analyses of control and recovery periods were performed over a duration that matched or exceeded those mentioned above.

No statistical methods were used to pre-determine sample sizes, but our sample sizes are similar to those reported in previous publications^14,44,46,73^. Data collection and analysis were not performed blind to the conditions of the experiments.

Delta values in percentages (%) show the change in post-stimulus values relative to pre-stimulus values. Statistics on delta values to determine the effect of a given stimulus were performed on absolute values (raw data) when comparing the pre-stimulus and stimulus conditions. Group data are presented as means ± s.e.m. when in numerical values, and as violin plots (truncated or untruncated) with median and quartiles or histograms with mean ± s.e.m. (Extended Data Fig. 7) on graphs. Normality (Shapiro–Wilk test) and variance equality were assessed. Tests were chosen accordingly based on whether assumptions were met. The statistical tests used and post hoc analysis performed are detailed in the corresponding figure legends and in Supplementary Table 1. All statistical analyses were performed using GraphPad Prism (v.9.5.1; GraphPad Software). Statistical significance was set at *P* < 0.05, with *P* values between 0.05 and 0.1 reported in brackets.

If technical issues affected specific recorded parameters, only those were excluded. Animals were excluded only if the injection site, virus expression or optical fiber placement were inaccurate. Animals were randomly selected from littermates and assigned to groups; no further randomization was applicable.

### Reporting summary

Further information on research design is available in the Nature Portfolio Reporting Summary linked to this article.

## Online content

Any methods, additional references, Nature Portfolio reporting summaries, source data, extended data, supplementary information, acknowledgements, peer review information; details of author contributions and competing interests; and statements of data and code availability are available at 10.1038/s41593-025-02074-2.

## Supplementary information

Supplementary Information Supplementary Figs. 1 and 2, and Table 1.

Reporting Summary

Supplementary Data 1 Source data for the data presented in Supplementary Fig. 2.

## Source data

Source Data Fig. 1 Statistical source data.

Source Data Fig. 2 Statistical source data.

Source Data Fig. 3 Statistical source data.

Source Data Fig. 4 Statistical source data.

Source Data Fig. 5 Statistical source data.

Source Data Fig. 6 Statistical source data.

Source Data Fig. 7 Statistical source data.

Source Data Extended Data Fig. 1 Statistical source data.

Source Data Extended Data Fig. 3 Statistical source data.

Source Data Extended Data Fig. 4 Statistical source data.

Source Data Extended Data Fig. 5 Statistical source data.

Source Data Extended Data Fig. 6 Statistical source data.

Source Data Extended Data Fig. 7 Statistical source data.

Source Data Extended Data Fig. 9 Statistical source data.

Source Data Extended Data Fig. 10 Statistical source data.

## Data Availability

All individual data are provided with this paper, and the complete dataset is available as source data files associated with each corresponding figure and extended data figure. Atlases used in this study are published anatomical refs. ^68,69^ and not publicly accessible datasets. Source data are provided with this paper.
