# Supplementary material for: Oxytocin modulates respiratory heart rate variability through a hypothalamus–brainstem–heart neuronal pathway
Source: Nat Neurosci. 2025 Oct 20;28(11):2247–61. doi: 10.1038/s41593-025-02074-2 (PMC12586189; doi:10.1038/s41593-025-02074-2)
Supplement: Supplementary file 1 — Supplementary Figs. 1 and 2, and Table 1. [file 41593_2025_2074_MOESM1_ESM.pdf]

nature neuroscience

Supplementary information

https://doi.org/10.1038/s41593-025-02074-2

Oxytocin modulates respiratory heart rate variability through a hypothalamus–brainstem–heart neuronal pathway

In the format provided by the authors and unedited

Supplementary figures and table

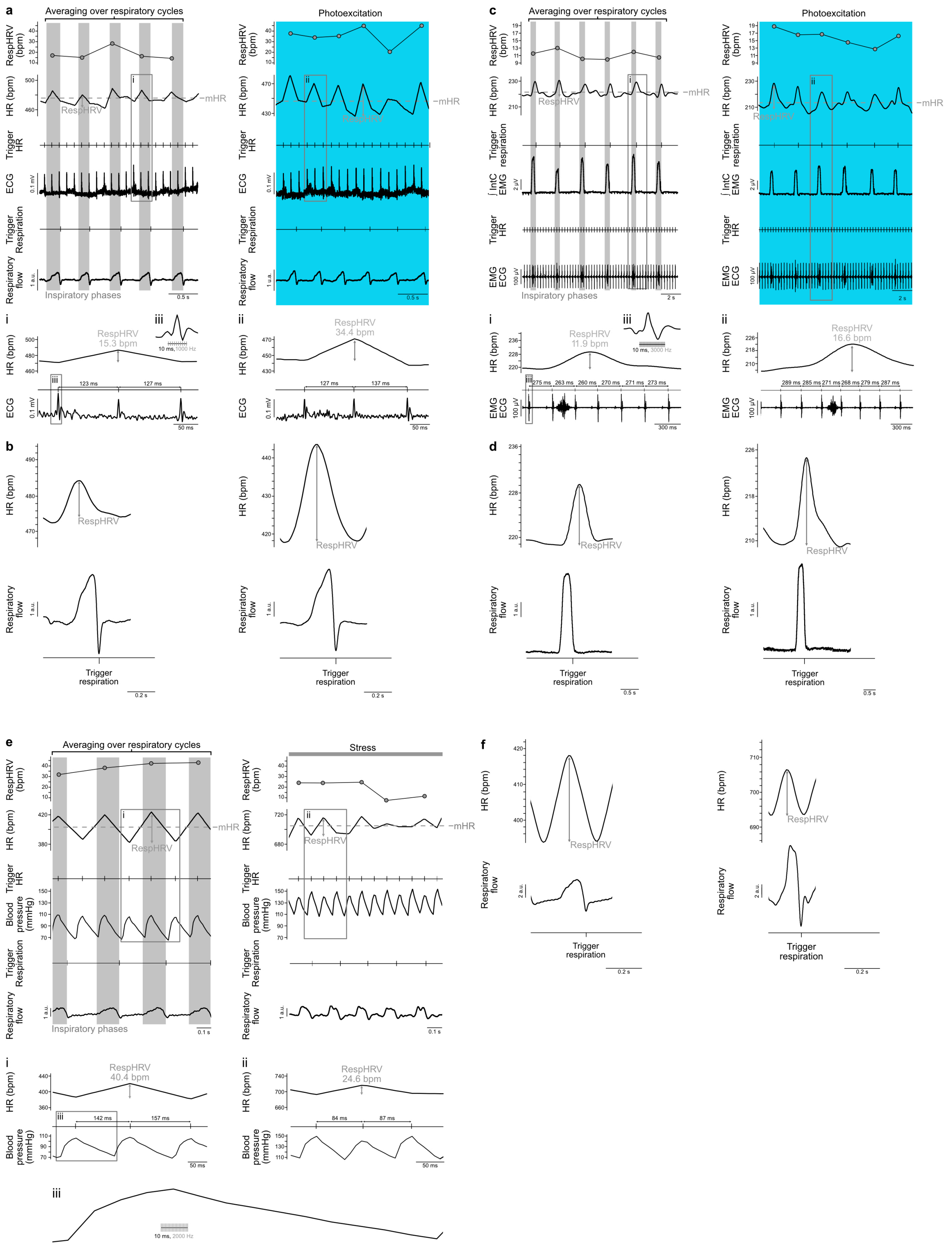

Supplementary Fig. 1: Method for the analysis of mean heart rate (mHR) and respiratory heart rate variability (RespHRV).

a, Recordings of respiratory activity (plethysmography) and HR (electrocardiogram (ECG) telemetry) of a freely moving OT::Cre;Ai27(LSL-ChR2) mouse before and during photoexcitation of OT fibers in the preBötC/nA (Fig. 1e). R-waves on the ECG recordings were automatically detected, manually verified, and labeled "trigger HR". The instantaneous frequency HR trace, in beats per minute (bpm), was then automatically calculated (60 / interval in seconds between each trigger HR). mHR was calculated as the average HR over each period analyzed (gray dotted line on HR traces), and RespHRV was calculated as the difference between maximal HR during inspiration and minimal HR during expiration within each respiratory cycle (gray double arrow on HR traces). Inspiratory phases are represented by the gray shaded areas and "trigger respiration" indicates the end of inspiration. The RespHRV calculated for each respiratory cycle is shown on the top trace. Open gray rectangles correspond to the recordings expanded in i and ii, showing the HR variation and RespHRV over one respiratory cycle. iii represents an expanded ECG recording of a single heartbeat with the acquisition sampling rate of 1000 Hz. **b**, To reduce the endogenous RespHRV variability from one respiratory cycle to the next, RespHRV quantifications were made after respiratory triggered averaging of HR recordings over at least 15 respiratory cycles, using the respiratory trigger shown in (**a**). **c-d**, Same arrangement as in (**a**) and (**b**) except that data were obtained from an anesthetized OT::Cre;Ai27(LSL-ChR2) mouse (Fig. 2b). ECG and electromyogram (EMG) recordings were obtained using subcutaneous electrodes. iii represents the ECG recorded for one heartbeat with the acquisition sampling rate of 3000 Hz. **e-f**, Same arrangement as in (**a**) and (**b**) except that data were obtained from a freely moving OT::Cre;R26-LSL-hM4Di-DREADD mouse before and during a restraint stress test (Fig. 7d). Respiratory activity was recorded by plethysmography, and blood pressure was recorded with telemetry probes. To obtain HR, the systolic blood pressure was automatically detected, manually verified, and labeled "trigger HR". iii represents the blood pressure variation over a single cardiac cycle with the sampling rate of 2000 Hz.

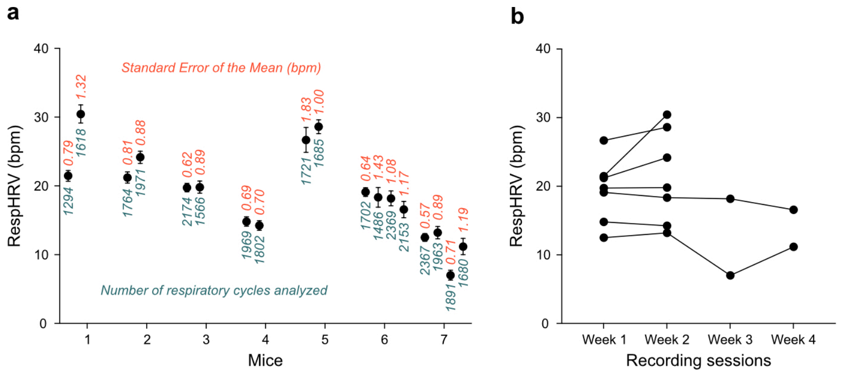

Supplementary Fig. 2: Stability of RespHRV amplitude during quiet breathing.

a-b, RespHRV measured during quiet breathing periods in single recording sessions (~3 h around midday) over two (n = 5) or four (n = 2) consecutive weeks in WT mice using plethysmography (respiratory activity) and telemetry probes (ECG or blood pressure). RespHRV remains stable in each mouse during a recording session, as shown by its little variability (Standard Error of the Mean, SEM) over more than 1,000 respiratory cycles analyzed (**a**). RespHRV also remains stable in each mouse over two to four weeks (**b**). Week 1 *vs*. week 2, Wilcoxon matched-pairs signed rank test (**b**). Detailed statistics are presented in Supplementary Table 1.

Supplementary Table 1: Statistical table for Main Figures, Extended Data Figures and Supplementary Figure 2.

Figures and panels	Type of data	Comparison	Test used	Number of animals	p-values	Mean	SEM
**1a**	Relative values	OT fibers nA *vs.* preBötC (Absolute values in Ext. Data Fig. 1d)	n/a	n = 6	n/a	nA, 37.38 %<br>preBötC, 62.64 %	nA, 4.85 %<br>preBötC, 4.85 %
**1c**	Relative values	Colocalization OT and ChR2	n/a	n = 1	n/a	$OT^{+}ChR2^{+}$, 89.13 %<br>$OT^{+}ChR2^{-}$, 10.87 %<br>$ChR2^{+}OT^{+}$, 96.85 %<br>$ChR2^{+}OT^{-}$, 3.15 %	n/a
**1f**	Absolute values	pre-photoexcitation (Pre-Off) *vs.* Photoexcitation (On) *vs.* Post-photoexcitation (Post-Off)	Repeated Measures one-way ANOVA, Tukey's multiple comparison	n = 8	**RespHRV**:<br>Pre-Off *vs.* On, p = 0.0022<br>Pre-Off *vs.* Post-Off, p > 0.1<br>On *vs.* post-Off, p = 0.0081<br>**mHR:**<br>Pre-Off *vs.* On, p < 0.0001<br>Pre-Off *vs.* Post-Off, p > 0.1<br>On *vs.* post-Off, p = 0.0093<br>**Respiratory freq:**<br>Pre-Off *vs.* On, p = 0.0488<br>Pre-Off *vs.* Post-Off, p = 0.0402<br>On *vs.* post-Off, p > 0.1<br>**Respiratory ampl:**<br>Pre-Off *vs.* On, p > 0.1<br>Pre-Off *vs.* Post-Off, p > 0.1<br>On *vs.* post-Off, p > 0.1	**RespHRV:**<br>Pre-Off, 13.23 bpm<br>On, 19.53 bpm<br>Post-Off, 13.50 bpm<br>**mHR:**<br>Pre-Off, 531.74 bpm<br>On, 497.05 bpm<br>Post-Off, 519.44 bpm<br>**Respiratory freq:**<br>Pre-Off, 124.91 cpm<br>On, 130.64 cpm<br>Post-Off, 132.48 cpm<br>**Respiratory ampl:**<br>Pre-Off, 1.76 (a.u.)<br>On, 1.71 (a.u.)<br>Post-Off, 1.65 (a.u.)	**RespHRV:**<br>Pre-Off, 3.24 bpm<br>On, 4.36 bpm<br>Post-Off, 3.22 bpm<br>**mHR:**<br>Pre-Off, 18.47 bpm<br>On, 18.99 bpm<br>Post-Off, 20.75 bpm<br>**Respiratory freq:**<br>Pre-Off, 8.74 cpm<br>On, 8.89 cpm<br>Post-Off, 8.23 cpm<br>**Respiratory ampl:**<br>Pre-Off, 0.23 (a.u.)<br>On, 0.22 (a.u.)<br>Post-Off, 0.22 (a.u.)
**1g**	Delta changes	Intra-group analysis from 1f: pre-photoexcitation (Pre-Off) *vs.* Photoexcitation (On)	Repeated Measures one-way ANOVA, Tukey's multiple comparison (test performed on pre-photoexcitation (Pre-Off) *vs.* photoexcitation	n = 8	**RespHRV**:<br>Pre-Off *vs.* On, p = 0.0022<br><br>**mHR:**<br>Pre-Off *vs.* On, p < 0.0001<br><br>**Respiratory freq:**<br>Pre-Off *vs.* On, p = 0.0488	**RespHRV:**<br>Pre-Off, 13.23 bpm<br>On, 19.53 bpm<br>**mHR:**<br>Pre-Off, 531.74 bpm<br>On, 497.05 bpm<br>**Respiratory freq:**<br>Pre-Off, 124.91 cpm<br>On, 130.64 cpm	**RespHRV:**<br>Pre-Off, 3.24 bpm<br>On, 4.36 bpm<br>**mHR:**<br>Pre-Off, 18.47 bpm<br>On, 18.99 bpm<br>**Respiratory freq:**<br>Pre-Off, 8.74 cpm<br>On, 8.89 cpm

			(On) *vs.* post-photoexcitation (Post-Off) data)		**Respiratory ampl:**<br>Pre-Off *vs.* On, $p > 0.1$	**Respiratory ampl:**<br>Pre-Off, 1.76 (a.u.)<br>On, 1.71 (a.u.)	**Respiratory ampl:**<br>Pre-Off, 0.23 (a.u.)<br>On, 0.22 (a.u.)
**1g**	Delta changes	Inter-group analysis: Cre^+ *vs.* Cre^-	Unpaired t-test	Cre^+, n = 8;<br>Cre^-, n = 4	**RespHRV,** $p < 0.0001$<br><br><br>**mHR,** p = 0.0003<br><br><br>**Respiratory freq,** p = 0.0305<br><br><br>**Respiratory ampl,** $p > 0.1$	**RespHRV**:<br>Cre^+, +56.30 %<br>Cre^-, +0.66 %<br>**mHR**<br>Cre^+, -34.69 bpm<br>Cre^-, -3.95 bpm<br>**Respiratory freq:**<br>Cre^+, +4.81 %<br>Cre^-, -2.24 %<br>**Respiratory ampl:**<br>Cre^+, -3.44 %<br>Cre^-, -4.48 %	**RespHRV**:<br>Cre^+, 6.03 %<br>Cre^-, 0.60 %<br>**mHR**<br>Cre^+, 3.04 bpm<br>Cre^-, 5.19 bpm<br>**Respiratory freq:**<br>Cre^+, 1.75 %<br>Cre^-, 2.17 %<br>**Respiratory ampl:**<br>Cre^+, 2.10 %<br>Cre^-, 2.12 %
**1h**	Absolute values *vs.* delta changes	Pre-stim RespHRV *vs.* Delta RespHRV<br><br>Pre-stim mHR *vs.* Delta mHR	Pearson correlation analysis, simple linear regression plotted	n = 8	**Pre-stim RespHRV *vs.* Delta RespHRV:**<br>$p < 0.0001$, $R^2 = 97$ %<br><br>**Pre-stim mHR *vs.* Delta mHR:**<br>p = 0.8348, $R^2 = 0.7$ %	n/a	n/a
**Ext. Data Fig. 1d**	Absolute values	preBötC *vs.* nA	Paired t-test	n = 6	p = 0.0299	preBötC, 9.50 fibers<br>nA, 5.67 fibers	preBötC, 1.06 fibers<br>nA, 1.15 fibers
**Ext. Data Fig. 1e**	Relative values	Colocalization CTB and OT	n/a	n = 2	n/a	$PS38^+$ CTB^+, 35.11 %<br>$PS38^+$ CTB^-, 64.89 %<br>CTB^+ $PS38^+$, 29.45 %<br>CTB^+ $PS38^-$, 70.55 %	$PS38^+$ CTB^+, 1.91 %<br>$PS38^+$ CTB^-, 1.91 %<br>CTB^+ $PS38^+$, 7.59 %<br>CTB^+ $PS38^-$, 7.59 %
**2c**	Delta changes	Intra-group analysis from Ext. Data Fig. 3a: pre-photoexcitation (Pre-Off) *vs.* Photoexcitation (On)	Repeated Measures one-way ANOVA, Tukey's multiple comparison (test performed on pre-photoexcitation (Pre-Off) *vs.* photoexcitation	Cre^+ females, n = 15;<br>Cre^+ males, n = 15;<br>Cre^-, n = 5	**RespHRV**:<br>Cre^+ Females, Pre-Off *vs.* On, $p < 0.0001$<br>Cre^+ Males, Pre-Off *vs.* On, $p < 0.0001$<br>Cre^-, Pre-Off *vs.* On, $p > 0.1$	**RespHRV:**<br>**Cre^+ Females**<br>Pre-Off, 3.82 bpm<br>On, 5.82 bpm<br>**Cre^+ Males**<br>Pre-Off, 6.13 bpm<br>On, 8.97 bpm<br>**Cre^-**<br>Pre-Off, 4.60 bpm<br>On, 4.52 bpm	**RespHRV:**<br>**Cre^+ Females**<br>Pre-Off, 0.51 bpm<br>On, 0.78 bpm<br>**Cre^+ Males**<br>Pre-Off, 0.93 bpm<br>On, 1.28 bpm<br>**Cre^-**<br>Pre-Off, 3.52 bpm<br>On, 3.05 bpm

			(On) *vs*. post-photoexcitation (Post-Off) data)		**mHR:**<br>Cre$^+$ Females, Pre-Off *vs*. On, p = 0.0001<br>Cre$^+$ Males, Pre-Off *vs*. On, p < 0.0001<br>Cre$^-$, Pre-Off *vs*. On, p > 0.1<br><br><br><br><br><br><br>**Respiratory freq:**<br>Cre$^+$ Females, Pre-Off *vs*. On, p > 0.1<br>Cre$^+$ Males, Pre-Off *vs*. On, p > 0.1<br>Cre$^-$, Pre-Off *vs*. On, p > 0.1<br><br><br><br><br><br><br>**Respiratory ampl:**<br>Cre$^+$ Females, Pre-Off *vs*. On, p = 0.0039<br>Cre$^+$ Males, Pre-Off *vs*. On, p > 0.1<br>Cre$^-$, Pre-Off *vs*. On, p > 0.1	**mHR:**<br>**Cre$^+$ Females**<br>Pre-Off, 269.48 bpm<br>On, 264.54 bpm<br>**Cre$^+$ Males**<br>Pre-Off, 214.12 bpm<br>On, 207.75 bpm<br>**Cre$^-$**<br>Pre-Off, 249.79 bpm<br>On, 249.83 bpm<br>**Respiratory freq:**<br>**Cre$^+$ Females**<br>Pre-Off, 32.62 cpm<br>On, 31.64 cpm<br>**Cre$^+$ Males**<br>Pre-Off, 24.39 cpm<br>On, 24.54 cpm<br>**Cre$^-$**<br>Pre-Off, 34.98 bpm<br>On, 34.86 bpm<br>**Respiratory ampl:**<br>**Cre$^+$ Females**<br>Pre-Off, 6.40 (a.u.)<br>On, 6.94 (a.u.)<br>**Cre$^+$ Males**<br>Pre-Off, 4.80 (a.u.)<br>On, 5.03 (a.u.)<br>**Cre$^-$**<br>Pre-Off, 4.63 (a.u.)<br>On, 4.73 (a.u.)	**mHR:**<br>**Cre$^+$ Females**<br>Pre-Off, 11.91 bpm<br>On, 12.04 bpm<br>**Cre$^+$ Males**<br>Pre-Off, 6.64 bpm<br>On, 6.60 bpm<br>**Cre$^-$**<br>Pre-Off, 18.83 bpm<br>On, 16.69 bpm<br>**Respiratory freq:**<br>**Cre$^+$ Females**<br>Pre-Off, 2.29 cpm<br>On, 2.27 cpm<br>**Cre$^+$ Males**<br>Pre-Off, 1.31 cpm<br>On, 1.26 cpm<br>**Cre$^-$**<br>Pre-Off, 5.12 bpm<br>On, 4.17 bpm<br>**Respiratory ampl:**<br>**Cre$^+$ Females**<br>Pre-Off, 0.54 (a.u.)<br>On, 0.62 (a.u.)<br>**Cre$^+$ Males**<br>Pre-Off, 0.34 (a.u.)<br>On, 0.34 (a.u.)<br>**Cre$^-$**<br>Pre-Off, 0.95 (a.u.)<br>On, 0.97 (a.u.)
**2c**	Delta changes	Inter-group analysis: Cre$^+$ females *vs*. Cre$^+$ males *vs*. Cre$^-$	One-way ANOVA, Tukey's multiple comparison (RespHRV, mHR, respiratory frequency) or Kruskal-Wallis	Cre$^+$ females, n = 15;<br>Cre$^+$ males, n = 15;<br>Cre$^-$, n = 5	**RespHRV,** p < 0.0001<br><br><br><br>**mHR,** p = 0.0296	**RespHRV**:<br>Cre$^+$ females, +53.37 %<br>Cre$^+$ males, +50.00 %<br>Cre$^-$, -1.39 %<br>**mHR:**<br>Cre$^+$ females, -4.59 bpm<br>Cre$^+$ males, -6.37 bpm	**RespHRV**:<br>Cre$^+$ females, 3.68 %<br>Cre$^+$ males, 4.87 %<br>Cre$^-$, 5.31 %<br>**mHR:**<br>Cre$^+$ females, 0.85 bpm<br>Cre$^+$ males, 0.99 bpm<br>Cre$^-$, 0.27 bpm

			test, Dunn's multiple comparison (respiratory amplitude)		**Respiratory freq,** $p > 0.1$<br><br>**Respiratory ampl,** $p > 0.1$	Cre^{-}, +0.05 bpm<br>**Respiratory freq:**<br>Cre^{+} females, -0.88 %<br>Cre^{+} males, +0.99 %<br>Cre^{-}, +0.21 %<br>**Respiratory ampl:**<br>Cre^{+} females, +7.96 %<br>Cre^{+} males, +5.96 %<br>Cre^{-}, +1.11 %	**Respiratory freq:**<br>Cre^{+} females, 0.86 %<br>Cre^{+} males, 1.19 %<br>Cre^{-}, 1.75 %<br>**Respiratory ampl:**<br>Cre^{+} females, 1.72 %<br>Cre^{+} males, 2.18 %<br>Cre^{-}, 2.60 %
**2d**	Absolute values *vs.* delta changes	Pre-stim RespHRV *vs.* Delta RespHRV<br><br>Pre-stim mHR *vs.* Delta mHR	Pearson correlation analysis, simple linear regression plotted	Cre^{+} females, n = 15;<br>Cre^{+} males, n = 15	**Pre-stim RespHRV vs. Delta RespHRV:**<br>$p < 0.0001$, $R^2 = 75$ %<br><br>**Pre-stim mHR vs. Delta mHR:**<br>$p = 0.5179$, $R^2 = 2$ %	n/a	n/a
**2g**	Delta changes	Intra-group analysis from Ext. Data Fig. 3c-d: pre-photoexcitation (Pre-Off) *vs.* Photoexcitation (On)	Repeated Measures one-way ANOVA, Tukey's multiple comparison (test performed on pre-photoexcitation (Pre-Off) *vs.* photoexcitation (On) *vs.* post-photoexcitation (Post-Off) data)	"ipsi" and "OT-R antagonist + ipsi", n = 9;<br>"OT-R antagonist + contra", n = 6;<br>Vehicle injections, n = 5	**RespHRV:**<br>"Ipsi before OT-R antagonist", $p < 0.0001$<br>"OT-R antagonist + ipsi", $p > 0.1$<br>"OT-R antagonist + contra", $p = 0.0004$<br>"Ipsi before vehicle", $p = 0.0293$<br>"vehicle + ipsi", $p = 0.0009$<br><br>**mHR:**<br>"Ipsi before OT-R antagonist", $p = 0.0029$<br>"OT-R antagonist + ipsi", $p = 0.0339$<br>"OT-R antagonist + contra", $p = 0.0134$	**RespHRV:**<br>**"Ipsi before OT-R antagonist"**<br>Pre-Off, 3.54 bpm<br>On, 5.04 bpm<br>**"OT-R antagonist + ipsi"**<br>Pre-Off, 5.18 bpm<br>On, 5.53 bpm<br>**"OT-R antagonist + contra"**<br>Pre-Off, 3.63 bpm<br>On, 5.58 bpm<br>**"Ipsi before vehicle"**<br>Pre-Off, 5.49 bpm<br>On, 8.24 bpm<br>**"vehicle + ipsi"**<br>Pre-Off, 3.91 bpm<br>On, 5.51 bpm<br><br>**mHR:**<br>**"Ipsi before OT-R antagonist"**<br>Pre-Off, 245.68 bpm	**RespHRV:**<br>**"Ipsi before OT-R antagonist"**<br>Pre-Off, 0.71 bpm<br>On, 0.90 bpm<br>**"OT-R antagonist + ipsi"**<br>Pre-Off, 1.38 bpm<br>On, 1.31 bpm<br>**"OT-R antagonist + contra"**<br>Pre-Off, 0.66 bpm<br>On, 0.95 bpm<br>**"Ipsi before vehicle"**<br>Pre-Off, 2.50 bpm<br>On, 3.51 bpm<br>**"vehicle + ipsi"**<br>Pre-Off, 0.86 bpm<br>On, 1.10 bpm<br><br>**mHR:**<br>**"Ipsi before OT-R antagonist"**<br>Pre-Off, 15.72 bpm

					"Ipsi before vehicle", p = 0.0293<br>"vehicle + ipsi", p = 0.0734	On, 241.29 bpm<br>**"OT-R antagonist + ipsi"**<br>Pre-Off, 260.61 bpm<br>On, 257.64 bpm<br>**"OT-R antagonist + contra"**<br>Pre-Off, 288.06 bpm<br>On, 285.38 bpm<br>**"Ipsi before vehicle"**<br>Pre-Off, 250.32 bpm<br>On, 246.55 bpm<br>**"vehicle + ipsi"**<br>Pre-Off, 272.1 b1pm<br>On, 270.30 bpm	On, 16.10 bpm<br>**"OT-R antagonist + ipsi"**<br>Pre-Off, 18.13 bpm<br>On, 18.91 bpm<br>**"OT-R antagonist + contra"**<br>Pre-Off, 13.72 bpm<br>On, 13.26 bpm<br>**"Ipsi before vehicle"**<br>Pre-Off, 17.82 bpm<br>On, 17.85 bpm<br>**"vehicle + ipsi"**<br>Pre-Off, 22.22 bpm<br>On, 21.89 bpm
**2g**	Delta changes (OT-R antagonist)	Inter-group analysis: "ipsi" *vs.* "OT-R antagonist + ipsi" *vs.* "OT-R antagonist + contra"	Repeated Measures mixed-effects analysis with the Geisser-Greenhouse correction, Tukey's multiple comparison	"ipsi" and "OT-R antagonist + ipsi",<br>n = 9;<br>"OT-R antagonist + contra", n = 6	**RespHRV:**<br>"ipsi" *vs.* "OT-R antagonist + ipsi", p = 0.0003<br>"OT-R antagonist + ipsi" *vs.* "OT-R antagonist + contra", p = 0.0016<br>"ipsi" *vs.* "OT-R antagonist + contra", p > 0.1<br><br>**mHR:**<br>"ipsi" *vs.* "OT-R antagonist + ipsi", p > 0.1<br>"OT-R antagonist+ ipsi" *vs.* "OT-R antagonist + contra", p > 0.1<br>"ipsi" *vs.* "OT-R antagonist + contra", p > 0.1	**RespHRV:**<br>"ipsi", +48.84 %<br>"OT-R antagonist + ipsi", +13.34 %<br>"OT-R antagonist + contra", +59.99 %<br><br>**mHR:**<br>"ipsi", -4.39 bpm<br>"OT-R antagonist + ipsi", -2.97 bpm<br>"OT-R antagonist + contra", -2.68 bpm	**RespHRV:**<br>"ipsi", 7.80 %<br>"OT-R antagonist + ipsi", 4.99 %<br>"OT-R antagonist + contra", 10.74 %<br><br>**mHR:**<br>"ipsi", 1.18 bpm<br>"OT-R antagonist + ipsi", 1.25 bpm<br>"OT-R antagonist + contra", 0.76 bpm
**2g**	Delta changes (vehicle)	Inter-group analysis: "ipsi" *vs.* "vehicle + ipsi"	Paired t-test	n = 5	**RespHRV,** p > 0.1<br><br><br>**mHR,** p > 0.1	**RespHRV:**<br>"ipsi", +53.82 %<br>"vehicle + ipsi", +43.48 %<br>**mHR:**<br>"ipsi", -3.77 bpm<br>"vehicle + ipsi", -1.81 bpm	**RespHRV:**<br>"ipsi", 5.40 %<br>"vehicle + ipsi", 4.96 %<br><br>**mHR:**<br>"ipsi", 1.18 bpm<br>"vehicle + ipsi", 0.93 bpm

**Ext. Data Fig. 3a**	Absolute values (Cre+)	pre-photoexcitation (Pre-Off) *vs.* Photoexcitation (On) *vs.* Post-photoexcitation (Post-Off)	Repeated Measures two-way ANOVA, Sidak's multiple comparison	Cre+ females, n = 15;<br>Cre+ males, n=15	**RespHRV**:<br>**Females**<br>Pre-Off *vs.* On, $p < 0.0001$<br>Pre-Off *vs.* Post-Off, $p > 0.1$<br>On *vs.* post-Off, $p < 0.0001$<br>**Males**<br>Pre-Off *vs.* On, $p < 0.0001$<br>Pre-Off *vs.* Post-Off, $p > 0.1$<br>On *vs.* post-Off, $p < 0.0001$<br>**Females vs. Males**<br>Pre-Off F *vs.* Pre-Off M, $p > 0.1$<br>Pre-Off F *vs.* On M, $p > 0.1$<br>Pre-Off F *vs.* Post-Off M, $p > 0.1$<br>On F *vs.* Pre-off M, $p > 0.1$<br>On F *vs.* On M, $p > 0.1$<br>On F *vs.* Post-off M, $p > 0.1$<br>Post-off F *vs.* Pre-off M, $p > 0.1$<br>Post-off F *vs.* On M, $p > 0.1$<br>Post-off F *vs.* Post-off M, $p > 0.1$<br><br>**mHR:**<br>**Females**<br>Pre-Off *vs.* On, $p = 0.0001$<br>Pre-Off *vs.* Post-Off, $p > 0.1$<br>On *vs.* post-Off, $p = 0.0012$<br>**Males**<br>Pre-Off *vs.* On, $p < 0.0001$<br>Pre-Off *vs.* Post-Off, $p > 0.1$<br>On *vs.* post-Off, $p = 0.0001$<br>**Females vs. Males**<br>Pre-Off F *vs.* Pre-Off M, $p = 0.0018$<br>Pre-Off F *vs.* On M, $p > 0.1$<br>Pre-Off F *vs.* Post-Off M, $p > 0.1$<br>On F *vs.* Pre-off M, $p > 0.1$<br>On F *vs.* On M, $p = 0.0015$<br>On F *vs.* Post-off M, $p > 0.1$<br>Post-off F *vs.* Pre-off M, $p > 0.1$<br>Post-off F *vs.* On M, $p > 0.1$	**RespHRV:**<br>**Females**<br>Pre-Off, 3.82 bpm<br>On, 5.82 bpm<br>Post-Off, 4.06 bpm<br>**Males**<br>Pre-Off, 6.13 bpm<br>On, 8.97 bpm<br>Post-Off, 6.10 bpm<br><br>**mHR:**<br>**Females**<br>Pre-Off, 269.48 bpm<br>On, 264.54 bpm<br>Post-Off, 269.75 bpm<br>**Males**<br>Pre-Off, 214.12 bpm<br>On, 207.75 bpm<br>Post-Off, 214.01 bpm	**RespHRV:**<br>**Females**<br>Pre-Off, 0.51 bpm<br>On, 0.78 bpm<br>Post-Off, 0.57 bpm<br>**Males**<br>Pre-Off, 0.93 bpm<br>On, 1.28 bpm<br>Post-Off, 0.96 bpm<br><br>**mHR:**<br>**Females**<br>Pre-Off, 11.91 bpm<br>On, 12.04 bpm<br>Post-Off, 11.83 bpm<br>**Males**<br>Pre-Off, 6.64 bpm<br>On, 6.60 bpm<br>Post-Off, 6.70 bpm

					Post-off F *vs*. Post-off M, p = 0.0016		
					**Respiratory freq:**<br>**Females**<br>Pre-Off *vs*. On, p > 0.1<br>Pre-Off *vs*. Post-Off, p > 0.1<br>On *vs*. post-Off, p > 0.1<br>**Males**<br>Pre-Off *vs*. On, p > 0.1<br>Pre-Off *vs*. Post-Off, p > 0.1<br>On *vs*. post-Off, p > 0.1<br>**Females vs. Males**<br>Pre-Off F *vs*. Pre-Off M, p = 0.0149<br>Pre-Off F *vs*. On M, p > 0.1<br>Pre-Off F *vs*. Post-Off M, p > 0.1<br>On F *vs*. Pre-off M, p > 0.1<br>On F *vs*. On M, p = 0.0359<br>On F *vs*. Post-off M, p > 0.1<br>Post-off F *vs*. Pre-off M, p > 0.1<br>Post-off F *vs*. On M, p > 0.1<br>Post-off F *vs*. Post-off M, p = 0.0202	**Respiratory freq:**<br>**Females**<br>Pre-Off, 32.62 cpm<br>On, 31.64 cpm<br>Post-Off, 32.81 cpm<br>**Males**<br>Pre-Off, 24.39 cpm<br>On, 24.54 cpm<br>Post-Off, 24.59 cpm	**Respiratory freq:**<br>**Females**<br>Pre-Off, 2.29 cpm<br>On, 2.27 cpm<br>Post-Off, 2.43 cpm<br>**Males**<br>Pre-Off, 1.31 cpm<br>On, 1.26 cpm<br>Post-Off, 1.25 cpm
					**Respiratory ampl:**<br>**Females**<br>Pre-Off *vs*. On, p = 0.0044<br>Pre-Off *vs*. Post-Off, p > 0.1<br>On *vs*. post-Off, p = 0.0394<br>**Males**<br>Pre-Off *vs*. On, p > 0.1<br>Pre-Off *vs*. Post-Off, p > 0.1<br>On *vs*. post-Off, p > 0.1<br>**Females vs. Males**<br>Pre-Off F *vs*. Pre-Off M, p = 0.0564<br>Pre-Off F *vs*. On M, p > 0.1<br>Pre-Off F *vs*. Post-Off M, p > 0.1<br>On F *vs*. Pre-off M, p > 0.1<br>On F *vs*. On M, p = 0.0400<br>On F *vs*. Post-off M, p > 0.1	**Respiratory ampl:**<br>**Females**<br>Pre-Off, 6.40 (a.u.)<br>On, 6.94 (a.u.)<br>Post-Off, 6.43 (a.u.)<br>**Males**<br>Pre-Off, 4.80 (a.u.)<br>On, 5.03 (a.u.)<br>Post-Off, 4.80 (a.u.)	**Respiratory ampl:**<br>**Females**<br>Pre-Off, 0.54 (a.u.)<br>On, 0.62 (a.u.)<br>Post-Off, 0.62 (a.u.)<br>**Males**<br>Pre-Off, 0.34 (a.u.)<br>On, 0.34 (a.u.)<br>Post-Off, 0.33 (a.u.)

					Post-off F *vs*. Pre-off M, p > 0.1<br>Post-off F *vs*. On M, p > 0.1<br>Post-off F *vs*. Post-off M, p = 0.0860		
**Ext. Data Fig. 3a**	Absolute values (Cre$^-$)	pre-photoexcitation (Pre-Off) *vs.* Photoexcitation (On) vs. Post-photoexcitation (Post-Off)	Repeated Measures one-way ANOVA, Tukey's multiple comparison	n = 5	**RespHRV**:<br>Pre-Off vs. On, p > 0.1<br>Pre-Off vs. Post-Off, p > 0.1<br>On vs. post-Off, p > 0.1<br>**mHR:**<br>Pre-Off vs. On, p > 0.1<br>Pre-Off vs. Post-Off, p > 0.1<br>On vs. post-Off, p > 0.1<br>**Respiratory freq:**<br>Pre-Off vs. On, p > 0.1<br>Pre-Off vs. Post-Off, p > 0.1<br>On vs. post-Off, p > 0.1<br>**Respiratory ampl:**<br>Pre-Off vs. On, p > 0.1<br>Pre-Off vs. Post-Off, p > 0.1<br>On vs. post-Off, p > 0.1	**RespHRV:**<br>Pre-Off, 4.60 bpm<br>On, 4.52 bpm<br>Post-Off, 4.53 bpm<br>**mHR**:<br>Pre-Off, 249.79 bpm<br>On, 249.83 bpm<br>Post-Off, 249.47 bpm<br>**Respiratory freq**:<br>Pre-Off, 34.98 bpm<br>On, 34.86 bpm<br>Post-Off, 33.77 bpm<br>**Respiratory ampl**:<br>Pre-Off, 4.63 (a.u.)<br>On, 4.73 (a.u.)<br>Post-Off, 4.76 (a.u.)	**RespHRV:**<br>Pre-Off, 3.52 bpm<br>On, 3.41 bpm<br>Post-Off, 3.37 bpm<br>**mHR**:<br>Pre-Off, 18.83 bpm<br>On, 18.66 bpm<br>Post-Off, 19.00 bpm<br>**Respiratory freq**:<br>Pre-Off, 5.12 bpm<br>On, 4.67 bpm<br>Post-Off, 4.39 bpm<br>**Respiratory ampl**:<br>Pre-Off, 0.95 (a.u.)<br>On, 1.09 (a.u.)<br>Post-Off, 1.07 (a.u.)
**Ext. Data Fig. 3b**	Absolute values	Before ("Control") *vs*. after "OT-R antagonist" or "Vehicle"	Paired t-test or Wilcoxon matched-pairs signed rank test	OT-R antagonist injections, n = 9; Vehicle injections, n = 5	**RespHRV**:<br>"Control" *vs*. after "OT-R antagonist", p > 0.1<br>"Control" vs. after "Vehicle", p > 0.1<br><br>**mHR**:<br>"Control" *vs*. after "OT-R antagonist", p > 0.1<br>"Control" vs. after "Vehicle", p > 0.1	**RespHRV**:<br>"Control before OT-R antagonist", 3.54 bpm<br>After "OT-R antagonist", 5.18 bpm<br>"Control before vehicle", 5.49 bpm<br>After "Vehicle", 3.91 bpm<br><br>**mHR**:<br>"Control before OT-R antagonist", 245.68 bpm<br>After "OT-R antagonist", 260.61 bpm	**RespHRV**:<br>"Control before OT-R antagonist", 0.71 bpm<br>After "OT-R antagonist", 1.38 bpm<br>"Control before vehicle", 2.50 bpm<br>After "Vehicle", 0.86 bpm<br><br>**mHR**:<br>"Control before OT-R antagonist", 15.72 bpm<br>After "OT-R antagonist", 18.13 bpm<br>"Control before vehicle", 17.82 bpm

						"Control before vehicle", 250.32 bpm<br>After "Vehicle", 271.11 bpm	After "Vehicle", 22.22 bpm
					**Respiratory freq**:<br>"Control" *vs*. after "OT-R antagonist", p = 0.0761<br>"Control" vs. after "Vehicle", p = 0.0625	**Respiratory freq**:<br>"Control before OT-R antagonist", 34.09 cpm<br>After "OT-R antagonist", 29.18 cpm<br>"Control before vehicle", 29.34 bpm<br>After "Vehicle", 25.43 bpm	**Respiratory freq**:<br>"Control before OT-R antagonist", 3.82 cpm<br>After "OT-R antagonist", 3.05 cpm<br>"Control before vehicle", 2.78 bpm<br>After "Vehicle", 1.03 bpm
					**Respiratory ampl**:<br>"Control" *vs*. after "OT-R antagonist", p > 0.1<br>"Control" vs. after "Vehicle", p = 0.0367	**Respiratory ampl**:<br>"Control before OT-R antagonist", 5.65 (a.u.)<br>After "OT-R antagonist", 6.17 (a.u.)<br>"Control before vehicle", 5.72 (a.u.)<br>After "Vehicle", 6.64 (a.u.)	**Respiratory ampl**:<br>"Control before OT-R antagonist", 0.49 (a.u.)<br>After "OT-R antagonist", 0.65 (a.u.)<br>"Control before vehicle", 0.88 (a.u.)<br>After "Vehicle", 0.83 (a.u.)
**Ext. Data Fig. 3c**	Absolute values	pre-photoexcitation (Pre-Off) *vs*. Photoexcitation (On) *vs*. Post-photoexcitation (Post-Off)	Repeated Measures one-way ANOVA, Tukey's multiple comparison	"ipsi" and "OT-R antagonist + ipsi", n = 9;<br>"OT-R antagonist + contra", n = 6;	**RespHRV:**<br>**"ipsilateral"**<br>Pre-Off *vs*. On, p < 0.0001<br>Pre-Off *vs*. Post-Off, p > 0.1<br>On *vs*. post-Off, p < 0.0001<br>**"OT-R antagonist + ipsilateral"**<br>Pre-Off *vs*. On, p > 0.1<br>Pre-Off *vs*. Post-Off, p > 0.1<br>On *vs*. post-Off, p > 0.1<br>**"OT-R antagonist + controlateral"**<br>Pre-Off *vs*. On, p = 0.0004<br>Pre-Off *vs*. Post-Off, p > 0.1<br>On *vs*. post-Off, p = 0.0008	**RespHRV:**<br>**"ipsilateral"**<br>Pre-Off, 3.54 bpm<br>On, 5.04 bpm<br>Post-Off, 3.61 bpm<br>**"OT-R antagonist + ipsilateral"**<br>Pre-Off, 5.18 bpm<br>On, 5.53 bpm<br>Post-Off, 5.13 bpm<br>**"OT-R antagonist + controlateral"**<br>Pre-Off, 3.63 bpm<br>On, 5.58 bpm	**RespHRV:**<br>**"ipsilateral"**<br>Pre-Off, 0.71 bpm<br>On, 0.90 bpm<br>Post-Off, 0.77 bpm<br>**"OT-R antagonist + ipsilateral"**<br>Pre-Off, 1.38 bpm<br>On, 1.31 bpm<br>Post-Off, 1.47 bpm<br>**"OT-R antagonist + controlateral"**<br>Pre-Off, 0.66 bpm<br>On, 0.95 bpm

						Post-Off, 3.82 bpm	Post-Off, 0.65 bpm
					**mHR:**<br>**“ipsilateral”**<br>Pre-Off *vs*. On, p = 0.0029<br>Pre-Off *vs*. Post-Off, p > 0.1<br>On *vs*. post-Off, p < 0.0001<br>**“OT-R antagonist + ipsilateral”**<br>Pre-Off *vs*. On, p = 0.0339<br>Pre-Off *vs*. Post-Off, p > 0.1<br>On *vs*. post-Off, p = 0.0017<br>**“OT-R antagonist + controlateral”**<br>Pre-Off *vs*. On, p = 0.0134<br>Pre-Off *vs*. Post-Off, p > 0.1<br>On *vs*. post-Off, p = 0.0013	**mHR:**<br>**“ipsilateral”**<br>Pre-Off, 245.68 bpm<br>On, 241.29 bpm<br>Post-Off, 247.62 bpm<br>**“OT-R antagonist + ipsilateral”**<br>Pre-Off, 260.61 bpm<br>On, 257.64 bpm<br>Post-Off, 262.18 bpm<br>**“OT-R antagonist + controlateral”**<br>Pre-Off, 288.06 bpm<br>On, 285.38 bpm<br>Post-Off, 289.19 bpm	**mHR:**<br>**“ipsilateral”**<br>Pre-Off, 15.72 bpm<br>On, 16.10 bpm<br>Post-Off, 15.57 bpm<br>**“OT-R antagonist + ipsilateral”**<br>Pre-Off, 18.13 bpm<br>On, 18.91 bpm<br>Post-Off, 18.02 bpm<br>**“OT-R antagonist + controlateral”**<br>Pre-Off, 13.72 bpm<br>On, 13.26 bpm<br>Post-Off, 13.46 bpm
					**Respiratory freq:**<br>**“ipsilateral”**<br>Pre-Off *vs*. On, p > 0.1<br>Pre-Off *vs*. Post-Off, p > 0.1<br>On *vs*. post-Off, p > 0.1<br>**“OT-R antagonist + ipsilateral”**<br>Pre-Off *vs*. On, p > 0.1<br>Pre-Off *vs*. Post-Off, p > 0.1<br>On *vs*. post-Off, p > 0.1<br>**“OT-R antagonist + controlateral”**<br>Pre-Off *vs*. On, p > 0.1<br>Pre-Off *vs*. Post-Off, p > 0.1<br>On *vs*. post-Off, p > 0.1	**Respiratory freq:**<br>**“ipsilateral”**<br>Pre-Off, 34.09 cpm<br>On, 33.63 cpm<br>Post-Off, 34.31 cpm<br>**“OT-R antagonist + ipsilateral”**<br>Pre-Off, 29.18 cpm<br>On, 29.80 cpm<br>Post-Off, 29.55 cpm<br>**“OT-R antagonist + controlateral”**<br>Pre-Off, 30.23 cpm<br>On, 30.61 cpm<br>Post-Off, 30.42 cpm	**Respiratory freq:**<br>**“ipsilateral”**<br>Pre-Off, 3.82 cpm<br>On, 3.63 cpm<br>Post-Off, 4.03 cpm<br>**“OT-R antagonist + ipsilateral”**<br>Pre-Off, 3.05 cpm<br>On, 2.82 cpm<br>Post-Off, 3.09 cpm<br>**“OT-R antagonist + controlateral”**<br>Pre-Off, 3.76 cpm<br>On, 3.08 cpm<br>Post-Off, 4.31 cpm
					**Respiratory ampl:**<br>**“ipsilateral”**<br>Pre-Off *vs*. On, p > 0.1<br>Pre-Off *vs*. Post-Off, p > 0.1	**Respiratory ampl:**<br>**“ipsilateral”**<br>Pre-Off, 5.65 (a.u.)<br>On, 5.99 (a.u.)	**Respiratory ampl:**<br>**“ipsilateral”**<br>Pre-Off, 0.49 (a.u.)<br>On, 0.51 (a.u.)

					On *vs*. post-Off, p = 0.0782<br>**“OT-R antagonist + ipsilateral”**<br>Pre-Off *vs*. On, p > 0.1<br>Pre-Off *vs*. Post-Off, p > 0.1<br>On *vs*. post-Off, p = 0.0021<br>**“OT-R antagonist + controlateral”**<br>Pre-Off *vs*. On, p = 0.0969<br>Pre-Off *vs*. Post-Off, p > 0.1<br>On *vs*. post-Off, p = 0.0530	Post-Off, 5.52 (a.u.)<br>**“OT-R antagonist + ipsilateral”**<br>Pre-Off, 6.17 (a.u.)<br>On, 6.37 (a.u.)<br>Post-Off, 5.97 (a.u.)<br>**“OT-R antagonist + controlateral”**<br>Pre-Off, 5.93 (a.u.)<br>On, 6.35 (a.u.)<br>Post-Off, 5.87 (a.u.)	Post-Off, 0.62 (a.u.)<br>**“OT-R antagonist + ipsilateral”**<br>Pre-Off, 0.65 (a.u.)<br>On, 0.64 (a.u.)<br>Post-Off, 0.64 (a.u.)<br>**“OT-R antagonist + controlateral”**<br>Pre-Off, 0.86 (a.u.)<br>On, 0.96 (a.u.)<br>Post-Off, 0.79 (a.u.)
**Ext. Data Fig. 3d**	Absolute values	pre-photoexcitation (Pre-Off) *vs*. Photoexcitation (On) vs. Post-photoexcitation (Post-Off)	Repeated Measures one-way ANOVA, Tukey’s multiple comparison	n = 5	**RespHRV:**<br>**“ipsilateral”**<br>Pre-Off vs. On, p = 0.0293<br>Pre-Off vs. Post-Off, p > 0.1<br>On vs. post-Off, p = 0.0267<br>**“vehicle + ipsilateral”**<br>Pre-Off vs. On, p = 0.0009<br>Pre-Off vs. Post-Off, p > 0.1<br>On vs. post-Off, p = 0.0014<br><br>**mHR:**<br>**“ipsilateral”**<br>Pre-Off vs. On, p = 0.0293<br>Pre-Off vs. Post-Off, p > 0.1<br>On vs. post-Off, p = 0.0178<br>**“Vehicle + ipsilateral”**<br>Pre-Off vs. On, p = 0.0734<br>Pre-Off vs. Post-Off, p > 0.1<br>On vs. post-Off, p = 0.0451<br><br>**Respiratory freq:**<br>**“ipsilateral”**<br>Pre-Off vs. On, p > 0.1<br>Pre-Off vs. Post-Off, p > 0.1<br>On vs. post-Off, p > 0.1<br>**“vehicle + ipsilateral”**<br>Pre-Off vs. On, p > 0.1	**RespHRV:**<br>**“ipsilateral”**<br>Pre-Off, 5.49 bpm<br>On, 8.24 bpm<br>Post-Off, 5.43 bpm<br>**“vehicle + ipsilateral”**<br>Pre-Off, 3.91 bpm<br>On, 5.51 bpm<br>Post-Off, 4.01 bpm<br><br>**mHR:**<br>**“ipsilateral”**<br>Pre-Off, 250.32 bpm<br>On, 246.55 bpm<br>Post-Off, 250.73 bpm<br>**“vehicle + ipsilateral”**<br>Pre-Off, 272.1 b1pm<br>On, 270.30 bpm<br>Post-Off, 272.34 bpm<br><br>**Respiratory freq:**<br>**“ipsilateral”**<br>Pre-Off, 29.34 cpm<br>On, 29.33 cpm<br>Post-Off, 29.32 cpm<br>**“vehicle + ipsilateral”**<br>Pre-Off, 25.43 cpm	**RespHRV:**<br>**“ipsilateral”**<br>Pre-Off, 2.50 bpm<br>On, 3.51 bpm<br>Post-Off, 2.46 bpm<br>**“vehicle + ipsilateral”**<br>Pre-Off, 0.86 bpm<br>On, 1.10 bpm<br>Post-Off, 0.93 bpm<br><br>**mHR:**<br>**“ipsilateral”**<br>Pre-Off, 17.82 bpm<br>On, 17.85 bpm<br>Post-Off, 18.27 bpm<br>**“vehicle + ipsilateral”**<br>Pre-Off, 22.22bpm<br>On, 21.89 bpm<br>Post-Off, 22.08 bpm<br><br>**Respiratory freq:**<br>**“ipsilateral”**<br>Pre-Off, 2.78 cpm<br>On, 2.94 cpm<br>Post-Off, 2.86 cpm<br>**“vehicle + ipsilateral”**<br>Pre-Off, 1.03 cpm

					Pre-Off vs. Post-Off, p > 0.1<br>On vs. post-Off, p > 0.1<br><br>**Respiratory ampl:**<br>**“ipsilateral”**<br>Pre-Off vs. On, p = 0.0551<br>Pre-Off vs. Post-Off, p > 0.1<br>On vs. post-Off, p > 0.1<br>**“vehicle + ipsilateral”**<br>Pre-Off vs. On, p > 0.1<br>Pre-Off vs. Post-Off, p > 0.1<br>On vs. post-Off, p > 0.1	On, 26.44 cpm<br>Post-Off, 25.60 cpm<br><br>**Respiratory ampl:**<br>**“ipsilateral”**<br>Pre-Off, 5.72 (a.u.)<br>On, 5.93 (a.u.)<br>Post-Off, 5.89 (a.u.)<br>**“vehicle + ipsilateral”**<br>Pre-Off, 6.64 (a.u.)<br>On, 6.73 (a.u.)<br>Post-Off, 6.59 (a.u.)	On, 0.83 cpm<br>Post-Off, 1.19 cpm<br><br>**Respiratory ampl:**<br>**“ipsilateral”**<br>Pre-Off, 0.88 (a.u.)<br>On, 0.92 (a.u.)<br>Post-Off, 0.96 (a.u.)<br>**“vehicle + ipsilateral”**<br>Pre-Off, 0.83 (a.u.)<br>On, 0.81 (a.u.)<br>Post-Off, 0.85 (a.u.)
**Ext. Data Fig. 3e**	Delta changes	Intra-group analysis from Ext. Data Fig. 3-d: pre-photoexcitation (Pre-Off) *vs.* Photoexcitation (On)	Repeated Measures one-way ANOVA, Tukey’s multiple comparison (test performed on pre-photoexcitation (Pre-Off) *vs.* photoexcitation (On) *vs.* post-photoexcitation (Post-Off) data)	“ipsi” and “OT-R antagonist + ipsi”, n = 9; “OT-R antagonist + contra”, n = 6; Vehicle injections, n = 5	**Respiratory freq:**<br>“Ipsi before OT-R antagonist”, p > 0.1<br>“OT-R antagonist + ipsi”, p > 0.1<br>“OT-R antagonist + contra”, p > 0.1<br>“Ipsi before vehicle”, p > 0.1<br>“vehicle + ipsi”, p > 0.1<br><br>**Respiratory ampl:**<br>“Ipsi before OT-R antagonist”, p > 0.1<br>“OT-R antagonist + ipsi”, p > 0.1<br>“OT-R antagonist + contra”, p = 0.0969<br>“Ipsi before vehicle”, p = 0.0551<br>“vehicle + ipsi”, p > 0.1	**Respiratory freq:**<br>**“Ipsi before OT-R antagonist”**<br>Pre-Off, 34.09 cpm<br>On, 33.63 cpm<br>**“OT-R antagonist + ipsi”**<br>Pre-Off, 29.18 cpm<br>On, 29.80 cpm<br>**“OT-R antagonist + contra”**<br>Pre-Off, 30.23 cpm<br>On, 30.61 cpm<br>**“Ipsi before vehicle”**<br>Pre-Off, 29.34 cpm<br>On, 29.33 cpm<br>**“vehicle + ipsi”**<br>Pre-Off, 25.43 cpm<br>On, 26.44 cpm<br><br>**Respiratory ampl:**<br>**“Ipsi before OT-R antagonist”**<br>Pre-Off, 5.65 (a.u.)<br>On, 5.99 (a.u.)	**Respiratory freq:**<br>**“Ipsi before OT-R antagonist”**<br>Pre-Off, 3.82 cpm<br>On, 3.63 cpm<br>**“OT-R antagonist + ipsi”**<br>Pre-Off, 3.05 cpm<br>On, 2.82 cpm<br>**“OT-R antagonist + contra”**<br>Pre-Off, 3.76 cpm<br>On, 3.08 cpm<br>**“Ipsi before vehicle”**<br>Pre-Off, 2.78 cpm<br>On, 2.94 cpm<br>**“vehicle + ipsi”**<br>Pre-Off, 1.03 cpm<br>On, 0.83 cpm<br><br>**Respiratory ampl:**<br>**“Ipsi before OT-R antagonist”**<br>Pre-Off, 0.49 (a.u.)<br>On, 0.51 (a.u.)

						**“OT-R antagonist + ipsi”**<br>Pre-Off, 6.17 (a.u.)<br>On, 6.37 (a.u.)<br>**“OT-R antagonist + contra”**<br>Pre-Off, 5.93 (a.u.)<br>On, 6.35 (a.u.)<br>**“Ipsi before vehicle”**<br>Pre-Off, 5.72 (a.u.)<br>On, 5.93 (a.u.)<br>**“vehicle + ipsi”**<br>Pre-Off, 6.64 (a.u.)<br>On, 6.73 (a.u.)	**“OT-R antagonist + ipsi”**<br>Pre-Off, 0.65 (a.u.)<br>On, 0.64 (a.u.)<br>**“OT-R antagonist + contra”**<br>Pre-Off, 0.86 (a.u.)<br>On, 0.96 (a.u.)<br>**“Ipsi before vehicle”**<br>Pre-Off, 0.88 (a.u.)<br>On, 0.92 (a.u.)<br>**“vehicle + ipsi”**<br>Pre-Off, 0.83 (a.u.)<br>On, 0.81 (a.u.)
**Ext. Data Fig. 3e**	Delta changes (OT-R antagonist)	Inter-group analysis: “ipsi” *vs.* “OT-R antagonist + ipsi” *vs.* “OT-R antagonist + contra”	Repeated Measures mixed-effects analysis with the Geisser-Greenhouse correction, Tukey’s multiple comparison	“ipsi” and “OT-R antagonist + ipsi”, n = 9;<br>“OT-R antagonist + contra”, n = 6;	**Respiratory freq:**<br>“ipsi” *vs.* “OT-R antagonist + ipsi”, p > 0.1<br>“OT-R antagonist + ipsi” *vs.* “OT-R antagonist + contra”, p > 0.1<br>“ipsi” *vs.* “OT-R antagonist + contra”, p > 0.1<br><br>**Respiratory ampl:**<br>“ipsi” *vs.* “OT-R antagonist + ipsi”, p > 0.1<br>“OT-R antagonist+ ipsi” *vs.* “OT-R antagonist + contra”, p > 0.1<br>“ipsi” *vs.* “OT-R antagonist + contra”, p > 0.1	**Respiratory freq:**<br>“ipsi”, -0.81 %<br>“OT-R antagonist + ipsi”, +3.34 %<br>“OT-R antagonist + contra”, +2.59 %<br><br>**Respiratory ampl:**<br>“ipsi”, +6.19 %<br>“OT-R antagonist + ipsi”, +3.67 %<br>“OT-R antagonist + contra”, +6.86 %	**Respiratory freq:**<br>“ipsi”, 1.33 %<br>“OT-R antagonist + ipsi”, 2.49 %<br>“OT-R antagonist + contra”, 2.94 %<br><br>**Respiratory ampl:**<br>“ipsi”, 1.72 %<br>“OT-R antagonist + ipsi”, 1.72 %<br>“OT-R antagonist + contra”, 2.76 %
**Ext. Data Fig. 3e**	Delta changes (vehicle)	Inter-group analysis: “ipsi” *vs.* “vehicle + ipsi”	Paired t-test	n = 5	**Respiratory freq,** p > 0.1<br><br>**Respiratory ampl,** p > 0.1	**Respiratory freq:**<br>“ipsi”, -0.19 %<br>“Vehicle + ipsi”, +4.18 %<br>**Respiratory ampl:**<br>“ipsi”, +3.69 %<br>“Vehicle + ipsi”, +1.53 %	**Respiratory freq:**<br>“ipsi”, 1.06 %<br>“Vehicle + ipsi”, 1.85 %<br>**Respiratory ampl:**<br>“ipsi”, 0.56 %<br>“Vehicle + ipsi”, 2.09 %
**Ext. Data Fig. 4c**	Absolute values	pre-photoexcitation (Pre-Off) *vs.* Photoexcitation (On)	Repeated Measures one-way ANOVA,	n = 5	**RespHRV**:<br>Pre-Off *vs*. On, p > 0.1<br>Pre-Off *vs*. Post-Off, p > 0.1	**RespHRV:**<br>Pre-Off, 1.37 bpm<br>On, 1.32 bpm	**RespHRV:**<br>Pre-Off, 0.43 bpm<br>On, 0.36 bpm

		vs. Post-photoexcitation (Post-Off)	Tukey's multiple comparison, except for mHR, Friedman test, Dunn's multiple comparison		On *vs*. post-Off, p > 0.1<br>**mHR:**<br>Pre-Off *vs*. On, p > 0.1<br>Pre-Off *vs*. Post-Off, p > 0.1<br>On *vs*. post-Off, p > 0.1<br>**Respiratory freq:**<br>Pre-Off *vs*. On, p > 0.1<br>Pre-Off *vs*. Post-Off, p > 0.1<br>On *vs*. post-Off, p > 0.1<br>**Respiratory ampl:**<br>Pre-Off *vs*. On, p > 0.1<br>Pre-Off *vs*. Post-Off, p > 0.1<br>On *vs*. post-Off, p > 0.1	Post-Off, 1.36 bpm<br>**mHR:**<br>Pre-Off, 205.54 bpm<br>On, 205.44 bpm<br>Post-Off, 204.76 bpm<br>**Respiratory freq:**<br>Pre-Off, 97.74 cpm<br>On, 97.60 cpm<br>Post-Off, 97.01 cpm<br>**Respiratory ampl:**<br>Pre-Off, 2.46 (a.u.)<br>On, 2.45 (a.u.)<br>Post-Off, 2.45 (a.u.)	Post-Off, 0.34 bpm<br>**mHR:**<br>Pre-Off, 21.38 bpm<br>On, 21.40 bpm<br>Post-Off, 21.66 bpm<br>**Respiratory freq:**<br>Pre-Off, 11.71 cpm<br>On, 11.66 cpm<br>Post-Off, 11.14 cpm<br>**Respiratory ampl:**<br>Pre-Off, 0.56 (a.u.)<br>On, 0.54 (a.u.)<br>Post-Off, 0.58 (a.u.)
**Ext. Data Fig. 4c**	Delta changes	pre-photoexcitation (Pre-Off) *vs.* Photoexcitation (On)	n/a	n = 5	n/a	**RespHRV**, -0.78 %<br>**mHR**, -0.10 bpm<br>**Respiratory freq**, -0.11 %<br>**Respiratory ampl**, +1.17 %	**RespHRV**, 4.65 %<br>**mHR**, 0.31 bpm<br>**Respiratory freq**, 0.27 %<br>**Respiratory ampl**, 2.07 %
**Ext. Data Fig. 5d**	Absolute values	pre-photoexcitation (Pre-Off) *vs.* Photoexcitation (On) *vs*. Post-photoexcitation (Post-Off)	Repeated Measures one-way ANOVA, Tukey's multiple comparison	n = 6	**RespHRV:**<br>**"preBötC/nA"**<br>Pre-Off *vs*. On, p = 0.0029<br>Pre-Off *vs*. Post-Off, p > 0.1<br>On *vs*. post-Off, p = 0.0033<br>**"DMV"**<br>Pre-Off *vs*. On, p = 0.0043<br>Pre-Off *vs*. Post-Off, p > 0.1<br>On *vs*. post-Off, p = 0.0221<br><br>**mHR:**<br>**"preBötC/nA"**<br>Pre-Off *vs*. On, p = 0.0080<br>Pre-Off *vs*. Post-Off, p > 0.1<br>On *vs*. post-Off, p = 0.0013<br>**"DMV"**<br>Pre-Off *vs*. On, p = 0.0023<br>Pre-Off *vs*. Post-Off, p > 0.1<br>On *vs*. post-Off, p = 0.0011	**RespHRV:**<br>**"preBötC/nA"**<br>Pre-Off, 3.58 bpm<br>On, 5.90 bpm<br>Post-Off, 3.62 bpm<br>**"DMV"**<br>Pre-Off, 4.67 bpm<br>On, 5.78 bpm<br>Post-Off, 4.94 bpm<br><br>**mHR:**<br>**"preBötC/nA"**<br>Pre-Off, 218.67 bpm<br>On, 211.56 bpm<br>Post-Off, 220.89 bpm<br>**"DMV"**<br>Pre-Off, 224.62 bpm<br>On, 220.51 bpm<br>Post-Off, 225.07 bpm	**RespHRV:**<br>**"preBötC/nA"**<br>Pre-Off, 0.86 bpm<br>On, 1.47 bpm<br>Post-Off, 0.95 bpm<br>**"DMV"**<br>Pre-Off, 1.52 bpm<br>On, 1.65 bpm<br>Post-Off, 1.46 bpm<br><br>**mHR:**<br>**"preBötC/nA"**<br>Pre-Off, 4.24 bpm<br>On, 4.74 bpm<br>Post-Off, 5.67 bpm<br>**"DMV"**<br>Pre-Off, 12.68 bpm<br>On, 12.85 bpm<br>Post-Off, 12.90 bpm

					**Respiratory freq:**<br>**“preBötC/nA”**<br>Pre-Off *vs*. On, p > 0.1<br>Pre-Off *vs*. Post-Off, p > 0.1<br>On *vs*. post-Off, p > 0.1<br>**“DMV”**<br>Pre-Off *vs*. On, p > 0.1<br>Pre-Off *vs*. Post-Off, p > 0.1<br>On *vs*. post-Off, p > 0.1<br>**Respiratory ampl:**<br>**“preBötC/nA”**<br>Pre-Off *vs*. On, p > 0.1<br>Pre-Off *vs*. Post-Off, p > 0.1<br>On *vs*. post-Off, p > 0.1<br>**“DMV”**<br>Pre-Off *vs*. On, p = 0.0041<br>Pre-Off *vs*. Post-Off, p > 0.1<br>On *vs*. post-Off, p = 0.0063	**Respiratory freq:**<br>**“preBötC/nA”**<br>Pre-Off, 32.52 cpm<br>On, 32.12 cpm<br>Post-Off, 32.69 cpm<br>**“DMV”**<br>Pre-Off, 26.44 cpm<br>On, 26.14 cpm<br>Post-Off, 26.63 cpm<br>**Respiratory ampl:**<br>**“preBötC/nA”**<br>Pre-Off, 6.28 (a.u.)<br>On, 6.91 (a.u.)<br>Post-Off, 6.83 (a.u.)<br>**“DMV”**<br>Pre-Off, 7.49 (a.u.)<br>On, 8.23 (a.u.)<br>Post-Off, 7.54 (a.u.)	**Respiratory freq:**<br>**“preBötC/nA”**<br>Pre-Off, 4.93 cpm<br>On, 4.65 cpm<br>Post-Off, 4.97 cpm<br>**“DMV”**<br>Pre-Off, 2.12 cpm<br>On, 1.93 cpm<br>Post-Off, 2.11 cpm<br>**Respiratory ampl:**<br>**“preBötC/nA”**<br>Pre-Off, 1.23 (a.u.)<br>On, 1.38 (a.u.)<br>Post-Off, 1.30 (a.u.)<br>**“DMV”**<br>Pre-Off, 1.17 (a.u.)<br>On, 1.31 (a.u.)<br>Post-Off, 1.28 (a.u.)
**Ext. Data Fig. 5e**	Absolute values	pre-photoexcitation (Pre-Off) *vs.* Photoexcitation (On) *vs*. Post-photoexcitation (Post-Off)	Repeated Measures one-way ANOVA, Tukey’s multiple comparison	n = 7	**RespHRV:**<br>**“OT-R antagonist + preBötC/nA”**<br>Pre-Off *vs*. On, p > 0.1<br>Pre-Off *vs*. Post-Off, p > 0.1<br>On *vs*. post-Off, p > 0.1<br>**“OT-R antagonist + DMV”**<br>Pre-Off *vs*. On, p > 0.1<br>Pre-Off *vs*. Post-Off, p > 0.1<br>On *vs*. post-Off, p > 0.1<br><br>**mHR:**<br>**“OT-R antagonist + preBötC/nA”**<br>Pre-Off *vs*. On, p = 0.0082<br>Pre-Off *vs*. Post-Off, p > 0.1<br>On *vs*. post-Off, p = 0.0012<br>**“OT-R antagonist + DMV”**<br>Pre-Off *vs*. On, p = 0.0008<br>Pre-Off *vs*. Post-Off, p > 0.1	**RespHRV:**<br>**“OT-R antagonist + preBötC/nA”**<br>Pre-Off, 4.65 bpm<br>On, 5.00 bpm<br>Post-Off, 4.70 bpm<br>**“OT-R antagonist + DMV”**<br>Pre-Off, 3.48 bpm<br>On, 3.62 bpm<br>Post-Off, 3.45 bpm<br><br>**mHR:**<br>**“OT-R antagonist + preBötC/nA”**<br>Pre-Off, 252.80 bpm<br>On, 248.66 bpm<br>Post-Off, 254.02 bpm<br>**“OT-R antagonist + DMV”**	**RespHRV:**<br>**“OT-R antagonist + preBötC/nA”**<br>Pre-Off, 1.74 bpm<br>On, 1.64 bpm<br>Post-Off, 1.88 bpm<br>**“OT-R antagonist + DMV”**<br>Pre-Off, 0.57 bpm<br>On, 0.62 bpm<br>Post-Off, 0.72 bpm<br><br>**mHR:**<br>**“OT-R antagonist + preBötC/nA”**<br>Pre-Off, 22.29 bpm<br>On, 23.15 bpm<br>Post-Off, 22.14 bpm<br>**“OT-R antagonist + DMV”**

					On *vs*. post-Off, p = 0.0003<br><br><br><br>**Respiratory freq:**<br>**“OT-R antagonist + preBötC/nA”**<br>Pre-Off *vs*. On, p > 0.1<br>Pre-Off *vs*. Post-Off, p > 0.1<br>On *vs*. post-Off, p > 0.1<br>**“OT-R antagonist + DMV”**<br>Pre-Off *vs*. On, p > 0.1<br>Pre-Off *vs*. Post-Off, p > 0.1<br>On *vs*. post-Off, p > 0.1<br><br><br><br><br>**Respiratory ampl:**<br>**“OT-R antagonist + preBötC/nA”**<br>Pre-Off *vs*. On, p > 0.1<br>Pre-Off *vs*. Post-Off, p > 0.1<br>On *vs*. post-Off, p = 0.0278<br>**“OT-R antagonist + DMV”**<br>Pre-Off *vs*. On, p = 0.0041<br>Pre-Off *vs*. Post-Off, p > 0.1<br>On *vs*. post-Off, p = 0.0472	Pre-Off, 266.22 bpm<br>On, 263.92 bpm<br>Post-Off, 266.50 bpm<br><br>**Respiratory freq:**<br>**“OT-R antagonist + preBötC/nA”**<br>Pre-Off, 30.40 cpm<br>On, 30.74 cpm<br>Post-Off, 30.60 cpm<br>**“OT-R antagonist + DMV”**<br>Pre-Off, 30.74 cpm<br>On, 29.95 cpm<br>Post-Off, 30.80 cpm<br><br>**Respiratory ampl:**<br>**“OT-R antagonist + preBötC/nA”**<br>Pre-Off, 6.45 (a.u.)<br>On, 6.57 (a.u.)<br>Post-Off, 6.28 (a.u.)<br>**“OT-R antagonist + DMV”**<br>Pre-Off, 6.48 (a.u.)<br>On, 6.85 (a.u.)<br>Post-Off, 6.28 (a.u.)	Pre-Off, 21.74 bpm<br>On, 21.97 bpm<br>Post-Off, 21.88 bpm<br><br>**Respiratory freq:**<br>**“OT-R antagonist + preBötC/nA”**<br>Pre-Off, 3.64 cpm<br>On, 3.32 cpm<br>Post-Off, 3.74 cpm<br>**“OT-R antagonist + DMV”**<br>Pre-Off, 3.80 cpm<br>On, 3.46 cpm<br>Post-Off, 3.90 cpm<br><br>**Respiratory ampl:**<br>**“OT-R antagonist + preBötC/nA”**<br>Pre-Off, 0.80 (a.u.)<br>On, 0.80 (a.u.)<br>Post-Off, 0.80 (a.u.)<br>**“OT-R antagonist + DMV”**<br>Pre-Off, 0.69 (a.u.)<br>On, 0.74 (a.u.)<br>Post-Off, 0.70 (a.u.)
**Ext. Data Fig. 5f**	Delta changes	Intra-group analysis from Ext. Data Fig. 5d-e: pre-photoexcitation (Pre-Off) *vs*. Photoexcitation (On)	Repeated Measures one-way ANOVA, Tukey’s multiple comparison (test performed on pre-photoexcitation (Pre-Off) *vs*. photoexcitation (On) *vs*. post-	“preBötC/nA” and “DMV” before OT-R antagonist, n = 6; “preBötC/nA” and “DMV” after OT-R antagonist, n = 7	**RespHRV:**<br>before OT-R antagonist, “preBötC/nA”, p = 0.0029<br>“DMV”, p = 0.0043<br>“OT-R antagonist + preBötC/nA”, p > 0.1<br>“OT-R antagonist + DMV”, p > 0.1	**RespHRV:**<br>**“preBötC/nA”**<br>Pre-Off, 3.58 bpm<br>On, 5.90 bpm<br>**“DMV”**<br>Pre-Off, 4.67 bpm<br>On, 5.78 bpm<br>**“OT-R antagonist + preBötC/nA”**<br>Pre-Off, 4.65 bpm<br>On, 5.00 bpm	**RespHRV:**<br>**“preBötC/nA”**<br>Pre-Off, 0.86 bpm<br>On, 1.47 bpm<br>**“DMV”**<br>Pre-Off, 1.52 bpm<br>On, 1.65 bpm<br>**“OT-R antagonist + preBötC/nA”**<br>Pre-Off, 1.74 bpm<br>On, 1.64 bpm

			photoexcitation<br>(Post-Off) data)		 <br><br><br><br>**mHR:**<br>before OT-R antagonist,<br>“preBötC/nA”, p = 0.0080<br>“DMV”, p = 0.0023<br>“OT-R antagonist + preBötC/nA”, p = 0.0082<br>“OT-R antagonist + DMV”, p = 0.0008<br><br><br><br><br><br><br><br><br><br><br>**Respiratory ampl:**<br>before OT-R antagonist,<br>preBötC/nA, p > 0.1<br>DMV, p = 0.0041<br>after OT-R antagonist,<br>preBötC/nA, p > 0.1<br>DMV, p > 0.1	**“OT-R antagonist + DMV”**<br>Pre-Off, 3.48 bpm<br>On, 3.62 bpm<br><br>**mHR:**<br>**“preBötC/nA”**<br>Pre-Off, 218.67 bpm<br>On, 211.56 bpm<br>**“DMV”**<br>Pre-Off, 224.62 bpm<br>On, 220.51 bpm<br>**“OT-R antagonist + preBötC/nA”**<br>Pre-Off, 252.80 bpm<br>On, 248.66 bpm<br>**“OT-R antagonist + DMV”**<br>Pre-Off, 266.22 bpm<br>On, 263.92 bpm<br><br>**Respiratory ampl:**<br>**“preBötC/nA”**<br>Pre-Off, 6.28 (a.u.)<br>On, 6.91 (a.u.)<br>**“DMV”**<br>Pre-Off, 7.49 (a.u.)<br>On, 8.23 (a.u.)<br>**“OT-R antagonist + preBötC/nA”**<br>Pre-Off, 6.45 (a.u.)<br>On, 6.57 (a.u.)<br>**“OT-R antagonist + DMV”**<br>Pre-Off, 6.48 (a.u.)<br>On, 6.85 (a.u.)	**“OT-R antagonist + DMV”**<br>Pre-Off, 0.57 bpm<br>On, 0.62 bpm<br><br>**mHR:**<br>**“preBötC/nA”**<br>Pre-Off, 4.24 bpm<br>On, 4.74 bpm<br>**“DMV”**<br>Pre-Off, 12.68 bpm<br>On, 12.85 bpm<br>**“OT-R antagonist + preBötC/nA”**<br>Pre-Off, 22.29 bpm<br>On, 23.15 bpm<br>**“OT-R antagonist + DMV”**<br>Pre-Off, 21.74 bpm<br>On, 21.97 bpm<br><br>**Respiratory ampl:**<br>**“preBötC/nA”**<br>Pre-Off, 1.23 (a.u.)<br>On, 1.38 (a.u.)<br>**“DMV”**<br>Pre-Off, 1.17 (a.u.)<br>On, 1.31 (a.u.)<br>**“OT-R antagonist + preBötC/nA”**<br>Pre-Off, 0.80 (a.u.)<br>On, 0.80 (a.u.)<br>**“OT-R antagonist + DMV”**<br>Pre-Off, 0.69 (a.u.)<br>On, 0.74 (a.u.)

					**Respiratory freq:**<br>before OT-R antagonist,<br>preBötC/nA, $p > 0.1$<br>DMV, $p > 0.1$<br>after OT-R antagonist,<br>preBötC/nA, $p > 0.1$<br>DMV, $p > 0.1$	**Respiratory freq:**<br>**"preBötC/nA"**<br>Pre-Off, 32.52 cpm<br>On, 32.12 cpm<br>**"DMV"**<br>Pre-Off, 26.44 cpm<br>On, 26.14 cpm<br>**"OT-R antagonist + preBötC/nA"**<br>Pre-Off, 30.40 cpm<br>On, 30.74 cpm<br>**"OT-R antagonist + DMV"**<br>Pre-Off, 30.74 cpm<br>On, 29.95 cpm	**Respiratory freq:**<br>**"preBötC/nA"**<br>Pre-Off, 4.93 cpm<br>On, 4.65 cpm<br>**"DMV"**<br>Pre-Off, 2.12 cpm<br>On, 1.93 cpm<br>**"OT-R antagonist + preBötC/nA"**<br>Pre-Off, 3.64 cpm<br>On, 3.32 cpm<br>**"OT-R antagonist + DMV"**<br>Pre-Off, 3.80 cpm<br>On, 3.46 cpm
**Ext. Data Fig. 5f**	Delta changes	Inter-group analysis: "preBötC/nA" *vs.* "DMV"	Paired t-test	"preBötC/nA" and "DMV" before OT-R antagonist, n = 6;<br>"preBötC/nA" and "DMV" after OT-R antagonist, n = 7	**RespHRV:**<br>Before OT-R antagonist<br>"preBötC/nA" vs. "DMV", $p = 0.0569$<br>After OT-R antagonist<br>"preBötC/nA" vs. "DMV", $p > 0.1$<br><br>**mHR:**<br>Before OT-R antagonist<br>"preBötC/nA" vs. "DMV", $p > 0.1$<br>After OT-R antagonist<br>"preBötC/nA" vs. "DMV", $p > 0.1$<br><br>**Respiratory ampl:**<br>Before OT-R antagonist<br>"preBötC/nA" *vs.* "DMV", $p > 0.1$<br>After OT-R antagonist<br>"preBötC/nA" *vs.* "DMV", $p > 0.1$	**RespHRV:**<br>Before OT-R antagonist,<br>preBötC/nA, +63.16 %<br>DMV, +34.35 %<br>After OT-R antagonist,<br>preBötC/nA, +18.45 %<br>DMV, +4.87 %<br><br>**mHR:**<br>Before OT-R antagonist,<br>preBötC/nA, -7.11 bpm<br>DMV, -4.10 bpm<br>After OT-R antagonist,<br>preBötC/nA, -4.46 bpm<br>DMV, -2.00 bpm<br><br>**Respiratory ampl:**<br>Before OT-R antagonist:<br>preBötC/nA, +10.05 %<br>DMV, +9.46 %<br>After OT-R antagonist:<br>preBötC/nA, +2.25 %<br>DMV, +5.69 %	**RespHRV:**<br>Before OT-R antagonist,<br>preBötC/nA, 6.09 %<br>DMV, 11.31 %<br>After OT-R antagonist,<br>preBötC/nA, 6.25 %<br>DMV, 6.63 %<br><br>**mHR:**<br>Before OT-R antagonist,<br>preBötC/nA, 2.00 bpm<br>DMV, 0.54 bpm<br>After OT-R antagonist,<br>preBötC/nA, 1.18 bpm<br>DMV, 0.45 bpm<br><br>**Respiratory ampl:**<br>Before OT-R antagonist:<br>preBötC/nA, 3.64 %<br>DMV, 1.39 %<br>After OT-R antagonist:<br>preBötC/nA, 1.85 %<br>DMV, 1.77 %

					**Respiratory freq:**<br>Before OT-R antagonist<br>"preBötC/nA" *vs*. "DMV", $p > 0.1$<br>After OT-R antagonist<br>"preBötC/nA" *vs*. "DMV", $p > 0.1$	**Respiratory freq:**<br>Before OT-R antagonist:<br>preBötC/nA, -0.13 %<br>DMV, -0.77 %<br>After OT-R antagonist:<br>preBötC/nA, +2.58 %<br>DMV, -1.82 %	**Respiratory freq:**<br>Before OT-R antagonist:<br>preBötC/nA, 3.18 %<br>DMV, 1.51 %<br>After OT-R antagonist:<br>preBötC/nA, 3.19 %<br>DMV, 2.25 %
**Ext. Data Fig. 6a**	Absolute values and delta changes	Pre-stim RespHRV *vs*. Pre-stim mHR<br><br>Delta RespHRV *vs*. Delta mHR	Pearson correlation analysis, simple linear regression plotted	n = 8	**Pre-stim RespHRV *vs*. Pre-stim mHR:**<br>p = 0.6453, R^2 = 3 %<br><br>**Delta RespHRV *vs*. Delta mHR:**<br>p = 0.4939, R^2 = 8 %	n/a	n/a
**Ext. Data Fig. 6b**	Absolute values and delta changes	Pre-stim RespHRV *vs*. Pre-stim mHR<br><br>Delta RespHRV *vs*. Delta mHR	Pearson correlation analysis, simple linear regression plotted	Cre^+ females, n = 15;<br>Cre^+ males, n = 15	**Pre-stim RespHRV vs. Pre-stim mHR:**<br>p = 0.9230, R^2 = 0.0 %<br><br>**Delta RespHRV vs. Delta mHR:**<br>p = 0.0059, R^2 = 24 %	n/a	n/a
**Ext. Data Fig. 6c**	Absolute values and delta changes	Pre-stim RespHRV *vs*. Pre-stim mHR<br><br>Delta RespHRV *vs*. Delta mHR	Pearson correlation analysis, simple linear regression plotted	Stim preBötC/nA ipsilateral, n = 14;<br>Stim DMV ipsilateral, n = 6;<br>"OT-R antagonist + preBötC/nA ipsilateral", n = 9 ;<br>"OT-R antagonist + preBötC/nA contralateral", n = 6;<br>"OT-R antagonist + DMV ipsilateral",	**Pre-stim RespHRV *vs*. Pre-stim mHR:**<br>p = 0.9105, R^2 = 0.1 %<br><br>**Delta RespHRV *vs*. Delta mHR, stim preBötC/nA ipsi:**<br>p = 0.0279, R^2 = 34 %<br><br>**Delta RespHRV *vs*. Delta mHR, stim DMV ipsi:**<br>p = 0.4910, R^2 = 13 %<br><br>**Delta RespHRV *vs*. Delta mHR, stim preBötC/nA ipsi + OT-R antagonist:**<br>p = 0.2355, R^2 = 17 %<br><br>**Delta RespHRV *vs*. Delta mHR, stim preBötC/nA contra + OT-R antagonist:**<br>p = 0.1216, R^2 = 49 %<br><br>**Delta RespHRV *vs*. Delta mHR, stim DMV ipsi + OT-R antagonist:**	n/a	n/a

				n = 7	p = 0.8304, R^2 = 1 %		
**Ext. Data Fig. 6d**	Absolute values and delta changes	Pre-stim RespHRV *vs*. Pre-stim mHR<br><br>Delta RespHRV *vs*. Delta mHR	Pearson correlation analysis, simple linear regression plotted	n = 10	**Pre-stim RespHRV *vs*. Pre-stim mHR:**<br>p = 0.9517, R^2 = 0.01 %<br><br>**Delta RespHRV *vs*. Delta mHR:**<br>$p < 0.0001$, R^2 = 68 %	n/a	n/a
**Ext. Data Fig. 6e**	Absolute values and delta changes	Pre-TGOT RespHRV *vs*. Pre-TGOT mHR<br><br>Delta RespHRV *vs*. Delta mHR	Pearson correlation analysis, simple linear regression plotted	Pre-TGOT (basal) state, n = 19;<br>After TGOT injection, n= 14	**Pre-TGOT RespHRV *vs*. Pre-TGOT mHR:**<br>p = 0.1196, R^2 = 14 %<br><br>**Delta RespHRV *vs*. Delta mHR:**<br>p = 0.4377, R^2 = 5 %	n/a	n/a
**Ext. Data Fig. 6f**	Absolute values and delta changes	Pre-TGOT RespHRV *vs*. Pre-TGOT mHR<br><br>Delta RespHRV *vs*. Delta mHR	Pearson correlation analysis, simple linear regression plotted	n = 18	**Pre-TGOT RespHRV *vs*. Pre-TGOT mHR:**<br>p = 0.5798, R^2 = 2 %<br><br>**Delta RespHRV *vs*. Delta mHR:**<br>p = 0.8527, R^2 = 0.2 %	n/a	n/a
**3a**	Relative values	Colocalization FG and OT-R (Absolute values in Ext. Data Fig. 7a)	n/a	nA counts, n = 6;<br>DMV count, n = 5	n/a	**nA**<br>FG$^+$ OT-R$^+$, 1.16 %<br>FG$^+$ OT-R$^-$, 98.84 %<br>**nA** **^{Cardiac}**<br>FG$^+$ CGRP$^-$ OT-R$^+$, 1.65 %<br>FG$^+$ CGRP$^-$ OT-R$^-$, 98.35 %<br>**DMV**<br>FG$^+$ OT-R$^+$, 69.35 %<br>FG$^+$ OT-R$^-$, 30.65 %	**nA**<br>FG$^+$ OT-R$^+$, 0.56 %<br>FG$^+$ OT-R$^-$, 0.56 %<br>**nA** **^{Cardiac}**<br>FG$^+$ CGRP$^-$ OT-R$^+$, 0.77 %<br>FG$^+$ CGRP$^-$ OT-R$^-$, 0.77 %<br><br>**DMV**<br>FG$^+$ OT-R$^+$, 4.61 %<br>FG$^+$ OT-R$^-$, 4.61 %
**3b**	Relative values	Colocalization OT-R and FG, NeuN, GS, NK1-R or µO-R (Absolute values in Ext. Data Fig. 7b-c)	n/a	FG, n = 4;<br>NeuN, n = 4;<br>GS, n = 4;<br>NK1-R, n = 4;<br>µO-R, n = 6	n/a	OT-R$^+$ FG$^+$, 5.40 %<br>OT-R$^+$ FG$^-$, 94.60 %<br>OT-R$^+$ NeuN$^+$, 65.96 %<br>OT-R$^+$ NeuN$^-$, 34.04 %<br>OT-R$^+$ GS$^+$, 12.11 %<br>OT-R$^+$ GS$^-$, 87.89 %<br>OT-R$^+$ NK1-R$^+$, 26.76 %<br>OT-R$^+$ NK1-R$^-$, 73.24 %<br>OT-R$^+$ µO-R$^+$, 21.55 %<br>OT-R$^+$ µO-R$^-$, 78.45 %	OT-R$^+$ FG$^+$, 3.92 %<br>OT-R$^+$ FG$^-$, 3.92 %<br>OT-R$^+$ NeuN$^+$, 4.31 %<br>OT-R$^+$ NeuN$^-$, 4.31 %<br>OT-R$^+$ GS$^+$, 2.57 %<br>OT-R$^+$ GS$^-$, 2.57 %<br>OT-R$^+$ NK1-R$^+$, 2.81 %<br>OT-R$^+$ NK1-R$^-$, 2.81 %<br>OT-R$^+$ µO-R$^+$, 1.18 %<br>OT-R$^+$ µO-R$^-$, 1.18 %

3f	Relative values	Colocalization OT-R and Glut or Gly (Absolute values in Ext. Data Fig. 7d)	n/a	n = 4	n/a	OT-R$^+$ Glut$^+$, 9.52 %<br>OT-R$^+$ Gly$^+$, 89.16 %<br>OT-R$^+$ Gly$^-$ Glut$^-$, 1.32 %	OT-R$^+$ Glut$^+$, 2.56 %<br>OT-R$^+$ Gly$^+$, 2.80 %<br>OT-R$^+$ Gly$^-$ Glut$^-$, 0.49 %
Ext. Data Fig. 7a	Absolute and relative values	Colocalization FG and OT-R	n/a	nA counts, n = 6;<br>DMV count, n = 5	n/a	**nA**<br>FG$^+$, 595.67 cells<br>FG$^+$ OT-R$^+$, 7.67 cells<br>FG$^+$ OT-R$^+$/FG$^+$, 1.16 %<br>**nA** ^{Cardiac}<br>FG$^+$ CGRP$^-$, 120.33 cells<br>FG$^+$ CGRP$^-$ OT-R$^+$, 2.00 cells<br>FG$^+$ CGRP$^-$ OT-R$^+$/FG$^+$ CGRP$^-$, 1.65 %<br>**DMV**<br>FG$^+$, 854.40 cells<br>FG$^+$ OT-R$^+$, 587.20 cells<br>FG$^+$ OT-R$^+$/FG$^+$, 69.35 %	**nA**<br>FG$^+$, 43.29 cells<br>FG$^+$ OT-R$^+$, 3.77 cells<br>FG$^+$ OT-R$^+$/FG$^+$, 0.56 %<br>**nA** ^{Cardiac}<br>FG$^+$ CGRP$^-$, 8.97 cells<br>FG$^+$ CGRP$^-$ OT-R$^+$, 0.89 cells<br>FG$^+$ CGRP$^-$ OT-R$^+$/FG$^+$ CGRP$^-$, 0.77 %<br>**DMV**<br>FG$^+$, 155.67 cells<br>FG$^+$ OT-R$^+$, 106.38 cells<br>FG$^+$ OT-R$^+$/FG$^+$, 4.61 %
Ext. Data Fig. 7b	Absolute and relative values	Colocalization OT-R and NeuN, GS, NK1-R or µO-R	n/a	n = 4	n/a	OT-R$^+$, 362.00 cells<br>OT-R$^+$ NeuN$^+$, 239.00 cells<br>OT-R$^+$ GS$^+$, 46.00 cells<br>OT-R$^+$ NeuN$^+$/OT-R$^+$, 65.96 %<br>OT-R$^+$ GS$^+$/OT-R$^+$, 12.11 %	OT-R$^+$, 29.96 cells<br>OT-R$^+$ NeuN$^+$, 24.08 cells<br>OT-R$^+$ GS$^+$, 14.09 cells<br>OT-R$^+$ NeuN$^+$/OT-R$^+$, 4.31 %<br>OT-R$^+$ GS$^+$/OT-R$^+$, 2.57 %
Ext. Data Fig. 7c	Absolute and relative values	Colocalization OT-R and FG, NK1-R or µO-R	n/a	FG, n = 4;<br>NK1-R, n = 4;<br>µO-R, n = 6	n/a	**FG**<br>OT-R$^+$, 353.00 cells<br>OT-R$^+$ FG$^+$, 16.00 cells<br>OT-R$^+$ FG$^+$/OT-R$^+$, 5.40 %<br>**NK1-R**<br>OT-R$^+$, 353.00 cells<br>OT-R$^+$ NK1-R$^+$, 90.00 cells<br>OT-R$^+$ NK1-R$^+$/OT-R$^+$, 26.76 %<br>**µO-R**	**FG**<br>OT-R$^+$, 55.48 cells<br>OT-R$^+$ FG$^+$, 10.71 cells<br>OT-R$^+$ FG$^+$/OT-R$^+$, 3.92 %<br>**NK1-R**<br>OT-R$^+$, 55.48 cells<br>OT-R$^+$ NK1-R$^+$, 5.03 cells<br>OT-R$^+$ NK1-R$^+$/OT-R$^+$, 2.81 %<br>**µO-R**

						OT-R$^+$, 271.33 cells<br>OT-R$^+$ µO-R$^+$, 58.67 cells<br>OT-R$^+$ µO-R$^+$/OT-R$^+$, 21.55 %	OT-R$^+$, 24.25 cells<br>OT-R$^+$ µO-R$^+$, 6.82 cells<br>OT-R$^+$ µO-R$^+$/OT-R$^+$, 1.18 %
**Ext. Data Fig. 7d**	Absolute and relative values	Colocalization OT-R and Glut or Gly	n/a	n = 4	n/a	OT-R$^+$, 156.75 cells<br>OT-R$^+$ Gly$^+$, 140.25 cells<br>OT-R$^+$ Glut$^+$, 14.50 cells<br>OT-R$^+$ Gly$^-$ Glut$^-$, 2.00 cells<br>OT-R$^+$ Gly$^+$/ OT-R$^+$, 89.16 %<br>OT-R$^+$ Glut$^+$/ OT-R$^+$, 9.52 %<br>OT-R$^+$ Gly$^-$ Glut$^-$/ OT-R$^+$, 1.32 %	OT-R$^+$, 6.50 cells<br>OT-R$^+$ Gly$^+$, 9.90 cells<br>OT-R$^+$ Glut$^+$, 3.59 cells<br>OT-R$^+$ Gly$^-$ Glut$^-$, 0.71 cells<br>OT-R$^+$ Gly$^+$/ OT-R$^+$, 2.80 %<br>OT-R$^+$ Glut$^+$/ OT-R$^+$, 2.56 %<br>OT-R$^+$ Gly$^-$ Glut$^-$/ OT-R$^+$, 0.49 %
**Ext. Data Fig. 7e-g**	Absolute and relative values	Colocalization OT-R and GAD67 or GlyT2	n/a	n = 4	n/a	**GAD67**<br>OT-R$^+$, 207.50 cells<br>OT-R$^+$ GAD67$^+$, 103.00 cells<br>OT-R$^+$ GAD67$^+$/OT-R$^+$, 50.15 %<br>**GlyT2**<br>OT-R$^+$, 271.50 cells<br>OT-R$^+$ GlyT2$^+$, 114.00 cells<br>OT-R$^+$ GlyT2$^+$/OT-R$^+$, 41.64 %	**GAD67**<br>OT-R$^+$, 10.37 cells<br>OT-R$^+$ GAD67$^+$, 11.00 cells<br>OT-R$^+$ GAD67$^+$/OT-R$^+$, 3.01 %<br>**GlyT2**<br>OT-R$^+$, 10.56 cells<br>OT-R$^+$ GlyT2$^+$, 13.04 cells<br>OT-R$^+$ GlyT2$^+$/OT-R$^+$, 3.32 %
**Ext. Data Fig. 7j**	Absolute values	pre-photoexcitation (Pre-Off) *vs.* Photoexcitation (On) vs. Post-photoexcitation (Post-Off)	Repeated Measures one-way ANOVA, Tukey's multiple	Cre$^+$ no treatment, n = 8; Cre$^+$ atropine, n = 6; Cre$^-$ no treatment, n = 4	**RespHRV**:<br>**"Cre$^+$ no treatment"**<br>Pre-Off vs. On, p = 0.0013<br>Pre-Off vs. Post-Off, p > 0.1<br>On vs. post-Off, p = 0.0305<br>**"Cre$^+$ atropine"**<br>Pre-Off vs. On, p > 0.1<br>Pre-Off vs. Post-Off, p > 0.1<br>On vs. post-Off, p > 0.1<br>**"Cre$^-$ no treatment"**<br>Pre-Off vs. On, p > 0.1	**RespHRV**:<br>**"Cre$^+$ no treatment"**<br>Pre-Off, 2.38 bpm<br>On, 1.06 bpm<br>Post-Off, 1.90 bpm<br>**"Cre$^+$ atropine"**<br>Pre-Off, 1.72 bpm<br>On, 1.83 bpm<br>Post-Off, 1.65 bpm<br>**"Cre$^-$ no treatment"**<br>Pre-Off, 2.93 bpm	**RespHRV**:<br>**"Cre$^+$ no treatment"**<br>Pre-Off, 0.56 bpm<br>On, 0.26 bpm<br>Post-Off, 0.52 bpm<br>**"Cre$^+$ atropine"**<br>Pre-Off, 0.38 bpm<br>On, 0.39 bpm<br>Post-Off, 0.35 bpm<br>**"Cre$^-$ no treatment"**<br>Pre-Off, 0.65 bpm

					Pre-Off vs. Post-Off, p > 0.1<br>On vs. post-Off, p > 0.1<br><br>**mHR:**<br>**“Cre$^+$ no treatment”**<br>Pre-Off vs. On, p = 0.0061<br>Pre-Off vs. Post-Off, p > 0.1<br>On vs. post-Off, p = 0.0093<br>**“Cre$^+$ atropine”**<br>Pre-Off vs. On, p > 0.1<br>Pre-Off vs. Post-Off, p > 0.1<br>On vs. post-Off, p > 0.1<br>**“Cre$^-$ no treatment”**<br>Pre-Off vs. On, p > 0.1<br>Pre-Off vs. Post-Off, p > 0.1<br>On vs. post-Off, p > 0.1<br><br>**Respiratory freq:**<br>**“Cre$^+$ no treatment”**<br>Pre-Off vs. On, p = 0.0178<br>Pre-Off vs. Post-Off, p > 0.1<br>On vs. post-Off, p = 0.0030<br>**“Cre$^+$ atropine”**<br>Pre-Off vs. On, p = 0.0015<br>Pre-Off vs. Post-Off, p > 0.1<br>On vs. post-Off, p = 0.0013<br>**“Cre$^-$ no treatment”**<br>Pre-Off vs. On, p > 0.1<br>Pre-Off vs. Post-Off, p > 0.1<br>On vs. post-Off, p > 0.1<br><br>**Respiratory ampl:**<br>**“Cre$^+$ no treatment”**<br>Pre-Off vs. On, p > 0.1<br>Pre-Off vs. Post-Off, p > 0.1<br>On vs. post-Off, p > 0.1<br>**“Cre$^+$ atropine”**<br>Pre-Off vs. On, p > 0.1	On, 3.03 bpm<br>Post-Off, 2.93 bpm<br><br>**mHR:**<br>**“Cre$^+$ no treatment”**<br>Pre-Off, 246.59 bpm<br>On, 258.22 bpm<br>Post-Off, 247.29 bpm<br>**“Cre$^+$ atropine”**<br>Pre-Off, 256.88 bpm<br>On, 258.28 bpm<br>Post-Off, 259.36 bpm<br>**“Cre$^-$ no treatment”**<br>Pre-Off, 255.57 bpm<br>On, 255.64 bpm<br>Post-Off, 255.96 bpm<br><br>**Respiratory freq:**<br>**“Cre$^+$ no treatment”**<br>Pre-Off, 24.28 cpm<br>On, 30.16 cpm<br>Post-Off, 22.57 cpm<br>**“Cre$^+$ atropine”**<br>Pre-Off, 23.76 cpm<br>On, 28.70 cpm<br>Post-Off, 23.68 cpm<br>**“Cre$^-$ no treatment”**<br>Pre-Off, 23.16 cpm<br>On, 22.91 cpm<br>Post-Off, 22.97 cpm<br><br>**Respiratory ampl:**<br>**“Cre$^+$ no treatment”**<br>Pre-Off, 3.86 (a.u.)<br>On, 3.87 (a.u.)<br>Post-Off, 3.97 (a.u.)<br>**“Cre$^+$ atropine”**<br>Pre-Off, 3.65 (a.u.)	On, 0.65 bpm<br>Post-Off, 0.67 bpm<br><br>**mHR:**<br>**“Cre$^+$ no treatment”**<br>Pre-Off, 22.73 bpm<br>On, 22.74 bpm<br>Post-Off, 22.42 bpm<br>**“Cre$^+$ atropine”**<br>Pre-Off, 16.28 bpm<br>On, 15.92 bpm<br>Post-Off, 15.41 bpm<br>**“Cre$^-$ no treatment”**<br>Pre-Off, 8.79 bpm<br>On, 8.74 bpm<br>Post-Off, 8.66 bpm<br><br>**Respiratory freq:**<br>**“Cre$^+$ no treatment”**<br>Pre-Off, 1.76 cpm<br>On, 3.13 cpm<br>Post-Off, 1.76 cpm<br>**“Cre$^+$ atropine”**<br>Pre-Off, 1.46 cpm<br>On, 1.43 cpm<br>Post-Off, 1.75 cpm<br>**“Cre$^-$ no treatment”**<br>Pre-Off, 1.75 cpm<br>On, 1.93 cpm<br>Post-Off, 1.78 cpm<br><br>**Respiratory ampl:**<br>**“Cre$^+$ no treatment”**<br>Pre-Off, 0.62 (a.u.)<br>On, 0.62 (a.u.)<br>Post-Off, 0.70 (a.u.)<br>**“Cre$^+$ atropine”**<br>Pre-Off, 0.56 (a.u.)

					Pre-Off vs. Post-Off, p > 0.1<br>On vs. post-Off, p > 0.1<br>**“Cre$^-$ no treatment”**<br>Pre-Off vs. On, p > 0.1<br>Pre-Off vs. Post-Off, p > 0.1<br>On vs. post-Off, p > 0.1	On, 3.62 (a.u.)<br>Post-Off, 3.59 (a.u.)<br>**“Cre$^-$ no treatment”**<br>Pre-Off, 4.46 (a.u.)<br>On, 4.49 (a.u.)<br>Post-Off, 4.70 (a.u.)	On, 0.52 (a.u.)<br>Post-Off, 0.49 (a.u.)<br>**“Cre$^-$ no treatment”**<br>Pre-Off, 0.33 (a.u.)<br>On, 0.33 (a.u.)<br>Post-Off, 0.42 (a.u.)
**Ext. Data Fig. 7k**	Absolute values	pre-photoinhibition (Pre-Off) *vs.* Photoinhibition (On) vs. Post-photoinhibition (Post-Off)	Repeated Measures one-way ANOVA, Tukey’s multiple	Cre$^+$ no treatment, n = 10; Cre$^+$ atropine, n = 6; Cre$^-$, n = 4	**RespHRV**:<br>**“Cre$^+$ no treatment”**<br>Pre-Off *vs.* On, p = 0.0046<br>Pre-Off vs. Post-Off, p > 0.1<br>On *vs.* post-Off, p = 0.0038<br>**“Cre$^+$ atropine”**<br>Pre-Off vs. On, p > 0.1<br>Pre-Off vs. Post-Off, p > 0.1<br>On vs. post-Off, p > 0.1<br>**“Cre$^-$ no treatment”**<br>Pre-Off *vs.* On, p > 0.1<br>Pre-Off *vs.* Post-Off, p > 0.1<br>On *vs.* post-Off, p > 0.1<br><br>**mHR:**<br>**“Cre$^+$ no treatment”**<br>Pre-Off *vs.* On, p = 0.0002<br>Pre-Off *vs.* Post-Off, p > 0.1<br>On *vs.* post-Off, p = 0.0003<br>**“Cre$^+$ atropine”**<br>Pre-Off *vs.* On, p > 0.1<br>Pre-Off *vs.* Post-Off, p > 0.1<br>On *vs.* post-Off, p > 0.1<br>**“Cre$^-$ no treatment”**<br>Pre-Off *vs.* On, p > 0.1<br>Pre-Off *vs.* Post-Off, p > 0.1<br>On *vs.* post-Off, p > 0.1<br><br>**Respiratory freq:**<br>**“Cre$^+$ no treatment”**<br>Pre-Off *vs.* On, p < 0.0001<br>Pre-Off *vs.* Post-Off, p > 0.1	**RespHRV**:<br>**“Cre$^+$ no treatment”**<br>Pre-Off, 2.32 bpm<br>On, 3.80 bpm<br>Post-Off, 2.29 bpm<br>**“Cre$^+$ atropine”**<br>Pre-Off, 1.61 bpm<br>On, 1.61 bpm<br>Post-Off, 1.56 bpm<br>**“Cre$^-$ no treatment”**<br>Pre-Off, 3.37 bpm<br>On, 3.44 bpm<br>Post-Off, 3.31 bpm<br><br>**mHR:**<br>**“Cre$^+$ no treatment”**<br>Pre-Off, 259.95 bpm<br>On, 252.46 bpm<br>Post-Off, 259.56 bpm<br>**“Cre$^+$ atropine”**<br>Pre-Off, 262.15 bpm<br>On, 262.50 bpm<br>Post-Off, 262.44 bpm<br>**“Cre$^-$ no treatment”**<br>Pre-Off, 245.69 bpm<br>On, 246.08 bpm<br>Post-Off, 246.17 bpm<br><br>**Respiratory freq:**<br>**“Cre$^+$ no treatment”**<br>Pre-Off, 23.34 cpm<br>On, 20.06 cpm	**RespHRV**:<br>**“Cre$^+$ no treatment”**<br>Pre-Off, 0.43 bpm<br>On, 0.75 bpm<br>Post-Off, 0.49 bpm<br>**“Cre$^+$ atropine”**<br>Pre-Off, 0.30 bpm<br>On, 0.32 bpm<br>Post-Off, 0.33 bpm<br>**“Cre$^-$ no treatment”**<br>Pre-Off, 0.60 bpm<br>On, 0.62 bpm<br>Post-Off, 0.69 bpm<br><br>**mHR:**<br>**“Cre$^+$ no treatment”**<br>Pre-Off, 20.89 bpm<br>On, 21.41 bpm<br>Post-Off, 20.98 bpm<br>**“Cre$^+$ atropine”**<br>Pre-Off, 15.76 bpm<br>On, 15.94 bpm<br>Post-Off, 15.98 bpm<br>**“Cre$^-$ no treatment”**<br>Pre-Off, 6.89 bpm<br>On, 6.88 bpm<br>Post-Off, 6.71 bpm<br><br>**Respiratory freq:**<br>**“Cre$^+$ no treatment”**<br>Pre-Off, 2.02 cpm<br>On, 1.85 cpm

					On *vs*. post-Off, p = 0.0001<br>**“Cre$^+$ atropine”**<br>Pre-Off *vs*. On, p = 0.0062<br>Pre-Off *vs*. Post-Off, p > 0.1<br>On *vs*. post-Off, p = 0.0016<br>**“Cre$^-$ no treatment”**<br>Pre-Off *vs*. On, p > 0.1<br>Pre-Off *vs*. Post-Off, p > 0.1<br>On *vs*. post-Off, p > 0.1<br><br>**Respiratory ampl:**<br>**“Cre$^+$ no treatment”**<br>Pre-Off *vs*. On, p > 0.1<br>Pre-Off *vs*. Post-Off, p > 0.1<br>On *vs*. post-Off, p > 0.1<br>**“Cre$^+$ atropine”**<br>Pre-Off *vs*. On, p > 0.1<br>Pre-Off *vs*. Post-Off, p > 0.1<br>On *vs*. post-Off, p > 0.1<br>**“Cre$^-$ no treatment”**<br>Pre-Off *vs*. On, p > 0.1<br>Pre-Off *vs*. Post-Off, p > 0.1<br>On *vs*. post-Off, p > 0.1	Post-Off, 22.96 cpm<br>**“Cre$^+$ atropine”**<br>Pre-Off, 24.07 cpm<br>On, 22.64 cpm<br>Post-Off, 24.38 cpm<br>**“Cre$^-$ no treatment”**<br>Pre-Off, 27.27 cpm<br>On, 27.51 cpm<br>Post-Off, 26.82 cpm<br><br>**Respiratory ampl:**<br>**“Cre$^+$ no treatment”**<br>Pre-Off, 4.13 (a.u.)<br>On, 4.24 (a.u.)<br>Post-Off, 4.04 (a.u.)<br>**“Cre$^+$ atropine”**<br>Pre-Off, 3.65 (a.u.)<br>On, 3.76 (a.u.)<br>Post-Off, 3.72 (a.u.)<br>**“Cre$^-$ no treatment”**<br>Pre-Off, 4.22 (a.u.)<br>On, 4.13 (a.u.)<br>Post-Off, 4.15 (a.u.)	Post-Off, 2.19 cpm<br>**“Cre$^+$ atropine”**<br>Pre-Off, 1.86 cpm<br>On, 1.77 cpm<br>Post-Off, 1.78 cpm<br>**“Cre$^-$ no treatment”**<br>Pre-Off, 3.78 cpm<br>On, 3.73 cpm<br>Post-Off, 3.52 cpm<br><br>**Respiratory ampl:**<br>**“Cre$^+$ no treatment”**<br>Pre-Off, 0.47 (a.u.)<br>On, 0.53 (a.u.)<br>Post-Off, 0.48 (a.u.)<br>**“Cre$^+$ atropine”**<br>Pre-Off, 0.55 (a.u.)<br>On, 0.56 (a.u.)<br>Post-Off, 0.56 (a.u.)<br>**“Cre$^-$ no treatment”**<br>Pre-Off, 0.43 (a.u.)<br>On, 0.47 (a.u.)<br>Post-Off, 0.36 (a.u.)
**Ext. Data Fig. 7l**	Absolute values	BCHE$^+$ *vs*. Calb$^+$	n/a	n = 4	n/a	BCHE$^+$, 12.75 cells<br>Calb$^+$, 4.25 cells	BCHE$^+$, 3.82 cells<br>Calb$^+$, 1.44 cells
**4c**	Delta changes	Intra-group analysis from Ext. Data Fig. 7j: pre-photoexcitation (Pre-Off) *vs*. Photoexcitation (On)	Repeated Measures one-way ANOVA, Tukey’s multiple comparison (test performed on pre-photoexcitation (Pre-Off) *vs*. photoexcitation (On) *vs*. post-	Cre$^+$ no treatment, n = 8;<br>Cre$^+$ atropine, n = 6;<br>Cre$^-$ no treatment, n = 4	**RespHRV**:<br>“Cre$^+$ no treatment”, Pre-Off *vs*. On, p = 0.0013<br>“Cre$^+$ atropine”, Pre-Off *vs*. On, p > 0.1<br>“Cre$^-$ no treatment”, Pre-Off *vs*. On, p > 0.1	**RespHRV**:<br>**“Cre$^+$ no treatment”**<br>Pre-Off, 2.38 bpm<br>On, 1.06 bpm<br>**“Cre$^+$ atropine”**<br>Pre-Off, 1.72 bpm<br>On, 1.83 bpm<br>**“Cre$^-$ no treatment”**<br>Pre-Off, 2.93 bpm<br>On, 3.03 bpm	**RespHRV**:<br>**“Cre$^+$ no treatment”**<br>Pre-Off, 0.56 bpm<br>On, 0.26 bpm<br>**“Cre$^+$ atropine”**<br>Pre-Off, 0.38 bpm<br>On, 0.39 bpm<br>**“Cre$^-$ no treatment”**<br>Pre-Off, 0.65 bpm<br>On, 0.65 bpm

			photoexcitation (Post-Off) data)		**mHR:**<br>"Cre$^+$ no treatment", Pre-Off *vs*. On, p = 0.0061<br>"Cre$^+$ atropine", Pre-Off *vs*. On, p > 0.1<br>"Cre$^-$ no treatment", Pre-Off *vs*. On, p > 0.1<br><br>**Respiratory freq:**<br>"Cre$^+$ no treatment", Pre-Off *vs*. On, p = 0.0178<br>"Cre$^+$ atropine", Pre-Off *vs*. On, p = 0.0015<br>"Cre$^-$ no treatment", Pre-Off *vs*. On, p > 0.1<br><br>**Respiratory ampl:**<br>"Cre$^+$ no treatment", Pre-Off *vs*. On, p > 0.1<br>"Cre$^+$ atropine", Pre-Off *vs*. On, p > 0.1<br>"Cre$^-$ no treatment", Pre-Off *vs*. On, p > 0.1	**mHR:**<br>**"Cre$^+$ no treatment"**<br>Pre-Off, 246.59 bpm<br>On, 258.22 bpm<br>**"Cre$^+$ atropine"**<br>Pre-Off, 256.88 bpm<br>On, 258.28 bpm<br>**"Cre$^-$ no treatment"**<br>Pre-Off, 255.57 bpm<br>On, 255.64 bpm<br><br>**Respiratory freq:**<br>**"Cre$^+$ no treatment"**<br>Pre-Off, 24.28 cpm<br>On, 30.16 cpm<br>**"Cre$^+$ atropine"**<br>Pre-Off, 23.76 cpm<br>On, 28.70 cpm<br>**"Cre$^-$ no treatment"**<br>Pre-Off, 23.16 cpm<br>On, 22.91 cpm<br><br>**Respiratory ampl:**<br>**"Cre$^+$ no treatment"**<br>Pre-Off, 3.86 (a.u.)<br>On, 3.87 (a.u.)<br>**"Cre$^+$ atropine"**<br>Pre-Off, 3.65 (a.u.)<br>On, 3.62 (a.u.)<br>**"Cre$^-$ no treatment"**<br>Pre-Off, 4.46 (a.u.)<br>On, 4.49 (a.u.)	**mHR:**<br>**"Cre$^+$ no treatment"**<br>Pre-Off, 22.73 bpm<br>On, 22.74 bpm<br>**"Cre$^+$ atropine"**<br>Pre-Off, 16.28 bpm<br>On, 15.92 bpm<br>**"Cre$^-$ no treatment"**<br>Pre-Off, 8.79 bpm<br>On, 8.74 bpm<br><br>**Respiratory freq:**<br>**"Cre$^+$ no treatment"**<br>Pre-Off, 1.76 cpm<br>On, 3.13 cpm<br>**"Cre$^+$ atropine"**<br>Pre-Off, 1.46 cpm<br>On, 1.43 cpm<br>**"Cre$^-$ no treatment"**<br>Pre-Off, 1.75 cpm<br>On, 1.93 cpm<br><br>**Respiratory ampl:**<br>**"Cre$^+$ no treatment"**<br>Pre-Off, 0.62 (a.u.)<br>On, 0.62 (a.u.)<br>**"Cre$^+$ atropine"**<br>Pre-Off, 0.56 (a.u.)<br>On, 0.52 (a.u.)<br>**"Cre$^-$ no treatment"**<br>Pre-Off, 0.33 (a.u.)<br>On, 0.33 (a.u.)
**4c**	Delta changes	Intra-group analysis: "Cre$^+$ no treatment" *vs*. "Cre$^+$ atropine"	Paired t-test or Wilcoxon matched-pairs signed rank test	Cre$^+$ no treatment, n = 8;<br>Cre$^+$ atropine, n = 6	**RespHRV**, p = 0.0106	**RespHRV:**<br>Cre$^+$ no treatment, -49.94 %<br>Cre$^+$ atropine, +6.57 %	**RespHRV:**<br>Cre$^+$ no treatment, 6.63 %<br>Cre$^+$ atropine, 6.97 %

					**mHR**, p = 0.0386<br><br><br><br><br>**Respiratory freq**, p > 0.1<br><br><br><br>**Respiratory ampl**, p > 0.1	**mHR:**<br>Cre$^+$ no treatment, +12.71 bpm<br>Cre$^+$ atropine, +0.52 bpm<br>**Respiratory freq:**<br>Cre$^+$ no treatment, +23.22 %<br>Cre$^+$ atropine, +21.97 %<br>**Respiratory ampl:**<br>Cre$^+$ no treatment, +0.94 %<br>Cre$^+$ atropine, -0.06 %	**mHR:**<br>Cre$^+$ no treatment, 4.52 bpm<br>Cre$^+$ atropine, 0.17 bpm<br>**Respiratory freq:**<br>Cre$^+$ no treatment, 7.31 %<br>Cre$^+$ atropine, 5.82 %<br>**Respiratory ampl:**<br>Cre$^+$ no treatment, 4.00 %<br>Cre$^+$ atropine, 1.59 %
**4c**	Delta changes	Inter-group analysis: "Cre$^+$ no treatment" *vs*. "Cre$^+$ atropine" *vs*. "Cre$^-$ no treatment"	Kruskal-Wallis test, Dunn's multiple comparison	Cre$^+$ no treatment, n = 8;<br>Cre$^+$ atropine, n = 6;<br>Cre$^-$ no treatment, n = 4	**RespHRV:**<br>"Cre$^+$ no treatment" *vs*. "Cre$^-$ no treatment", p = 0.0003<br>"Cre$^+$ atropine" *vs*. "Cre$^-$ no treatment", p > 0.1<br><br>**mHR:**<br>"Cre$^+$ no treatment" *vs*. "Cre$^-$ no treatment", p = 0.0040<br>"Cre$^+$ atropine" *vs*. "Cre$^-$ no treatment", p > 0.1<br><br><br>**Respiratory freq:**<br>"Cre$^+$ no treatment" *vs*. "Cre$^-$ no treatment", p = 0.0280<br>"Cre$^+$ atropine" *vs*. "Cre$^-$ no treatment", p > 0.1<br><br>**Respiratory ampl:**<br>"Cre$^+$ no treatment" *vs*. "Cre$^-$ no treatment", p > 0.1<br>"Cre$^+$ atropine" *vs*. "Cre$^-$ no treatment", p > 0.1	**RespHRV:**<br>Cre$^+$ no treatment, -49.94 %<br>Cre$^+$ atropine, +6.57 %<br>Cre$^-$ no treatment, +4.05 %<br>**mHR:**<br>Cre$^+$ no treatment, +12.71 bpm<br>Cre$^+$ atropine, +0.52 bpm<br>Cre$^-$ no treatment, +0.07 bpm<br>**Respiratory freq:**<br>Cre$^+$ no treatment, +23.22 %<br>Cre$^+$ atropine, +21.97 %<br>Cre$^-$ no treatment, -1.29 %<br>**Respiratory ampl:**<br>Cre$^+$ no treatment, +0.94 %<br>Cre$^+$ atropine, -0.06 %<br>Cre$^-$ no treatment, +0.76 %	**RespHRV:**<br>Cre$^+$ no treatment, 6.63 %<br>Cre$^+$ atropine, 6.97 %<br>Cre$^-$ no treatment, 3.03 %<br>**mHR:**<br>Cre$^+$ no treatment, 4.52 bpm<br>Cre$^+$ atropine, 0.17 bpm<br>Cre$^-$ no treatment, 0.07 bpm<br>**Respiratory freq:**<br>Cre$^+$ no treatment, 7.31 %<br>Cre$^+$ atropine, 5.82 %<br>Cre$^-$ no treatment, 1.36 %<br>**Respiratory ampl:**<br>Cre$^+$ no treatment, 4.00 %<br>Cre$^+$ atropine, 1.59 %<br>Cre$^-$ no treatment, 1.26 %

**4d**	Delta changes	Intra-group analysis from Ext. Data Fig. 7k: pre-photoinhibition (Pre-Off) *vs.* Photoinhibition (On)	Repeated Measures one-way ANOVA, Tukey's multiple comparison or Friedman test, Dunn's multiple comparison (Cre$^-$ RespHRV photoinhibition) (test performed on pre-photoexcitation (Pre-Off) *vs*. photoexcitation (On) *vs*. post-photoexcitation (Post-Off) data)	Cre$^+$ no treatment, n = 10;<br>Cre$^+$ atropine, n = 6;<br>Cre$^-$ no treatment, n = 4	**RespHRV**:<br>"Cre$^+$ no treatment", Pre-Off *vs*. On, p = 0.0046<br>"Cre$^+$ atropine", Pre-Off *vs*. On, p > 0.1<br>"Cre$^-$ no treatment", Pre-Off *vs*. On, p > 0.1<br><br>**mHR:**<br>"Cre$^+$ no treatment", Pre-Off *vs*. On, p = 0.0002<br>"Cre$^+$ atropine", Pre-Off *vs*. On, p > 0.1<br>"Cre$^-$ no treatment", Pre-Off *vs*. On, p > 0.1<br><br>**Respiratory freq:**<br>"Cre$^+$ no treatment", Pre-Off *vs*. On, p < 0.0001<br>"Cre$^+$ atropine", Pre-Off *vs*. On, p = 0.0062<br>"Cre$^-$ no treatment", Pre-Off *vs*. On, p > 0.1<br><br>**Respiratory ampl:**<br>"Cre$^+$ no treatment", Pre-Off *vs*. On, p > 0.1<br>"Cre$^+$ atropine", Pre-Off *vs*. On, p > 0.1<br>"Cre$^-$ no treatment", Pre-Off *vs*. On, p > 0.1	**RespHRV**:<br>**"Cre$^+$ no treatment"**<br>Pre-Off, 2.32 bpm<br>On, 3.80 bpm<br>**"Cre$^+$ atropine"**<br>Pre-Off, 1.61 bpm<br>On, 1.61 bpm<br>**"Cre$^-$ no treatment"**<br>Pre-Off, 3.37 bpm<br>On, 3.44 bpm<br>**mHR:**<br>**"Cre$^+$ no treatment"**<br>Pre-Off, 259.95 bpm<br>On, 252.46 bpm<br>**"Cre$^+$ atropine"**<br>Pre-Off, 262.15 bpm<br>On, 262.50 bpm<br>**"Cre$^-$ no treatment"**<br>Pre-Off, 245.69 bpm<br>On, 246.08 bpm<br><br>**Respiratory freq:**<br>**"Cre$^+$ no treatment"**<br>Pre-Off, 23.34 cpm<br>On, 20.06 cpm<br>**"Cre$^+$ atropine"**<br>Pre-Off, 24.07 cpm<br>On, 22.64 cpm<br>**"Cre$^-$ no treatment"**<br>Pre-Off, 27.27 cpm<br>On, 27.51 cpm<br><br>**Respiratory ampl:**<br>**"Cre$^+$ no treatment"**<br>Pre-Off, 4.13 (a.u.)<br>On, 4.24 (a.u.)<br>**"Cre$^+$ atropine"**<br>Pre-Off, 3.65 (a.u.)	**RespHRV**:<br>**"Cre$^+$ no treatment"**<br>Pre-Off, 0.43 bpm<br>On, 0.75 bpm<br>**"Cre$^+$ atropine"**<br>Pre-Off, 0.30 bpm<br>On, 0.32 bpm<br>**"Cre$^-$ no treatment"**<br>Pre-Off, 0.60 bpm<br>On, 0.62 bpm<br>**mHR:**<br>**"Cre$^+$ no treatment"**<br>Pre-Off, 20.89 bpm<br>On, 21.41 bpm<br>**"Cre$^+$ atropine"**<br>Pre-Off, 15.76 bpm<br>On, 15.94 bpm<br>**"Cre$^-$ no treatment"**<br>Pre-Off, 6.89 bpm<br>On, 6.88 bpm<br><br>**Respiratory freq:**<br>**"Cre$^+$ no treatment"**<br>Pre-Off, 2.02 cpm<br>On, 1.85 cpm<br>**"Cre$^+$ atropine"**<br>Pre-Off, 1.86 cpm<br>On, 1.77 cpm<br>**"Cre$^-$ no treatment"**<br>Pre-Off, 3.78 cpm<br>On, 3.73 cpm<br><br>**Respiratory ampl:**<br>**"Cre$^+$ no treatment"**<br>Pre-Off, 0.47 (a.u.)<br>On, 0.53 (a.u.)<br>**"Cre$^+$ atropine"**<br>Pre-Off, 0.55 (a.u.)

						On, 3.76 (a.u.)<br>**“Cre$^-$ no treatment”**<br>Pre-Off, 4.22 (a.u.)<br>On, 4.13 (a.u.)	On, 0.56 (a.u.)<br>**“Cre$^-$ no treatment”**<br>Pre-Off, 0.43 (a.u.)<br>On, 0.47 (a.u.)
**4d**	Delta changes	Intra-group analysis: “Cre$^+$ no treatment” *vs*. “Cre$^+$ atropine”	Paired t-test or Wilcoxon matched-pairs signed rank test	Cre$^+$ no treatment, n = 10;<br>Cre$^+$ atropine, n = 6	**RespHRV**, p = 0.0158<br><br>**mHR**, p = 0.0147<br><br>**Respiratory freq**, p = 0.0174<br><br>**Respiratory ampl**, p > 0.1	**RespHRV:**<br>Cre$^+$ no treatment, +77.16 %<br>Cre$^+$ atropine, +0.54 %<br>**mHR:**<br>Cre$^+$ no treatment, -7.49 bpm<br>Cre$^+$ atropine, +0.36 bpm<br>**Respiratory freq:**<br>Cre$^+$ no treatment, -14.32 %<br>Cre$^+$ atropine, -5.86 %<br>**Respiratory ampl:**<br>Cre$^+$ no treatment, +2.82 %<br>Cre$^+$ atropine, +3.17 %	**RespHRV:**<br>Cre$^+$ no treatment, 18.49 %<br>Cre$^+$ atropine, 4.08 %<br>**mHR:**<br>Cre$^+$ no treatment, 1.78 bpm<br>Cre$^+$ atropine, 0.25 bpm<br>**Respiratory freq:**<br>Cre$^+$ no treatment, 1.83 %<br>Cre$^+$ atropine, 1.75 %<br>**Respiratory ampl:**<br>Cre$^+$ no treatment, 2.43 %<br>Cre$^+$ atropine, 1.27 %
**4d**	Delta changes	Inter-group analysis: “Cre$^+$ no treatment” *vs*. “Cre$^+$ atropine” *vs*. “Cre$^-$ no treatment”	Kruskal-Wallis test, Dunn’s multiple comparison	Cre$^+$ no treatment, n = 10;<br>Cre$^+$ atropine, n = 6;<br>Cre$^-$, n = 4	**RespHRV:**<br>“Cre$^+$ no treatment” vs. “Cre$^-$ no treatment”, p = 0.0020<br>“Cre$^+$ atropine” vs. “Cre$^-$ no treatment”, p > 0.1<br><br>**mHR:**<br>“Cre$^+$ no treatment” vs. “Cre$^-$ no treatment”, p = 0.0020<br>“Cre$^+$ atropine” vs. “Cre$^-$ no treatment”, p > 0.1<br><br>**Respiratory freq:**<br>“Cre$^+$ no treatment” vs. “Cre$^-$ no treatment”, p = 0.0036	**RespHRV:**<br>Cre$^+$ no treatment, +77.16 %<br>Cre$^+$ atropine, +0.54 %<br>Cre$^-$ no treatment, +2.14 %<br>**mHR:**<br>Cre$^+$ no treatment, -7.49 bpm<br>Cre$^+$ atropine, +0.36 bpm<br>Cre$^-$ no treatment, +0.39 bpm<br>**Respiratory freq:**<br>Cre$^+$ no treatment, -14.32 %<br>Cre$^+$ atropine, -5.86 %	**RespHRV:**<br>Cre$^+$ no treatment, 18.49 %<br>Cre$^+$ atropine, 4.08 %<br>Cre$^-$ no treatment, 3.49 %<br>**mHR:**<br>Cre$^+$ no treatment, 1.78 bpm<br>Cre$^+$ atropine, 0.25 bpm<br>Cre$^-$ no treatment, 0.20 bpm<br>**Respiratory freq:**<br>Cre$^+$ no treatment, 1.83 %<br>Cre$^+$ atropine, 1.75 %

					“Cre$^+$ atropine” vs. “Cre$^-$ no treatment”, p > 0.1<br>**Respiratory ampl:**<br>“Cre$^+$ no treatment” vs. “Cre$^-$ no treatment”, p > 0.1<br>“Cre$^+$ atropine” vs. “Cre$^-$ no treatment”, p > 0.1	Cre$^-$ no treatment, +0.99 %<br>**Respiratory ampl:**<br>Cre$^+$ no treatment, +2.82 %<br>Cre$^+$ atropine, +3.17 %<br>Cre$^-$ no treatment, -2.28 %	Cre$^-$ no treatment, 1.37 %<br>**Respiratory ampl:**<br>Cre$^+$ no treatment, 2.43 %<br>Cre$^+$ atropine, 1.27 %<br>Cre$^-$ no treatment, 3.01 %
**5c**	Absolute values	Ctrl *vs*. TGOT	Wilcoxon matched-pairs signed rank test	n = 12	p = 0.0010	Ctrl, 6.51 bpm<br>TGOT, 8.66 bpm	Ctrl, 0.81 bpm<br>TGOT, 0.84 bpm
**5c**	Delta changes	TGOT	n/a	n = 12	n/a	+47.00 %	20.40 %
**5e**	Absolute values	Ctrl *vs*. TGOT	Paired t-test	n = 5	p = 0.0368	Ctrl, 14.26 bpm<br>TGOT, 19.29 bpm	Ctrl, 3.07 bpm<br>TGOT, 3.46 bpm
**5e**	Delta changes	TGOT	n/a	n = 5	n/a	+40.07 %	11.55 %
**5h**	Absolute values	Ctrl *vs*. TGOT	Paired t-test	n = 7	p < 0.0001	Ctrl, -52.14 mV<br>TGOT, -47.23 mV	Ctrl, 2.08 mV<br>TGOT, 2.46 mV
**5h**	Delta changes	TGOT	n/a	n = 7	n/a	+4.91 %	0.51 %
**5l**	Absolute values	Ctrl *vs*. TGOT	Paired t-test	n = 10	p = 0.0026	Ctrl, 0.033 nanoAmp<br>TGOT, 0.051 nanoAmp	Ctrl, 0.007 nanoAmp<br>TGOT, 0.010 nanoAmp
**5l**	Delta changes	TGOT *vs*. Bic *vs*. Stry	Friedman test, Dunn’s multiple comparison	n = 8	TGOT *vs*. Bic, p > 0.1<br>Bic *vs*. Stry, p = 0.0081<br>TGOT *vs*. Stry, p = 0.0081	Delta TGOT, +53.80 %<br>Delta Bic, +52.01 %<br>Delta Stry, -175.42 %	Delta TGOT, 10.14 %<br>Delta Bic, 21.24 %<br>Delta Stry, 39.51 %
**6c**	Absolute values (TGOT)	Ctrl vs. TGOT	Wilcoxon matched-pairs signed rank test or paired t-test (respiratory frequency)	n = 15	**RespHRV**, p = 0.0006<br>**mHR**, p = 0.0346<br>**Respiratory freq**, p = 0.0638<br>**Respiratory ampl**, p = 0.0067	**RespHRV:**<br>Ctrl, 1.45 bpm<br>TGOT, 2.10 bpm<br>**mHR:**<br>Ctrl, 295.06 bpm<br>TGOT, 291.96 bpm<br>**Respiratory freq:**<br>Ctrl, 55.39 cpm<br>TGOT, 54.22 cpm<br>**Respiratory ampl:**<br>Ctrl, 19.48 (a.u.)<br>TGOT, 21.51 (a.u.)	**RespHRV:**<br>Ctrl, 0.45 bpm<br>TGOT, 0.67 bpm<br>**mHR:**<br>Ctrl, 8.60 bpm<br>TGOT, 8.33 bpm<br>**Respiratory freq:**<br>Ctrl, 2.74 cpm<br>TGOT, 2.95 cpm<br>**Respiratory ampl:**<br>Ctrl, 2.98 (a.u.)<br>TGOT, 3.67 (a.u.)
**6c**	Delta changes	Inter-group analysis: TGOT *vs*. Vehicle	Mann-Whitney test or unpaired t-test (mHR)	TGOT, n = 15; Vehicle, n = 10	**RespHRV**, p = 0.0003	**RespHRV:**<br>TGOT, +52.65 %<br>Vehicle, -5.44 %	**RespHRV:**<br>TGOT, 15.82 %<br>Vehicle, 4.37 %

					**mHR**, p > 0.1<br><br><br>**Respiratory freq**, p = 0.0476<br><br><br>**Respiratory ampl**, p = 0.0101	**mHR:**<br>TGOT, -3.10 bpm<br>Vehicle, -5.46 bpm<br>**Respiratory freq:**<br>TGOT, -2.34 %<br>Vehicle, +0.48 %<br>**Respiratory ampl:**<br>TGOT, +7.02 %<br>Vehicle, -1.76 %	**mHR:**<br>TGOT, 1.31 bpm<br>Vehicle, 1.96 bpm<br>**Respiratory freq:**<br>TGOT, 1.19 %<br>Vehicle, 0.56 %<br>**Respiratory ampl:**<br>TGOT, 4.07 %<br>Vehicle, 1.23 %
**6f**	Absolute values (TGOT)	Ctrl *vs*. TGOT	Paired t-test or Wilcoxon matched-pairs	RespHRV, n = 16; mHR, n = 18; PNA frequency, n = 19; PNA amplitude, n = 18; VNA, n = 7; tSNA, n = 8; mPP, n = 18	**RespHRV**, p < 0.0001<br><br><br>**mHR**, p > 0.1<br><br><br>**PNA frequency**, p = 0.0627<br><br><br>**PNA amplitude**, p > 0.1<br><br><br>**VNA amplitude**, p = 0.0578<br><br><br>**Mean tSNA activity** p > 0.1<br><br><br>**mPP,** p > 0.1	**RespHRV:**<br>Ctrl, 1.40 bpm<br>TGOT, 2.60 bpm<br>**mHR:**<br>Ctrl, 340.74 bpm<br>TGOT, 338.71 bpm<br>**PNA frequency:**<br>Ctrl, 22.32 cpm<br>TGOT, 24.27 cpm<br>**PNA amplitude:**<br>Ctrl, 10.35 µV<br>TGOT, 9.65 µV<br>**VNA amplitude:**<br>Ctrl, 1.69 µV<br>TGOT, 1.40 µV<br>**Mean tSNA activity:**<br>Ctrl, 3.00 µV<br>TGOT, 3.06 µV<br>**mPP:**<br>Ctrl, 63.11 mmHg<br>TGOT, 61.63 mmHg	**RespHRV:**<br>Ctrl, 0.17 bpm<br>TGOT, 0.31 bpm<br>**mHR:**<br>Ctrl, 7.15 bpm<br>TGOT, 7.02 bpm<br>**PNA frequency:**<br>Ctrl, 1.89 cpm<br>TGOT, 2.37 cpm<br>**PNA amplitude:**<br>Ctrl, 1.03 µV<br>TGOT, 1.10 µV<br>**VNA amplitude:**<br>Ctrl, 0.23 µV<br>TGOT, 0.20 µV<br>**Mean tSNA activity:**<br>Ctrl, 0.28 µV<br>TGOT, 0.29 µV<br>**mPP:**<br>Ctrl, 5.37 mmHg<br>TGOT, 5.84 mmHg
**6f**	Delta changes	Inter-group analysis: TGOT *vs*. Vehicle	Mann-Whitney test or unpaired t-test	**TGOT**<br>RespHRV, n = 16; mHR, n = 18; PNA frequency, n = 19; PNA amplitude,	**RespHRV**, p = 0.0003<br><br><br>**mHR**, p = 0.0986<br><br><br>**PNA frequency**, p > 0.1	**RespHRV:**<br>TGOT, +105.29 %<br>Vehicle, -8.46 %<br>**mHR:**<br>TGOT, -2.03 bpm<br>Vehicle, +3.98 bpm<br>**PNA frequency:**<br>TGOT, +8.18 %	**RespHRV:**<br>TGOT, 18.27 %<br>Vehicle, 12.04 %<br>**mHR:**<br>TGOT, 1.59 bpm<br>Vehicle, 3.91 bpm<br>**PNA frequency:**<br>TGOT, 4.05 %

				n = 18;<br>VNA, n = 7;<br>tSNA, n = 8;<br>mPP, n = 18<br>**Vehicle**<br>RespHRV, n = 8; mHR, n=8;<br>PNA frequency, n = 9;<br>PNA amplitude, n = 8;<br>VNA, n = 9;<br>tSNA, n = 8;<br>mPP, n = 8	**PNA amplitude**, $p > 0.1$<br><br>**VNA amplitude**, $p > 0.1$<br><br>**Mean tSNA activity,** $p > 0.1$<br><br>**mPP,** $p > 0.1$	Vehicle, +9.25 %<br>**PNA amplitude:**<br>TGOT, -4.54 %<br>Vehicle, -2.58 %<br>**VNA amplitude:**<br>TGOT, -15.74 %<br>Vehicle, -8.39 %<br>**Mean tSNA activity:**<br>TGOT, +2.04 %<br>Vehicle, +9.53 %<br>**mPP:**<br>TGOT, -2.97%<br>Vehicle, -0.81 %	Vehicle, 5.30 %<br>**PNA amplitude:**<br>TGOT, 6.54 %<br>Vehicle, 3.93 %<br>**VNA amplitude:**<br>TGOT, 5.12 %<br>Vehicle, 4.49 %<br>**Mean tSNA activity:**<br>TGOT, 1.23 %<br>Vehicle, 6.33 %<br>**mPP:**<br>TGOT, 1.33 %<br>Vehicle, 1.80 %
**6g**	Delta changes	Delta RespHRV *vs*. Pre-injection RespHRV	Pearson correlation analysis, simple linear regression plotted	n = 16	**Pre-stim RespHRV *vs*. Pre-stim mHR:**<br>$p = 0.0050$, $R^2 = 42$ %	n/a	n/a
**6i**	Absolute values (TGOT)	Ctrl *vs*. TGOT	Paired t-test	n = 7	$p = 0.0020$	**CVBA activity:**<br>Ctrl, 0.115 µV<br>TGOT, 0.197 µV	**CVBA activity:**<br>Ctrl, 0.0016 µV<br>TGOT, 0.028 µV
**6i**	Delta changes	TGOT	n/a	n = 7	n/a	+73.63 %	10.14 %
**Ext. Data Fig. 9b**	Absolute values (vehicle)	Ctrl *vs*. Vehicle	Paired t-test or Wilcoxon matched-pairs signed rank test (RespHRV)	n = 10	**RespHRV**, $p > 0.1$<br><br>**mHR**, $p = 0.0209$<br><br>**Respiratory freq**, $p > 0.1$<br><br>**Respiratory ampl**, $p > 0.1$	**RespHRV:**<br>Ctrl, 0.95 bpm<br>Vehicle, 0.89 bpm<br>**mHR:**<br>Ctrl, 318.85 bpm<br>Vehicle, 313.38 bpm<br>**Respiratory freq:**<br>Ctrl, 50.45 cpm<br>Vehicle, 50.69 cpm<br>**Respiratory ampl:**<br>Ctrl, 18.41 (a.u.)<br>Vehicle, 18.11 (a.u.)	**RespHRV:**<br>Ctrl, 0.22 bpm<br>Vehicle, 0.20 bpm<br>**mHR:**<br>Ctrl, 10.05 bpm<br>Vehicle, 8.59 bpm<br>**Respiratory freq:**<br>Ctrl, 0.90 cpm<br>Vehicle, 0.97 cpm<br>**Respiratory ampl:**<br>Ctrl, 3.36 (a.u.)<br>Vehicle, 3.37 (a.u.)

**Ext. Data Fig. 9e**	Absolute values (vehicle)	Ctrl *vs*. vehicle	Paired t-test or Wilcoxon matched-pairs	HR, n = 8;<br>PNA frequency, n = 9;<br>PNA amplitude, n = 8;<br>VNA, n = 9;<br>tSNA,n = 8;<br>mPP, n = 8	**RespHRV**, p > 0.1<br>**mHR**, p > 0.1<br>**PNA frequency**, p = 0.0868<br>**PNA amplitude**, p > 0.1<br>**VNA amplitude**, p = 0.0547<br>**mPP,** p > 0.1<br>**Mean SNA activity,** p > 0.1<br>**Tonic SNA**, p = 0.0455<br>**RespSNA,** p > 0.1	**RespHRV:**<br>Ctrl, 1.50 bpm<br>Vehicle, 1.06 bpm<br>**mHR:**<br>Ctrl, 341.19 bpm<br>Vehicle, 345.17 bpm<br>**PNA frequency:**<br>Ctrl, 28.87 cpm<br>Vehicle, 30.63 cpm<br>**PNA amplitude:**<br>Ctrl, 10.13 µV<br>Vehicle, 9.53 µV<br>**VNA amplitude:**<br>Ctrl, 1.41 µV<br>Vehicle, 1.25 %<br>**mPP:**<br>Ctrl, 72.97 mmHg<br>Vehicle, 72.49 mmHg<br>**Mean tSNA activity:**<br>Ctrl, 4.44 µV<br>Vehicle, 4.76 µV<br>**Tonic SNA:**<br>Ctrl, 4.10 µV<br>Vehicle, 4.63 µV<br>**RespSNA:**<br>Ctrl, 0.78 µV<br>Vehicle, 0.73 µV	**RespHRV:**<br>Ctrl, 0.45 bpm<br>Vehicle, 0.10 bpm<br>**mHR:**<br>Ctrl, 17.58 bpm<br>Vehicle, 19.11 bpm<br>**PNA frequency:**<br>Ctrl, 4.59 cpm<br>Vehicle, 4.28 cpm<br>**PNA amplitude:**<br>Ctrl, 2.52 µV<br>Vehicle, 2.27 µV<br>**VNA amplitude:**<br>Ctrl, 0.20 µV<br>Vehicle, 0.16 %<br>**mPP:**<br>Ctrl, 3.71 mmHg<br>Vehicle, 4.09 mmHg<br>**Mean tSNA activity:**<br>Ctrl, 0.65 µV<br>Vehicle, 0.60 µV<br>**Tonic SNA:**<br>Ctrl, 0.41 µV<br>Vehicle, 0.45 µV<br>**RespSNA:**<br>Ctrl, 0.26 µV<br>Vehicle, 0.32 µV
**Ext. Data Fig. 9f**	Absolute values (TGOT)	Ctrl *vs*. TGOT	Paired t-test	n = 8	**RespSNA,** p > 0.1<br>**Tonic SNA**, p > 0.1	**RespSNA:**<br>Ctrl, 0.56 (a.u.)<br>TGOT, 0.57 (a.u.)<br>**Tonic SNA:**<br>Ctrl, 2.86 µV<br>TGOT, 2.87 µV	**RespSNA:**<br>Ctrl, 0.12 (a.u.)<br>TGOT, 0.17 (a.u.)<br>**Tonic SNA:**<br>Ctrl, 0.26 µV<br>TGOT, 0.25 µV
**Ext. Data Fig. 9f**	Delta changes	Inter-group analysis: TGOT *vs*. Vehicle	Unpaired t-test (RespSNA) or Mann-Whitney test (tonic SNA)	TGOT, n = 8;<br>vehicle, n = 8	**RespSNA,** p > 0.1<br>**Tonic SNA**, p = 0.0207	**RespSNA:**<br>TGOT, -5.02 %<br>Vehicle, -10.81 %<br>**Tonic SNA:**<br>TGOT, +0.86 %	**RespSNA:**<br>TGOT, 11.69 %<br>Vehicle, 10.54 %<br>**Tonic SNA:**<br>TGOT, 1.17 %

						Vehicle, +13.75 %	Vehicle, 5.61 %
**7b**	Absolute values	Ctrl *vs*. C21 *vs*. Wash	Repeated Measures one-way ANOVA with the Geisser-Greenhouse correction, Tukey's multiple comparison	Cre^{+}, n = 3 (11 cells);<br>Cre^{-}, n = 2 (6 cells)	**Cre^{+}**<br>Ctrl vs. C21, $p < 0.0001$<br>Ctrl vs. Wash, $p = 0.0004$<br>C21 vs. Wash, $p = 0.0033$<br><br>**Cre^{-}**<br>Ctrl vs. C21, $p > 0.1$<br>Ctrl vs. Wash, $p > 0.1$<br>C21 vs. Wash, $p > 0.1$	**Cre^{+}:**<br>Ctrl, 82.18 AP/min<br>C21, 30.30 AP/min<br>Wash, 61.91 AP/min<br><br>**Cre^{-}:**<br>Ctrl, 191.83 AP/min<br>C21, 191.50 AP/min<br>Wash, 188.94 AP/min	**Cre^{+}:**<br>Ctrl, 13.01 AP/min<br>C21, 8.91 AP/min<br>Wash, 13.10 AP/min<br><br>**Cre^{-}:**<br>Ctrl, 47.44 AP/min<br>C21, 48.29 AP/min<br>Wash, 50.66 AP/min
**7e**	Absolute values (Cre^{+})	Pre-stress period, C21 *vs.* vehicle	Paired t-test	n = 7	**RespHRV**, $p > 0.1$<br><br><br>**mHR**, $p = 0.0132$<br><br><br>**mBP,** $p = 0.0680$<br><br><br>**Respiratory freq**, $p > 0.1$<br><br><br>**Respiratory ampl**, $p > 0.1$	**RespHRV:**<br>C21, 28.05 bpm<br>Vehicle, 25.12 bpm<br>**mHR:**<br>C21, 436.24 bpm<br>Vehicle, 475.59 bpm<br>**mBP:**<br>C21, 96.46 mmHg<br>Vehicle, 91.72 mmHg<br>**Respiratory freq:**<br>C21, 194.58 cpm<br>Vehicle, 201.62 cpm<br>**Respiratory ampl:**<br>C21, 0.44 (a.u.)<br>Vehicle, 0.48 (a.u.)	**RespHRV:**<br>C21, 3.18 bpm<br>Vehicle, 3.69 bpm<br>**mHR:**<br>C21, 16.37 bpm<br>Vehicle, 16.67 bpm<br>**mBP:**<br>C21, 4.73 mmHg<br>Vehicle, 4.91 mmHg<br>**Respiratory freq:**<br>C21, 6.43 cpm<br>Vehicle, 5.48 cpm<br>**Respiratory ampl:**<br>C21, 0.07 (a.u.)<br>Vehicle, 0.07 (a.u.)
**7f**	Delta changes (Cre^{+})	Stress *vs*. 30min *vs*. 60min, C21 *vs.* vehicle	Repeated Measures two-way ANOVA, Sidak's multiple comparison	n = 7	**RespHRV:**<br>**C21**<br>Stress *vs*. 30min, $p > 0.1$<br>Stress *vs*. 60min, $p = 0.0010$<br>30min *vs*. 60min, $p = 0.0995$<br>**Vehicle**<br>Stress *vs*. 30min, $p = 0.0029$<br>Stress *vs*. 60min, $p = 0.0025$<br>30min *vs*. 60min, $p > 0.1$<br>**C21 *vs.* Vehicle**<br>Stress C21 *vs*. Stress vehicle, $p > 0.1$<br>Stress C21 *vs*. 30min vehicle, $p > 0.1$<br>Stress C21 *vs*. 60min vehicle, $p > 0.1$	**RespHRV:**<br>**C21**<br>Stress, -50.84 %<br>30 min, -32.64 %<br>60 min, -9.84 %<br>**Vehicle**<br>Stress, -44.99 %<br>30 min, -7.80 %<br>60 min, -7.17 %	**RespHRV:**<br>**C21**<br>Stress, 3.76 %<br>30 min, 3.41 %<br>60 min, 8.62 %<br>**Vehicle**<br>Stress, 6.52 %<br>30 min, 8.94 %<br>60 min, 9.98 %

					30min C21 *vs*. stress vehicle, p > 0.1<br>30min C21 *vs*. 30min vehicle, p = 0.0432<br>30min C21 *vs*. 60min vehicle, p > 0.1<br>60min C21 *vs*. stress vehicle, p > 0.1<br>60min C21 *vs*. 30min vehicle, p > 0.1<br>60min C21 *vs*. 60min vehicle, p > 0.1<br><br>**mHR:**<br>**C21**<br>Stress *vs*. 30min, p < 0.0001<br>Stress *vs*. 60min, p < 0.0001<br>30min *vs*. 60min, p > 0.1<br>**Vehicle**<br>Stress *vs*. 30min, p < 0.0001<br>Stress *vs*. 60min, p < 0.0001<br>30min *vs*. 60min, p > 0.1<br>**C21 *vs*. Vehicle**<br>Stress C21 *vs*. Stress vehicle, p = 0.0044<br>Stress C21 *vs*. 30min vehicle, p > 0.1<br>Stress C21 *vs*. 60min vehicle, p > 0.1<br>30min C21 *vs*. stress vehicle, p > 0.1<br>30min C21 *vs*. 30min vehicle, p = 0.0053<br>30min C21 *vs*. 60min vehicle, p > 0.1<br>60min C21 *vs*. stress vehicle, p > 0.1<br>60min C21 *vs*. 30min vehicle, p > 0.1<br>60min C21 *vs*. 60min vehicle, p > 0.1<br><br>**mBP:**<br>**C21**<br>Stress *vs*. 30min, p < 0.0001<br>Stress *vs*. 60min, p < 0.0001<br>30min *vs*. 60min, p = 0.0002<br>**Vehicle**<br>Stress *vs*. 30min, p = 0.0001<br>Stress *vs*. 60min, p < 0.0001<br>30min *vs*. 60min, p = 0.0011<br>**C21 *vs*. Vehicle**	**mHR:**<br>**C21**<br>Stress, +260.66 bpm<br>30 min, +138.83 bpm<br>60 min, +95.08 bpm<br>**Vehicle**<br>Stress, +206.18 bpm<br>30 min, +85.65 bpm<br>60 min, +87.14 bpm<br><br>**mBP:**<br>**C21**<br>Stress, +39.18 %<br>30 min, +17.86 %<br>60 min, -1.49 %<br>**Vehicle**<br>Stress, +34.93 %<br>30 min, +14.94 %<br>60 min, -1.78 %	**mHR:**<br>**C21**<br>Stress, 20.60 bpm<br>30 min, 11.62 bpm<br>60 min, 15.58 bpm<br>**Vehicle**<br>Stress, 16.26 bpm<br>30 min, 17.15 bpm<br>60 min, 9.50 bpm<br><br>**mBP:**<br>**C21**<br>Stress, 4.08 %<br>30 min, 1.55 %<br>60 min, 4.82 %<br>**Vehicle**<br>Stress, 4.82 %<br>30 min, 2.38 %<br>60 min, 1.69 %

					Stress C21 *vs*. Stress vehicle, $p = 0.0792$<br>Stress C21 *vs*. 30min vehicle, $p > 0.1$<br>Stress C21 *vs*. 60min vehicle, $p > 0.1$<br>30min C21 *vs*. stress vehicle, $p > 0.1$<br>30min C21 *vs*. 30min vehicle, $p > 0.1$<br>30min C21 *vs*. 60min vehicle, $p > 0.1$<br>60min C21 *vs*. stress vehicle, $p > 0.1$<br>60min C21 *vs*. 30min vehicle, $p > 0.1$<br>60min C21 *vs*. 60min vehicle, $p > 0.1$		
					**Respiratory freq:**<br>**C21**<br>Stress *vs*. 30min, $p < 0.0001$<br>Stress *vs*. 60min, $p < 0.0001$<br>30min *vs*. 60min, $p > 0.1$<br>**Vehicle**<br>Stress *vs*. 30min, $p < 0.0001$<br>Stress *vs*. 60min, $p < 0.0001$<br>30min *vs*. 60min, $p > 0.1$<br>**C21 *vs*. Vehicle**<br>Stress C21 *vs*. Stress vehicle, $p > 0.1$<br>Stress C21 *vs*. 30min vehicle, $p > 0.1$<br>Stress C21 *vs*. 60min vehicle, $p > 0.1$<br>30min C21 *vs*. stress vehicle, $p > 0.1$<br>30min C21 *vs*. 30min vehicle, $p > 0.1$<br>30min C21 *vs*. 60min vehicle, $p > 0.1$<br>60min C21 *vs*. stress vehicle, $p > 0.1$<br>60min C21 *vs*. 30min vehicle, $p > 0.1$<br>60min C21 *vs*. 60min vehicle, $p > 0.1$	**Respiratory freq:**<br>**C21**<br>Stress, +82.87 %<br>30 min, +12.56 %<br>60 min, +7.85 %<br>**Vehicle**<br>Stress, +75.53 %<br>30 min, +14.80 %<br>60 min, +1.31 %	**Respiratory freq:**<br>**C21**<br>Stress, 12.68 %<br>30 min, 6.27 %<br>60 min, 4.64 %<br>**Vehicle**<br>Stress, 7.75 %<br>30 min, 4.33 %<br>60 min, 2.01 %
					**Respiratory ampl:**<br>**C21**<br>Stress *vs*. 30min, $p < 0.0001$<br>Stress *vs*. 60min, $p < 0.0001$<br>30min *vs*. 60min, $p > 0.1$<br>**Vehicle**<br>Stress *vs*. 30min, $p < 0.0001$<br>Stress *vs*. 60min, $p < 0.0001$	**Respiratory ampl:**<br>**C21**<br>Stress, +198.26 %<br>30 min, +34.57 %<br>60 min, +3.67 %<br>**Vehicle**<br>Stress, +177.27 %<br>30 min, +37.57 %	**Respiratory ampl:**<br>**C21**<br>Stress, 32.19 %<br>30 min, 5.20 %<br>60 min, 4.01 %<br>**Vehicle**<br>Stress, 14.32 %<br>30 min, 8.79 %

					30min *vs*. 60min, p > 0.1<br>**C21 *vs*. Vehicle**<br>Stress C21 *vs*. Stress vehicle, p > 0.1<br>Stress C21 *vs*. 30min vehicle, p > 0.1<br>Stress C21 *vs*. 60min vehicle, p > 0.1<br>30min C21 *vs*. stress vehicle, p > 0.1<br>30min C21 *vs*. 30min vehicle, p > 0.1<br>30min C21 *vs*. 60min vehicle, p > 0.1<br>60min C21 *vs*. stress vehicle, p > 0.1<br>60min C21 *vs*. 30min vehicle, p > 0.1<br>60min C21 *vs*. 60min vehicle, p > 0.1	60 min, -0.47 %	60 min, 3.73 %
**Ext. Data Fig. 10b**	Absolute values (Cre^-)	Pre-stress period, C21 *vs*. vehicle	Paired t-test	n = 5	**RespHRV**, p > 0.1<br><br><br>**mHR**, p = 0.0049<br><br><br>**mBP,** p > 0.1<br><br><br>**Respiratory freq**, p > 0.1<br><br><br>**Respiratory ampl**, p > 0.1	**RespHRV:**<br>C21, 19.46 bpm<br>Vehicle, 20.26 bpm<br>**mHR:**<br>C21, 455.15 bpm<br>Vehicle, 487.81 bpm<br>**mBP:**<br>C21, 104.84 mmHg<br>Vehicle, 103.16 mmHg<br>**Respiratory freq:**<br>C21, 184.90 cpm<br>Vehicle, 183.43 cpm<br>**Respiratory ampl:**<br>C21, 0.35 (a.u.)<br>Vehicle, 0.34 (a.u.)	**RespHRV:**<br>C21, 1.97 bpm<br>Vehicle, 1.76 bpm<br>**mHR:**<br>C21, 15.35 bpm<br>Vehicle, 19.37 bpm<br>**mBP:**<br>C21, 3.09 mmHg<br>Vehicle, 3.00 mmHg<br>**Respiratory freq:**<br>C21, 9.10 cpm<br>Vehicle, 10.89 cpm<br>**Respiratory ampl:**<br>C21, 0.02 (a.u.)<br>Vehicle, 0.05 (a.u.)
**Ext. Data Fig. 10c**	Delta changes (Cre^-)	Stress *vs*. 30min vs. 60min, C21 *vs*. vehicle	Repeated Measures two-way ANOVA, Sidak's multiple comparison	n = 5	**RespHRV:**<br>**C21**<br>Stress *vs*. 30min, p = 0.0401<br>Stress *vs*. 60min, p = 0.0363<br>30min *vs*. 60min, p > 0.1<br>**Vehicle**<br>Stress *vs*. 30min, p > 0.1<br>Stress *vs*. 60min, p = 0.0257<br>30min *vs*. 60min, p > 0.1<br>**C21 *vs*. Vehicle**<br>Stress C21 *vs*. Stress vehicle, p > 0.1<br>Stress C21 *vs*. 30min vehicle, p > 0.1	**RespHRV:**<br>**C21**<br>Stress, -41.70 %<br>30 min, -15.45 %<br>60 min, -15.01 %<br>**Vehicle**<br>Stress, -41.83 %<br>30 min, -24.64 %<br>60 min, -13.66 %	**RespHRV:**<br>**C21**<br>Stress, 8.76 %<br>30 min, 6.15 %<br>60 min, 9.80 %<br>**Vehicle**<br>Stress, 4.78 %<br>30 min, 4.58 %<br>60 min, 6.06 %

					Stress C21 *vs*. 60min vehicle, p > 0.1<br>30min C21 *vs*. stress vehicle, p > 0.1<br>30min C21 *vs*. 30min vehicle, p > 0.1<br>30min C21 *vs*. 60min vehicle, p > 0.1<br>60min C21 *vs*. stress vehicle, p > 0.1<br>60min C21 *vs*. 30min vehicle, p > 0.1<br>60min C21 *vs*. 60min vehicle, p > 0.1<br><br>**mHR:**<br>**C21**<br>Stress *vs*. 30min, p < 0.0001<br>Stress *vs*. 60min, p < 0.0001<br>30min *vs*. 60min, p > 0.1<br>**Vehicle**<br>Stress *vs*. 30min, p < 0.0001<br>Stress *vs*. 60min, p < 0.0001<br>30min *vs*. 60min, p > 0.1<br>**C21 *vs*. Vehicle**<br>Stress C21 *vs*. Stress vehicle, p > 0.1<br>Stress C21 *vs*. 30min vehicle, p > 0.1<br>Stress C21 *vs*. 60min vehicle, p > 0.1<br>30min C21 *vs*. stress vehicle, p > 0.1<br>30min C21 *vs*. 30min vehicle, p > 0.1<br>30min C21 *vs*. 60min vehicle, p > 0.1<br>60min C21 *vs*. stress vehicle, p > 0.1<br>60min C21 *vs*. 30min vehicle, p > 0.1<br>60min C21 *vs*. 60min vehicle, p > 0.1<br><br>**mBP:**<br>**C21**<br>Stress *vs*. 30min, p < 0.0001<br>Stress *vs*. 60min, p < 0.0001<br>30min *vs*. 60min, p = 0.0044<br>**Vehicle**<br>Stress vs. 30min, p = 0.0270<br>Stress vs. 60min, p < 0.0001<br>30min vs. 60min, p = 0.0009<br>**C21 *vs*. Vehicle**	**mHR:**<br>**C21**<br>Stress, +240.55 bpm<br>30 min, +78.50 bpm<br>60 min, +60.08 bpm<br>**Vehicle**<br>Stress, +225.54 bpm<br>30 min, +95.99 bpm<br>60 min, +43.57 bpm<br><br>**mBP:**<br>**C21**<br>Stress, +36.04 %<br>30 min, +12.02 %<br>60 min, -4.46 %<br>**Vehicle**<br>Stress, +29.92 %<br>30 min, +16.90 %<br>60 min, -2.40 %	**mHR:**<br>**C21**<br>Stress, 13.19 bpm<br>30 min, 16.95 bpm<br>60 min, 20.95 bpm<br>**Vehicle**<br>Stress, 18.32 bpm<br>30 min, 11.32 bpm<br>60 min, 9.97 bpm<br><br>**mBP:**<br>**C21**<br>Stress, 3.89 %<br>30 min, 4.80 %<br>60 min, 1.83 %<br>**Vehicle**<br>Stress, 2.12 %<br>30 min, 4.14 %<br>60 min, 0.27 %

					Stress C21 *vs*. Stress vehicle, p > 0.1<br>Stress C21 *vs*. 30min vehicle, p > 0.1<br>Stress C21 *vs*. 60min vehicle, p > 0.1<br>30min C21 *vs*. stress vehicle, p > 0.1<br>30min C21 *vs*. 30min vehicle, p > 0.1<br>30min C21 *vs*. 60min vehicle, p > 0.1<br>60min C21 *vs*. stress vehicle, p > 0.1<br>60min C21 *vs*. 30min vehicle, p > 0.1<br>60min C21 *vs*. 60min vehicle, p > 0.1		
					**Respiratory freq:**<br>**C21**<br>Stress *vs*. 30min, p < 0.0001<br>Stress *vs*. 60min, p < 0.0001<br>30min *vs*. 60min, p > 0.1<br>**Vehicle**<br>Stress *vs*. 30min, p < 0.0001<br>Stress *vs*. 60min, p < 0.0001<br>30min *vs*. 60min, p > 0.1<br>**C21 *vs*. Vehicle**<br>Stress C21 *vs*. Stress vehicle, p > 0.1<br>Stress C21 *vs*. 30min vehicle, p > 0.1<br>Stress C21 *vs*. 60min vehicle, p > 0.1<br>30min C21 *vs*. stress vehicle, p > 0.1<br>30min C21 *vs*. 30min vehicle, p > 0.1<br>30min C21 *vs*. 60min vehicle, p > 0.1<br>60min C21 *vs*. stress vehicle, p > 0.1<br>60min C21 *vs*. 30min vehicle, p > 0.1<br>60min C21 *vs*. 60min vehicle, p > 0.1	**Respiratory freq:**<br>**C21**<br>Stress, +78.27 %<br>30 min, +11.24 %<br>60 min, +6.47 %<br>**Vehicle**<br>Stress, +90.58 %<br>30 min, +18.15 %<br>60 min, +7.33 %	**Respiratory freq:**<br>**C21**<br>Stress, 12.29 %<br>30 min, 4.71 %<br>60 min, 3.35 %<br>**Vehicle**<br>Stress, 7.23 %<br>30 min, 5.30 %<br>60 min, 3.16 %
					**Respiratory ampl:**<br>**C21**<br>Stress *vs*. 30min, p < 0.0001<br>Stress *vs*. 60min, p < 0.0001<br>30min *vs*. 60min, p > 0.1<br>**Vehicle**<br>Stress *vs*. 30min, p < 0.0001<br>Stress *vs*. 60min, p < 0.0001	**Respiratory ampl:**<br>**C21**<br>Stress, +165.35 %<br>30 min, +31.60 %<br>60 min, +10.51 %<br>**Vehicle**<br>Stress, +156.33 %<br>30 min, +25.89 %	**Respiratory ampl:**<br>**C21**<br>Stress, 25.65 %<br>30 min, 15.82 %<br>60 min, 8.59 %<br>**Vehicle**<br>Stress, 22.81 %<br>30 min, 10.79 %

					30min *vs*. 60min, p > 0.1<br>**C21 *vs*. Vehicle**<br>Stress C21 *vs*. Stress vehicle, p > 0.1<br>Stress C21 *vs*. 30min vehicle, p > 0.1<br>Stress C21 *vs*. 60min vehicle, p > 0.1<br>30min C21 *vs*. stress vehicle, p > 0.1<br>30min C21 *vs*. 30min vehicle, p > 0.1<br>30min C21 *vs*. 60min vehicle, p > 0.1<br>60min C21 *vs*. stress vehicle, p > 0.1<br>60min C21 *vs*. 30min vehicle, p > 0.1<br>60min C21 *vs*. 60min vehicle, p > 0.1	60 min, -0.19 %	60 min, 4.67 %
**Suppl. Fig. 2a**	Absolute values	RespHRV per mouse over consecutive weeks	n/a	n = 7	n/a	**Mouse 1**<br>Week 1, 21.47 bpm<br>Week 2, 30.46 bpm<br>**Mouse 2**<br>Week 1, 21.23 bpm<br>Week 2, 24.17 bpm<br>**Mouse 3**<br>Week 1, 19.76 bpm<br>Week 2, 19.83 bpm<br>**Mouse 4**<br>Week 1, 14.82 bpm<br>Week 2, 14.24 bpm<br>**Mouse 5**<br>Week 1, 26.69 bpm<br>Week 2, 28.63 bpm<br>**Mouse 6**<br>Week 1, 19.12 bpm<br>Week 2, 18.34 bpm<br>Week 3, 18.19 bpm<br>Week 4, 16.57 bpm<br>**Mouse 7**<br>Week 1, 12.50 bpm<br>Week 2, 13.21 bpm<br>Week 3, 7.03 bpm<br>Week 4, 11.19 bpm	**Mouse 1**<br>Week 1, 0.79 bpm<br>Week 2, 1.32 bpm<br>**Mouse 2**<br>Week 1, 0.81 bpm<br>Week 2, 0.88 bpm<br>**Mouse 3**<br>Week 1, 0.62 bpm<br>Week 2, 0.89 bpm<br>**Mouse 4**<br>Week 1, 0.69 bpm<br>Week 2, 0.70 bpm<br>**Mouse 5**<br>Week 1, 1.83 bpm<br>Week 2, 1.00 bpm<br>**Mouse 6**<br>Week 1, 0.64 bpm<br>Week 2, 1.43 bpm<br>Week 3, 1.08 bpm<br>Week 4, 1.17 bpm<br>**Mouse 7**<br>Week 1, 0.57 bpm<br>Week 2, 0.89 bpm<br>Week 3, 0.71 bpm<br>Week 4, 1.19 bpm

**Suppl. Fig. 2b**	Absolute values	RespHRV Week 1 *vs.* Week 2	Wilcoxon matched-pairs signed rank test	n = 7	$p > 0.1$	Week 1, 19.37 bpm<br>Week 2, 21.27 bpm	Week 1, 1.76 bpm<br>Week 2, 2.55 bpm
