# Supplementary material for: Oxytocin modulates respiratory heart rate variability through a hypothalamus–brainstem–heart neuronal pathway
Source: Nat Neurosci. 2025 Oct 20;28(11):2247–61. doi: 10.1038/s41593-025-02074-2 (PMC12586189; doi:10.1038/s41593-025-02074-2)
Supplement: Supplementary file 2 — Reporting Summary [file 41593_2025_2074_MOESM2_ESM.pdf]

nature portfolio

Corresponding author(s): Clément Menuet

Last updated by author(s): Jul 21, 2025

Reporting Summary

Nature Portfolio wishes to improve the reproducibility of the work that we publish. This form provides structure for consistency and transparency in reporting. For further information on Nature Portfolio policies, see our Editorial Policies and the Editorial Policy Checklist.

Statistics

For all statistical analyses, confirm that the following items are present in the figure legend, table legend, main text, or Methods section.

n/a	Confirmed	
☐	☒	The exact sample size (*n*) for each experimental group/condition, given as a discrete number and unit of measurement
☐	☒	A statement on whether measurements were taken from distinct samples or whether the same sample was measured repeatedly
☐	☒	The statistical test(s) used AND whether they are one- or two-sided<br>*Only common tests should be described solely by name; describe more complex techniques in the Methods section.*
☒	☐	A description of all covariates tested
☐	☒	A description of any assumptions or corrections, such as tests of normality and adjustment for multiple comparisons
☐	☒	A full description of the statistical parameters including central tendency (e.g. means) or other basic estimates (e.g. regression coefficient) AND variation (e.g. standard deviation) or associated estimates of uncertainty (e.g. confidence intervals)
☐	☒	For null hypothesis testing, the test statistic (e.g. *F*, *t*, *r*) with confidence intervals, effect sizes, degrees of freedom and *P* value noted<br>*Give P values as exact values whenever suitable.*
☒	☐	For Bayesian analysis, information on the choice of priors and Markov chain Monte Carlo settings
☒	☐	For hierarchical and complex designs, identification of the appropriate level for tests and full reporting of outcomes
☐	☒	Estimates of effect sizes (e.g. Cohen's *d*, Pearson's *r*), indicating how they were calculated

Our web collection on statistics for biologists contains articles on many of the points above.

Software and code

Policy information about availability of computer code

Data collection	Spike2 software (V10, Cambridge Electrical Design, Cambridge, UK), ZEN imaging software (V2.6 or 3.7, blue edition, Zeiss, Germany), pClamp (V10 and 10.7, Molecular Devices, CA, USA), Prizmatix Pulser (V3.2.1 Prizmatics Ltd., Israel).
Data analysis	GraphPad Prism (v. 9.5.1, GraphPad Software, San Diego, CA), Spike2 software (V10, Cambridge Electrical Design, Cambridge, UK), ImageJ software (v. 2.14.0/1.54f, NIH, USA), Photoshop (V12.1, CS5, Adobe, USA), Clampfit (10.7, Molecular Devices, CA, USA), MiniAnalysis (v. 6.0.3, Synaptosoft, USA), ZEN imaging software (V2.6, blue edition, Zeiss, Germany).

For manuscripts utilizing custom algorithms or software that are central to the research but not yet described in published literature, software must be made available to editors and reviewers. We strongly encourage code deposition in a community repository (e.g. GitHub). See the Nature Portfolio guidelines for submitting code & software for further information.

Data

Policy information about availability of data

All manuscripts must include a data availability statement. This statement should provide the following information, where applicable:

- Accession codes, unique identifiers, or web links for publicly available datasets
- A description of any restrictions on data availability
- For clinical datasets or third party data, please ensure that the statement adheres to our policy

All individual data are provided with this paper, and the complete dataset is available in the Source Data files associated with each corresponding Figure and

Extended Data Figure. Brain region identification was guided using either the Paxinos and Watson rat brain atlas (Paxinos G, Watson C. The Rat Brain in Stereotaxic Coordinates, 7th Edition, Academic Press, 2013) or the Paxinos and Franklin mouse brain atlas (Paxinos G, Franklin KBJ. The Mouse Brain in Stereotaxic Coordinates, 5th Edition, Academic Press, 2019), depending on the species used. These are published anatomical references and not publicly accessible datasets.

Research involving human participants, their data, or biological material

Policy information about studies with human participants or human data. See also policy information about sex, gender (identity/presentation), and sexual orientation and race, ethnicity and racism.

Reporting on sex and gender	N/A
Reporting on race, ethnicity, or other socially relevant groupings	N/A
Population characteristics	N/A
Recruitment	N/A
Ethics oversight	N/A

Note that full information on the approval of the study protocol must also be provided in the manuscript.

Field-specific reporting

Please select the one below that is the best fit for your research. If you are not sure, read the appropriate sections before making your selection.

☒ Life sciences ☐ Behavioural & social sciences ☐ Ecological, evolutionary & environmental sciences

For a reference copy of the document with all sections, see nature.com/documents/nr-reporting-summary-flat.pdf

Life sciences study design

All studies must disclose on these points even when the disclosure is negative.

Sample size	No statistical methods were used to pre-determine sample sizes. Our sample sizes were determined based on previous publications in the field and on years of experience using experimental preparations and protocols similar to those used in this study. Specifically: For optogenetics experiments: Menuet et al., eLife, 2020, Knobloch et al., Neuron, 2012; For Working Heart–Brainstem Preparation experiments: Menuet et al., eLife, 2020, Farmer et al., J Physiol, 2016; For chemogenetics experiments: Dyavanapalli et al., JACC, 2020, Grund et al., Psychoneuroendocrinology, 2019; For in vitro electrophysiology recordings: Chevalier et al., eLife, 2016, Cabirol et al., eLife, 2022; For anatomical and RNAscope experiments: Menuet et al., Cell Metabolism, 2017, Connelly et al., Neuroscience, 2025.<br>This research comply with the 3Rs rule on reducing, replacing and refining the use of animals for scientific purpose. The simple size (n) for each experiment is provided in the figure legends.
Data exclusions	In experiments where multiple parameters were recorded, if technical issues prevented the analysis of some parameters while other parameters were valid, then only the parameters with technical issues were excluded. Animals were excluded only if injection site, virus expression, or optical fiber placement were inaccurate.
Replication	All experiments were replicated across animals from different litters ("n" values are provided for each experiment). The effects of optogenetic and chemogenetic manipulations were also replicated within animals, and each individual data is an average of multiple trials within each animal (as indicated the method). Optogenetic manipulations were tested at least in three trials per animal, and chemogenetic manipulations in three trials per animal. All replicates were successful and yielded consistent results. Some experiments were replicated across species (mice and rats), and orthogonal approaches were used to validate results across ages (newborn, juvenile and adults).
Randomization	Animals were randomly selected within the pool with appropriate genotype from each litter, and randomly assigned to experimental/control groups.
Blinding	No blinding was used during data collection. Most experiments involved viral injections or surgical implantation of telemetric devices, which required close postoperative monitoring and handling by the experimenter. As the experimenter had to enter the animal facility to perform daily health checks, it was not possible to hide the genotype information, which made blinding impractical. However, outcome measures were acquired using objective and automated methods, minimizing the risk of experimenter bias. Data analysis was not blinded but all physiological data were analysed by at least 2 distinct investigators, and all analysis were performed before application of inclusion/exclusion criteria to the data (e.g. location of fluorescent beads in injectates, virus expression, fiber optic tracts etc.). Strict and predefined analysis protocols were followed, as detailed in the Methods section, to reduce subjective interpretation and ensure consistency and reproducibility across experiments.

Behavioural & social sciences study design

All studies must disclose on these points even when the disclosure is negative.

Study description	*Briefly describe the study type including whether data are quantitative, qualitative, or mixed-methods (e.g. qualitative cross-sectional, quantitative experimental, mixed-methods case study).*
Research sample	*State the research sample (e.g. Harvard university undergraduates, villagers in rural India) and provide relevant demographic information (e.g. age, sex) and indicate whether the sample is representative. Provide a rationale for the study sample chosen. For studies involving existing datasets, please describe the dataset and source.*
Sampling strategy	*Describe the sampling procedure (e.g. random, snowball, stratified, convenience). Describe the statistical methods that were used to predetermine sample size OR if no sample-size calculation was performed, describe how sample sizes were chosen and provide a rationale for why these sample sizes are sufficient. For qualitative data, please indicate whether data saturation was considered, and what criteria were used to decide that no further sampling was needed.*
Data collection	*Provide details about the data collection procedure, including the instruments or devices used to record the data (e.g. pen and paper, computer, eye tracker, video or audio equipment) whether anyone was present besides the participant(s) and the researcher, and whether the researcher was blind to experimental condition and/or the study hypothesis during data collection.*
Timing	*Indicate the start and stop dates of data collection. If there is a gap between collection periods, state the dates for each sample cohort.*
Data exclusions	*If no data were excluded from the analyses, state so OR if data were excluded, provide the exact number of exclusions and the rationale behind them, indicating whether exclusion criteria were pre-established.*
Non-participation	*State how many participants dropped out/declined participation and the reason(s) given OR provide response rate OR state that no participants dropped out/declined participation.*
Randomization	*If participants were not allocated into experimental groups, state so OR describe how participants were allocated to groups, and if allocation was not random, describe how covariates were controlled.*

Ecological, evolutionary & environmental sciences study design

All studies must disclose on these points even when the disclosure is negative.

Study description	*Briefly describe the study. For quantitative data include treatment factors and interactions, design structure (e.g. factorial, nested, hierarchical), nature and number of experimental units and replicates.*
Research sample	*Describe the research sample (e.g. a group of tagged Passer domesticus, all Stenocereus thurberi within Organ Pipe Cactus National Monument), and provide a rationale for the sample choice. When relevant, describe the organism taxa, source, sex, age range and any manipulations. State what population the sample is meant to represent when applicable. For studies involving existing datasets, describe the data and its source.*
Sampling strategy	*Note the sampling procedure. Describe the statistical methods that were used to predetermine sample size OR if no sample-size calculation was performed, describe how sample sizes were chosen and provide a rationale for why these sample sizes are sufficient.*
Data collection	*Describe the data collection procedure, including who recorded the data and how.*
Timing and spatial scale	*Indicate the start and stop dates of data collection, noting the frequency and periodicity of sampling and providing a rationale for these choices. If there is a gap between collection periods, state the dates for each sample cohort. Specify the spatial scale from which the data are taken*
Data exclusions	*If no data were excluded from the analyses, state so OR if data were excluded, describe the exclusions and the rationale behind them, indicating whether exclusion criteria were pre-established.*
Reproducibility	*Describe the measures taken to verify the reproducibility of experimental findings. For each experiment, note whether any attempts to repeat the experiment failed OR state that all attempts to repeat the experiment were successful.*
Randomization	*Describe how samples/organisms/participants were allocated into groups. If allocation was not random, describe how covariates were controlled. If this is not relevant to your study, explain why.*
Blinding	*Describe the extent of blinding used during data acquisition and analysis. If blinding was not possible, describe why OR explain why blinding was not relevant to your study.*

Did the study involve field work? ☐ Yes ☐ No

Field work, collection and transport

Field conditions	*Describe the study conditions for field work, providing relevant parameters (e.g. temperature, rainfall).*
Location	*State the location of the sampling or experiment, providing relevant parameters (e.g. latitude and longitude, elevation, water depth).*
Access & import/export	*Describe the efforts you have made to access habitats and to collect and import/export your samples in a responsible manner and in compliance with local, national and international laws, noting any permits that were obtained (give the name of the issuing authority, the date of issue, and any identifying information).*
Disturbance	*Describe any disturbance caused by the study and how it was minimized.*

Reporting for specific materials, systems and methods

We require information from authors about some types of materials, experimental systems and methods used in many studies. Here, indicate whether each material, system or method listed is relevant to your study. If you are not sure if a list item applies to your research, read the appropriate section before selecting a response.

Materials & experimental systems

n/a	Involved in the study	
☐	☒	Antibodies
☒	☐	Eukaryotic cell lines
☒	☐	Palaeontology and archaeology
☐	☒	Animals and other organisms
☒	☐	Clinical data
☒	☐	Dual use research of concern
☒	☐	Plants

Methods

n/a	Involved in the study	
☒	☐	ChIP-seq
☒	☐	Flow cytometry
☒	☐	MRI-based neuroimaging

Antibodies

Antibodies used

Primary antibodies :
1. Rabbit anti-CGRP; Sigma (C8198, lot 098M4896V).
2. Rabbit anti-NK1R; Sigma (S8305, lot 068M4878V).
3. Mouse anti-Tyrosine Hydroxylase, clone LNC1; Merk Millipore (MAB318, lot 3083054).
4. Goat anti-Choline Acetyltransferase; Merk Millipore (AB144P, lot 3475626).
5. Rabbit anti-Glutamine synthetase; Sigma (G2781, lot 0000098154).
6. Chicken anti-GFP; Avès (1020, lot GFP3717982).
7. Rabbit anti-DsRed; Clontech (632496, lot 1904182).
8. Goat anti-mCherry; Sicgen antibodies (AB0040-200, lot 0081030119).
9. Mouse anti-NeuN, clone 350D3; Synaptic System (266011, lot 266011/1-1).
10. Rabbit anti-CTB; Invitrogen (PA1-25635, lot UD2761176E).
11. Rabbit anti-µOpioid Receptor, clone UMB3; Abcam (Ab134054, lot GR323331-20).
12. Goat anti-BCHE; Biotechne (AF9024, lot CDJH0120111).
13. Rabbit anti-Calbindin; Swant (CB-38a, lot 9.03).
14. Rabbit anti-Oxytocin receptor; Alomone labs (AVR-013, lot AVR013AN0302).
15. Mouse anti-HA tag, clone 600; Cell Signaling Technology (2367S, lot 5).
16. Mouse anti-Phox2b, clone B-11; Santa Cruz (sc-376997, lot B0520).
17. Mouse anti-PS38 (H.Gainer).

Secondary antibodies:
1. Donkey anti-rabbit Alexa 488-conjugated; Invitrogen (A21206, lot 2289872).
2. Donkey anti-chicken Alexa 488-conjugated; Jackson (703545155, lot 158347).
3. Donkey anti-goat Alexa 488-conjugated; Invitrogen (A11055, lot 2301114).
4. Donkey anti-mouse Alexa 488-conjugated; Invitrogen (A21202, lot 2309139).
5. Donkey anti-rabbit Alexa 555-conjugated; Invitrogen (A31572, lot 2286312).
6. Donkey anti-goat Alexa 555-conjugated; Invitrogen (A21432, lot 2400919).
7. Donkey anti-mouse Alexa 555-conjugated; Invitrogen (A31570).
8. Donkey anti-mouse Alexa 647-conjugated; Invitrogen (A31571).
9. Donkey anti-rabbit Alexa 647-conjugated; Invitrogen (A31573, lot 2181018).
10. Donkey anti-goat Alexa 647-conjugated; Invitrogen (A21447, lot 2465096).
11. Donkey anti-mouse Alexa 647-conjugated; Invitrogen (A31571).

Validation

1. Rabbit anti-CGRP was validated by the manufacturer with immunohistochemistry of trigeminal ganglia cell cultures at a dilution of 1:200 and with immunocytochemistry of mouse lung slices at a dilution of 1:500. This antibody was validated in mouse tissue by other groups in the literature (S.M. Iyer et al., Sci Rep, 2016; Y.-Z. Lu et al., Nature, 2024).

2. Rabbit anti-NK1R was validated by the manufacturer with immunohistochemistry in rat, and the cited reference is Hamity et al., J Comp Neurol, 2014. This antibody was also validated in rat and mouse tissue by other groups in the literature and used in recent studies in the field (Menuet et al., eLife, 2020; Forsberg et al., eLife, 2016).
3. Mouse anti-Tyrosine Hydroxylase was validated by the manufacturer: "Detect Tyrosine Hydroxylase using this Anti-Tyrosine Hydroxylase Antibody, clone LNC1 validated for use in IH, IHC(P), IP & WB with more than 85 product citations. For immunohistochemistry (paraffin): a 1:200–1:400 dilution of a previous lot was used in IH. 4% PFA-fixed, frozen sections; 4% PFA-fixed, paraffin sections 1:100 (Barrachina, M. et al., 2003). For paraffin sections, Barrachina reported successful staining with microwave citrate acid antigen recovery; however, other methods can likely be used as well." This antibody has already been used in the literature in the field on rat brain tissue (Bochorishvili et al., J Comp Neurol, 2012).
4. Goat anti-Choline Acetyltransferase was validated by the manufacturer: "Anti-Choline Acetyltransferase Antibody detects levels of ChAT and has been published and validated for use in IHC(P), IC, IH, and WB. A previous lot of this antibody was used at 1:100." This antibody was used in recent studies on rat and mouse brain tissues (Menuet et al., eLife, 2020; Veerakumar et al., Nature, 2022).
5. Rabbit anti-Glutamine synthetase was validated by the manufacturer with immunohistochemistry at a minimum working antibody dilution of 1:10,000 using formalin-fixed, paraffin-embedded sections of rat brain. Anti-Glutamine Synthetase antibody has been used for the detection and localization of GS by immunoblotting and immunohistochemistry.
6. Chicken anti-GFP was validated by the manufacturer with western blot analysis (1:5000 dilution) and immunohistochemistry (1:500 dilution) using transgenic mice expressing the GFP gene product. Western blots were performed using BlokHen® (Aves Labs) as the blocking reagent, and HRP-labeled goat anti-chicken antibodies (Aves Labs, Cat. #H-1004) as the detection reagent.
7. Rabbit anti-DsRed was validated by the manufacturer using Western blot analysis on HEK-293 cells. This antibody has also been successfully used on mouse brain tissue (Krauth et al., Nat Neurosci, 2025; Jézéquel et al., Nat Commun, 2025).
8. Goat anti-mCherry was validated by the manufacturer with immunostaining on MCR cells, HEK-293 cells, and hCEC cells, and is suitable for WB, IHC(F), IHC(P), and IF applications.
9. Mouse anti-NeuN was validated by the manufacturer with immunostaining at 1:500 of PFA-fixed mouse hippocampus sections.
10. Rabbit anti-CTB was validated by the manufacturer on bacteria samples with ELISA. This antibody was also validated in mouse brain tissue by other groups in the literature (Cavalieri et al., eLife, 2021).
11. Rabbit anti-µOpioid Receptor was validated using immunohistochemistry, immunoprecipitation, and Western blot analysis on HEK-293 cells and on DRG tissue from MOR-deficient mice (PMID 20851148).
12. Goat anti-BCHE was validated by the manufacturer with immunohistochemistry in perfusion-fixed frozen sections of mouse brain and Western blot of mouse heart and liver tissues.
13. Rabbit anti-Calbindin antibodies were validated in Veerakumar et al., Nature, 2022 with immunostaining at 1:8000 on mouse brain tissue.
14. Rabbit anti-Oxytocin receptor was validated by the manufacturer with Western blot analysis of rat brain membrane (1:200), rat testis and mouse testis lysates (1:400), and with immunohistochemistry of perfusion-fixed frozen rat brain sections (1:200).
15. Mouse anti-HA tag was validated by the manufacturer with immunohistochemical analysis of paraffin-embedded COS cells and by our lab by immunostaining on mouse brain tissue.
16. Mouse anti-Phox2b was validated by the manufacturer by Western blot analysis in SK-N-SH, IMR-32, and Neuro-2A whole cell lysates. This antibody is recommended for detection of Phox2b of mouse, rat, and human origin by Western blotting (starting dilution 1:100, dilution range 1:100–1:1000), immunoprecipitation [1–2 µg per 100–500 µg of total protein (1 ml of cell lysate)], immunofluorescence (starting dilution 1:50, dilution range 1:50–1:500), and solid-phase ELISA (starting dilution 1:30, dilution range 1:30–1:3000).
17. Mouse anti-PS38 was validated by H. Gainer's lab and our lab on mouse brain tissues.

Eukaryotic cell lines

Policy information about cell lines and Sex and Gender in Research

Cell line source(s)

State the source of each cell line used and the sex of all primary cell lines and cells derived from human participants or vertebrate models.

Authentication

Describe the authentication procedures for each cell line used OR declare that none of the cell lines used were authenticated.

Mycoplasma contamination

Confirm that all cell lines tested negative for mycoplasma contamination OR describe the results of the testing for mycoplasma contamination OR declare that the cell lines were not tested for mycoplasma contamination.

Commonly misidentified lines
(See ICLAC register)

Name any commonly misidentified cell lines used in the study and provide a rationale for their use.

Palaeontology and Archaeology

Specimen provenance

Provide provenance information for specimens and describe permits that were obtained for the work (including the name of the issuing authority, the date of issue, and any identifying information). Permits should encompass collection and, where applicable, export.

Specimen deposition

Indicate where the specimens have been deposited to permit free access by other researchers.

Dating methods

If new dates are provided, describe how they were obtained (e.g. collection, storage, sample pretreatment and measurement), where they were obtained (i.e. lab name), the calibration program and the protocol for quality assurance OR state that no new dates are provided.

☐ Tick this box to confirm that the raw and calibrated dates are available in the paper or in Supplementary Information.

Ethics oversight | *Identify the organization(s) that approved or provided guidance on the study protocol, OR state that no ethical approval or guidance was required and explain why not.*

Note that full information on the approval of the study protocol must also be provided in the manuscript.

Animals and other research organisms

Policy information about studies involving animals; ARRIVE guidelines recommended for reporting animal research, and Sex and Gender in Research

Laboratory animals	Mice and rats were used in this study: C57BL6/J mice (Charles River), Swiss mice (Janvier labs), OT::cre mice (JAX stock #024234, Jackson Laboratory), Ai27(LSL-ChR2) mice (JAX stock #012567, Jackson Laboratory), OXTR::cre mice (JAX stock #031303, Jackson Laboratory), Ai14(LSL-tdTomato) mice (JAX Stock 007914, Jackson Laboratory), GAD67::EGFP mice (provided by A.Baude), GlyT2::EGFP mice (Zeilhofer lab, ETH Zurich), OT::GFP mice (GENSAT project), R26-LSL-hM4Di-DREADD mice (JAX stock #026219, Jackson Laboratory), Wistar rats (Janvier Labs, 21-30 days-old). All mice were used at 2/4-month-old, except Swiss mice (0-5 daysold). All animal experiments were conducted with animals group-housed under a 12-h light-dark cycle, at a constant temperature (22 ± 1° C) and humidity (55±10 %), with ad libitum access to food and water. Animals were fed a standard chow (SAFE R0325, SAFE Diets, France) with an approximate composition of 21.4 % crude protein, 5.1 % fat, 4.0 % crude fiber, and 52.0 % nitrogen free extract (energy content ~14.2 MJ/kg). All efforts were made to reduce animal suffering and minimize the number of experimental animals.
Wild animals	No wild animals were used.
Reporting on sex	Both sexes were used in this study. Female and male mice had responses of similar profiles and magnitudes (Fig. 2c, Extended Data Fig. 3a). Thus, female and/or male mice were used in all cohorts throughout this study.
Field-collected samples	No field samples were collected.
Ethics oversight	Animals were handled and cared for in accordance with the Guide for the Care and Use of Laboratory Animals (N.R.C., 1996) and the European Communities Council Directive of September 22th 2010 (2010/63/EU,74). Experimental protocols received approval from the institutional Ethical Committee #014 of the French Ministry of Higher Education, Research and Innovation (authorization APAFIS #12006 and #44474).

Note that full information on the approval of the study protocol must also be provided in the manuscript.

Clinical data

Policy information about clinical studies

All manuscripts should comply with the ICMJE guidelines for publication of clinical research and a completed CONSORT checklist must be included with all submissions.

Clinical trial registration	*Provide the trial registration number from ClinicalTrials.gov or an equivalent agency.*
Study protocol	*Note where the full trial protocol can be accessed OR if not available, explain why.*
Data collection	*Describe the settings and locales of data collection, noting the time periods of recruitment and data collection.*
Outcomes	*Describe how you pre-defined primary and secondary outcome measures and how you assessed these measures.*

Dual use research of concern

Policy information about dual use research of concern

Hazards

Could the accidental, deliberate or reckless misuse of agents or technologies generated in the work, or the application of information presented in the manuscript, pose a threat to:

No	Yes	
☐	☐	Public health
☐	☐	National security
☐	☐	Crops and/or livestock
☐	☐	Ecosystems
☐	☐	Any other significant area

Experiments of concern

Does the work involve any of these experiments of concern:

No	Yes	
☐	☐	Demonstrate how to render a vaccine ineffective
☐	☐	Confer resistance to therapeutically useful antibiotics or antiviral agents
☐	☐	Enhance the virulence of a pathogen or render a nonpathogen virulent
☐	☐	Increase transmissibility of a pathogen
☐	☐	Alter the host range of a pathogen
☐	☐	Enable evasion of diagnostic/detection modalities
☐	☐	Enable the weaponization of a biological agent or toxin
☐	☐	Any other potentially harmful combination of experiments and agents

Plants

Seed stocks

Report on the source of all seed stocks or other plant material used. If applicable, state the seed stock centre and catalogue number. If plant specimens were collected from the field, describe the collection location, date and sampling procedures.

Novel plant genotypes

Describe the methods by which all novel plant genotypes were produced. This includes those generated by transgenic approaches, gene editing, chemical/radiation-based mutagenesis and hybridization. For transgenic lines, describe the transformation method, the number of independent lines analyzed and the generation upon which experiments were performed. For gene-edited lines, describe the editor used, the endogenous sequence targeted for editing, the targeting guide RNA sequence (if applicable) and how the editor was applied.

Authentication

Describe any authentication procedures for each seed stock used or novel genotype generated. Describe any experiments used to assess the effect of a mutation and, where applicable, how potential secondary effects (e.g. second site T-DNA insertions, mosiacism, off-target gene editing) were examined.

ChIP-seq

Data deposition

☐ Confirm that both raw and final processed data have been deposited in a public database such as GEO.

☐ Confirm that you have deposited or provided access to graph files (e.g. BED files) for the called peaks.

Data access links
May remain private before publication.

For "Initial submission" or "Revised version" documents, provide reviewer access links. For your "Final submission" document, provide a link to the deposited data.

Files in database submission

Provide a list of all files available in the database submission.

Genome browser session
(e.g. UCSC)

Provide a link to an anonymized genome browser session for "Initial submission" and "Revised version" documents only, to enable peer review. Write "no longer applicable" for "Final submission" documents.

Methodology

Replicates

Describe the experimental replicates, specifying number, type and replicate agreement.

Sequencing depth

Describe the sequencing depth for each experiment, providing the total number of reads, uniquely mapped reads, length of reads and whether they were paired- or single-end.

Antibodies

Describe the antibodies used for the ChIP-seq experiments; as applicable, provide supplier name, catalog number, clone name, and lot number.

Peak calling parameters

Specify the command line program and parameters used for read mapping and peak calling, including the ChIP, control and index files used.

Data quality

Describe the methods used to ensure data quality in full detail, including how many peaks are at FDR 5% and above 5-fold enrichment.

Software

Describe the software used to collect and analyze the ChIP-seq data. For custom code that has been deposited into a community repository, provide accession details.

Flow Cytometry

Plots

Confirm that:

- [ ] The axis labels state the marker and fluorochrome used (e.g. CD4-FITC).
- [ ] The axis scales are clearly visible. Include numbers along axes only for bottom left plot of group (a 'group' is an analysis of identical markers).
- [ ] All plots are contour plots with outliers or pseudocolor plots.
- [ ] A numerical value for number of cells or percentage (with statistics) is provided.

Methodology

Sample preparation	*Describe the sample preparation, detailing the biological source of the cells and any tissue processing steps used.*
Instrument	*Identify the instrument used for data collection, specifying make and model number.*
Software	*Describe the software used to collect and analyze the flow cytometry data. For custom code that has been deposited into a community repository, provide accession details.*
Cell population abundance	*Describe the abundance of the relevant cell populations within post-sort fractions, providing details on the purity of the samples and how it was determined.*
Gating strategy	*Describe the gating strategy used for all relevant experiments, specifying the preliminary FSC/SSC gates of the starting cell population, indicating where boundaries between "positive" and "negative" staining cell populations are defined.*

- [ ] Tick this box to confirm that a figure exemplifying the gating strategy is provided in the Supplementary Information.

Magnetic resonance imaging

Experimental design

Design type	*Indicate task or resting state; event-related or block design.*
Design specifications	*Specify the number of blocks, trials or experimental units per session and/or subject, and specify the length of each trial or block (if trials are blocked) and interval between trials.*
Behavioral performance measures	*State number and/or type of variables recorded (e.g. correct button press, response time) and what statistics were used to establish that the subjects were performing the task as expected (e.g. mean, range, and/or standard deviation across subjects).*

Acquisition

Imaging type(s)	*Specify: functional, structural, diffusion, perfusion.*
Field strength	*Specify in Tesla*
Sequence & imaging parameters	*Specify the pulse sequence type (gradient echo, spin echo, etc.), imaging type (EPI, spiral, etc.), field of view, matrix size, slice thickness, orientation and TE/TR/flip angle.*
Area of acquisition	*State whether a whole brain scan was used OR define the area of acquisition, describing how the region was determined.*

Diffusion MRI ☐ Used ☐ Not used

Preprocessing

Preprocessing software	*Provide detail on software version and revision number and on specific parameters (model/functions, brain extraction, segmentation, smoothing kernel size, etc.).*
Normalization	*If data were normalized/standardized, describe the approach(es): specify linear or non-linear and define image types used for transformation OR indicate that data were not normalized and explain rationale for lack of normalization.*
Normalization template	*Describe the template used for normalization/transformation, specifying subject space or group standardized space (e.g. original Talairach, MNI305, ICBM152) OR indicate that the data were not normalized.*
Noise and artifact removal	*Describe your procedure(s) for artifact and structured noise removal, specifying motion parameters, tissue signals and physiological signals (heart rate, respiration).*

Volume censoring | *Define your software and/or method and criteria for volume censoring, and state the extent of such censoring.*

Statistical modeling & inference

Model type and settings | *Specify type (mass univariate, multivariate, RSA, predictive, etc.) and describe essential details of the model at the first and second levels (e.g. fixed, random or mixed effects; drift or auto-correlation).*

Effect(s) tested | *Define precise effect in terms of the task or stimulus conditions instead of psychological concepts and indicate whether ANOVA or factorial designs were used.*

Specify type of analysis: ☐ Whole brain ☐ ROI-based ☐ Both

Statistic type for inference | *Specify voxel-wise or cluster-wise and report all relevant parameters for cluster-wise methods.*

(See Eklund et al. 2016)

Correction | *Describe the type of correction and how it is obtained for multiple comparisons (e.g. FWE, FDR, permutation or Monte Carlo).*

Models & analysis

n/a	Involved in the study
☐	☐ Functional and/or effective connectivity
☐	☐ Graph analysis
☐	☐ Multivariate modeling or predictive analysis

Functional and/or effective connectivity | *Report the measures of dependence used and the model details (e.g. Pearson correlation, partial correlation, mutual information).*

Graph analysis | *Report the dependent variable and connectivity measure, specifying weighted graph or binarized graph, subject- or group-level, and the global and/or node summaries used (e.g. clustering coefficient, efficiency, etc.).*

Multivariate modeling and predictive analysis | *Specify independent variables, features extraction and dimension reduction, model, training and evaluation metrics.*
